# A Survey of the ATP-Binding Cassette (ABC) Gene Superfamily in the Salmon Louse (*Lepeophtheirus salmonis*)

**DOI:** 10.1371/journal.pone.0137394

**Published:** 2015-09-29

**Authors:** Greta Carmona-Antoñanzas, Stephen N. Carmichael, Jan Heumann, John B. Taggart, Karim Gharbi, James E. Bron, Michaël Bekaert, Armin Sturm

**Affiliations:** 1 Institute of Aquaculture, University of Stirling, Stirling FK9 4LA, Scotland, United Kingdom; 2 Edinburgh Genomics, Ashworth Laboratories, King’s Buildings, University of Edinburgh, Edinburgh EH9 3JT, Scotland, United Kingdom; University of Siena, ITALY

## Abstract

Salmon lice, *Lepeophtheirus salmonis* (Krøyer, 1837), are fish ectoparasites causing significant economic damage in the mariculture of Atlantic salmon, *Salmo salar* Linnaeus, 1758. The control of *L*. *salmonis* at fish farms relies to a large extent on treatment with anti-parasitic drugs. A problem related to chemical control is the potential for development of resistance, which in *L*. *salmonis* is documented for a number of drug classes including organophosphates, pyrethroids and avermectins. The ATP-binding cassette (ABC) gene superfamily is found in all biota and includes a range of drug efflux transporters that can confer drug resistance to cancers and pathogens. Furthermore, some ABC transporters are recognised to be involved in conferral of insecticide resistance. While a number of studies have investigated ABC transporters in *L*. *salmonis*, no systematic analysis of the ABC gene family exists for this species. This study presents a genome-wide survey of ABC genes in *L*. *salmonis* for which, ABC superfamily members were identified through homology searching of the *L*. *salmonis* genome. In addition, ABC proteins were identified in a reference transcriptome of the parasite generated by high-throughput RNA sequencing (RNA-seq) of a multi-stage RNA library. Searches of both genome and transcriptome allowed the identification of a total of 33 genes / transcripts coding for ABC proteins, of which 3 were represented only in the genome and 4 only in the transcriptome. Eighteen sequences were assigned to ABC subfamilies known to contain drug transporters, *i*.*e*. subfamilies B (4 sequences), C (11) and G (2). The results suggest that the ABC gene family of *L*. *salmonis* possesses fewer members than recorded for other arthropods. The present survey of the *L*. *salmonis* ABC gene superfamily will provide the basis for further research into potential roles of ABC transporters in the toxicity of salmon delousing agents and as potential mechanisms of drug resistance.

## Introduction

The large gene family of ATP-binding cassette (ABC) proteins has members in all biota. Typical ABC proteins possess transmembrane (TMD) and conserved nucleotide binding domains (NBD) and function as primary transporters in trafficking processes across biological membranes [1]. To form a functional transporter, two TMDs and two NBDs are required, which in full transporters are combined in a single polypeptide. In contrast, half-transporters consist of one TMD and one NBD and need to form homo- or heterodimers for transporter function. The wide range of substrates transported by ABC proteins includes inorganic ions, metals, sugars, amino acids, peptides, lipids and organic chemicals [1]. ABC proteins functioning as drug transporters can contribute to chemical resistance phenotypes in cancers, pathogens and pests. In tumours, multidrug resistance (MDR) is defined as the reduced susceptibility of cancer cells to structurally and functionally unrelated cytostatic drugs and can result from the enhanced expression of ABC efflux transporters, which reduces cellular drug accumulation [2]. ABC transporters have also been linked to drug resistance in parasitic nematodes [3] and to pesticide resistance in insects and other arthropods [4].

In metazoans, the ABC family is divided into eight subfamilies named A to H, of which subfamilies A to G have members in humans [1]. Multidrug transporters that can cause MDR are found in subfamilies B, C and G and include ABCB1 (also called MDR1 or P-glycoprotein), ABCC1 (also known as Multidrug resistance associated protein, MRP1) and ABCG2 (also known as the breast cancer resistance protein, BCRP) [2]. In non-cancerous tissues, ABC drug transporters have physiological roles in the biochemical defence against toxicants [5]. ABC drug transporters are predominantly expressed in tissues involved in excretion and/or constituting internal or external body boundaries. At these sites, ABC drug transporters often localise to the apical membranes of polarised epithelia and endothelia, resulting in directional transport of substrates into excreta (*e*.*g*., bile fluid or urine) and out of sanctuary sites (*e*.*g*., at blood-tissue barriers).

Aquatic animals, particularly those respiring through gills, are constantly exposed to a plethora of natural and anthropogenic chemicals from the ambient water. ABC drug transporters have been characterised as factors contributing to the biochemical defence against toxicants in a number of marine and freshwater organisms [6,7]. Homologues of ABCB1 and ABCC1 in the bivalve *Mytilus californicus* [8] and homologues of ABCB1, ABCC1 and ABCG2 in the sea urchin *Stronglyocentrotus purpuratus* [9] resemble their human counterparts in substrate specificity, suggesting evolutionary conservation of ABC drug pumps. Unsurprisingly, the gene complement and functional roles of ABC drug transporters are also highly similar between mammals and teleost fish [10–12]. While the ABC gene family has been annotated in the waterflea *Daphnia pulex* [13], comparatively few data exist on the roles of ABC transporters in crustaceans.

Caligid copepods, also called sea lice, are marine fish ectoparasites. In the Northern hemisphere, sea louse infections of farmed Atlantic salmon (*Salmo salar*, Linnaeus, 1758) are mostly attributable to the salmon louse (*Lepeophtheirus salmonis*, (Krøyer, 1837)) [14], for which Atlantic (*L*. *salmonis salmonis*) and Pacific sub-species (*L*. *salmonis oncorhynchi)* are recognised [15]. In addition to farm management measures and the use of cleaner fish, sea louse control relies on chemical treatments applied as baths or through feed [14]. The continuous use of medicinal agents sharing the same mode of action, however, can lead to the development of drug resistance [16]. In *L*. *salmonis*, resistance development has been reported for a number of different treatment classes including hydrogen peroxide, organophosphates, pyrethroids and avermectins [17–20].

At present, relatively little is known about the molecular mechanisms of drug resistance in *L*. *salmonis*. Changes in the expression and/or sequence of molecular target sites have similarly been suggested to contribute to decreased susceptibility of *L*. *salmonis* to organophosphates, pyrethroids and emamectin benzoate [21–23] while cytochrome P450-dependent metabolism has been proposed to affect pyrethroid toxicity in the parasite [24]. *L*. *salmonis* ABC transporters have also been suggested as factors that may potentially modulate emamectin benzoate susceptibility [20,25,26]. However, while a number of studies have investigated single *L*. *salmonis* ABC transporters, the ABC superfamily of this important parasite is at present poorly characterised.

The aim of this study was to complete a genome-wide survey of ABC genes in *L*. *salmonis*. To this end, the *L*. *salmonis* genome assembly (http://sealouse.imr.no/), which has a size of 700 Mb [27], was searched for sequences showing homology to ABC superfamily members. In addition, an *L*. *salmonis* reference transcriptome was generated by high-throughput RNA sequencing (RNA-seq) and the transcript assembly searched for ABC transcripts. The identified *L*. *salmonis* ABC transporters were characterised with regard to their evolutionary relationships to ABC transporters in other metazoan genomes.

## Materials and Methods

### Ethics statement

All research projects involving the Institute of Aquaculture (IoA) are subjected to a thorough Ethical Review Process prior to any work being approved. All projects with IoA participation are required to be submitted to the IoA Ethical Committee for approval, irrespective of where experimentation will be carried out. The forms to be completed for the ethical review process require all aspects of the experimentation to be described including conditions for the human experimenters as well as animal subjects. This procedure ensures all ethical issues are addressed before an experiment can be initiated. The present research was assessed by the IoA Ethical Review Committee and passed the Ethical Review Process of the University of Stirling.

### Salmon lice

The life cycle of *L*. *salmonis* includes non-feeding planktonic larval stages and host-associated juvenile and adult stages grazing on the host’s mucous and skin tissues [28]. Samples of different life stages of the parasite were obtained from a laboratory-maintained *L*. *salmonis* strain (IoA-00) that is susceptible to all major current salmon delousing agents. Details of *L*. *salmonis* husbandry conditions are provided elsewhere [20]. In brief, parasites were maintained on *S*. *salar* in circular tanks supplied with fresh seawater at ambient temperature, using a photoperiod corresponding to natural day length. To propagate cultures, egg strings were obtained from gravid females and allowed to develop to copepodids, which were used to infect fresh batches of host fish. Infection rates were maintained at levels that were unlikely to compromise fish welfare. Prior to the collection of *L*. *salmonis* from hosts, fish were anaesthetised with 100 mg L^-1^ 2-phenoxyethanol for 3 min. All laboratory infections were carried out under UK Home Office licence and appropriate veterinary supervision.

A total of 21 *L*. *salmonis* samples were collected, with samples corresponding to different points in the life cycle [28]. The collected material comprised egg strings, nauplius (I and II), copepodids, chalimus (I and II), as well as preadults (I and II) and adults, with some of these stages being further differentiated according to sex or age (S1 Table). Samples of nauplius, copepodid and chalimus stages consisted of pools of parasites. All samples were preserved in RNA stabilisation solution (4.54 mol L^-1^ ammonium sulphate, 25 mmol L^-1^ trisodium citrate, 20 mmol L^-1^ EDTA, pH 5.4) immediately after collection and then stored at -70°C. In order to obtain a set of samples collectively covering different points in early larval development, egg strings obtained from gravid females were incubated in aerated seawater at two temperatures (8 and 10.5°C), and in each condition nauplius larvae were collected at two time points (24 and 48 hours) by filtration. Free-living copepodids were obtained from cultures after 5 days of incubation (10.5°C).

### RNA Extraction and purification

Frozen samples were ground in liquid nitrogen using a pestle and mortar, and total RNA was immediately extracted from the homogenised sample using TRI Reagent (Sigma-Aldrich, UK), following the manufacturer’s protocol. After phase separation, RNA was precipitated from the aqueous phase by addition of 0.25 volumes isopropanol and 0.25 volumes of a high salt buffer (0.8 mol L^-1^ trisodium citrate; 1.2 mol L^-1^ sodium chloride) and resuspended in nuclease-free water. Total RNA was extracted from 21 different *L*. *salmonis* samples taken from key stages of the life cycle, where each sample consisted of pools of individuals (S1 Table). UV spectroscopy (NanoDrop ND-1000, Thermo Scientific, USA) was used to confirm purity of the RNA samples and establish concentrations, whereas RNA integrity was assessed by agarose gel electrophoresis and ethidium bromide staining.

### Library construction and sequencing

To create a total RNA pool for *L*. *salmonis* representing all key stages of the life cycle, total RNA samples were obtained from eggstring, nauplius, and copepodid stages, as well as male and female preadult (I and II) and adult stages of the parasite. To generate a representative pooled sample, 2.5 μg of total RNA from each of the 21 samples were pooled (S1 Table). This total RNA pool was further purified using RNeasy columns (Qiagen, UK). A single sequencing library was prepared from the RNA pool using Illumina TruSeq RNA Sample Prep Kit and was sequenced using an Illumina HiSeq 2000 using 100 base paired-end reads (v3 chemistry). Library preparation and sequencing was performed at the Edinburgh Genomics facility, University of Edinburgh.

### Data filtering and assemblies

Transcriptome sequencing requires high quality sequence reads for optimal assembly as sequencing errors can create difficulties for short-read assembly algorithms. We therefore performed stringent filtering to remove low-quality reads containing ambiguous bases (“N”) or with a Phred score under 20. The sequences were also screened to remove any PCR duplicates or low complexity sequences using PRINSEQ v0.20 [29]. Two complementary assembly methods were employed to process the filtered reads. First, TopHat v2.0.4 and Cufflinks [30] were used to establish a reference-based assembly using the Atlantic *L*. *salmonis* genome as a reference [http://sealouse.imr.no/] (Accessed: July 2012). Sequence reads that remained unaligned in this step were then processed using Trinity (release 2012-06-08 [31]), to generate an extra 4,144 transcripts and extend 698 reference-based transcripts. Based on the high quality reads, 37,681 transcripts (S1 Fig) were assembled. To lower the redundancy resulting from *de-novo* assemblies, all transcripts shorter than 300 bp and transcripts exhibiting tandem repeats, as detected by TRF 4.07b [32], with an entropy over 1.00 were removed. Following this step, a transcriptome of 33,933 transcripts was obtained (EBI ENA reference ERS237607), corresponding to 30,159 putative genes.

### Gene annotation and analysis

To annotate the sequences obtained, we performed sequence similarity searches using the BLAST algorithm. The longest coding DNA sequences were determined for each transcript using getorf from the EMBOSS v6.6.0 package [33]. ESTScan v2 [34] was then used to confirm transcript coding regions and determine sequence orientation. The coding sequences of the predicted transcripts were annotated using BLASTp searches against the GenBank Reference Proteins database (refseq_protein; 2014-04-07 release) from the NCBI, with an expectation value (e-value) cut-off of 10^−4^ and minimum alignment length of 33 amino acids being considered significant.

The inferred annotations were used to retrieve Gene Ontology (GO) annotation for molecular function, biological process and cellular component [35]. To avoid redundant functional assignments, the best-rated similarity hit with at least one GO annotation was chosen. A custom pipeline converted GO terms to GO Slim terms, using the Protein Information resource and Generic GO Slim files [36].

### Identification of ABC proteins

BLASTp searches were performed on predicted protein sequences of the *L*. *salmonis* genome assembly (http://sealouse.imr.no/], using the highly conserved NBD (as defined by InterPro domain IPR003439) of *Drosophila melanogaster* ABC proteins as query sequences. Hits from individual subfamily-specific BLASTp searches (E-value of 10^−5^) significantly overlapped, with each search retrieving loci of genes of the query and other subfamilies. To identify ABC superfamily members among the sequences of the *L*. *salmonis* transcriptome, the programme HMMER v3.1b1 [37] was used in connection with ABC transporter-related hidden Markov models (S2 Table). Each ABC locus identified by the above strategies was further manually annotated using BLASTp searches against the “non-redundant” sequences collection available at the NCBI. Illumina reads were mapped against cDNA sequences of ABC proteins identified in the *L*. *salmonis* transcriptome using tophat2 with standard parameters.

### Phylogenetic analyses

Phylogenetic analyses of *L*. *salmonis* ABC gene sequences also took into account ABC members from human (*Homo sapiens*) and a number of ecdysozoan invertebrates in which the ABC family has been characterised, including the nematode *Caenorhabditis elegans*, the water flea *Daphnia pulex*, the spider mite *Tetranychus urticae*, the fruit fly *Drosophila melanogaster* and the red flour beetle (*Tribolium castaneum*) [1,13,38–40]. Sequences of entire transporters, or NBDs predicted with the InterProScan tool [41], were aligned using the programme MUSCLE [42] and then subjected to phylogenetic analysis using the graphical user interphase (GUI) of the RAxML package [43,44]. The phylogenetic trees were constructed using a maximum likelihood method implementing the CAT model for heterogeneity among sites and the WAG substitution model with 1000 bootstrapping iterations.

### Data access

The raw sequence data from this study were submitted to the EBI Sequence Read Archive (SRA) study PRJEB1804. Annotated transcript sequences were deposited at the EBI European Nucleotide Archive (ENA) reference ERS237607 (contigs accession range HACA01000001-HACA01033933).

## Results and Discussion

### *L*. *salmonis* transcriptome

In order to complement searches of ABC transporters in the *L*. *salmonis* genome, a reference transcriptome of the species was generated using Illumina sequencing (RNA-seq) and searched for ABC subfamily members. In the reference transcriptome, which comprised 33,933 transcripts corresponding to 30,159 putative genes, a total of 27,086 putative genes were represented as unique transcripts while 3,073 exhibited alternatively spliced transcripts (Table 1). The absolute depth of sequencing read coverage across the full length of transcripts ranged from 1 to 519,299 reads, with an average of 795 reads across all transcripts. About 77% of the transcripts were supported by more than 10 reads and 35% were supported by more than 100 reads.

**Table 1:** Transcriptome assembly metrics.

Raw data	
Number of reads	389,927,940
Read length	101
Total size of reads	39,382,721,940
After assembly	
Number of transcripts	37,681
N50	2,060
Total size of transcripts	50,718,754
After filtering (> 300 bp)	
Number of transcripts	33,933
Number of genes	30,159
With unique transcripts	27,086
With multiple transcripts	3,073
N50	2,100
Total size of transcripts	49,850,662
Shortest transcript	300
Longest transcript	24,684
Mean size	1,469
Median size	979
Mean GC%	36.9%
N%	0.03%

The assembled transcripts were annotated using BLAST searches against the refseq_protein and UniGene databases respectively. The results indicated that out of 30,159 genes, 28,547 (95%) and 8,640 (29%) showed significant similarity to known proteins or gene transcripts in refseq_protein and UniGene databases, respectively. GO annotations were assigned to the assembled *L*. *salmonis* transcripts/genes on the basis of refseq_protein annotations. In total, 28,547 genes had similarity to known gene products; however only 4,954 (17%) were assigned GO annotations (S2 Fig).

### Identification of ABC superfamily members

A total of 33 ABC superfamily members were identified as the result of combined searches of the *L*. *salmonis* genome and reference transcriptome (Table 2, S1 File, S2 File). The identified ABC proteins comprised members of all eight metazoan ABC subfamilies A to H (Table 2). The present results suggest that *L*. *salmonis* possesses a relatively sparsely populated ABC gene superfamily, showing less members than any other arthropod previously characterised.

**Table 2:** Number of ABC superfamily members^*^ in eight eumetazoans.

ABC subfamily	*H*. *sapiens*	*C*. *elegans*	*L*. *salmonis*	*D*. *pulex*	*T*. *urticae*	*D*. *melanogaster*	*T*. *castaneum*	*A*. *mellifera*
A	12	7	3	4	9	10	10	3
B FT	4	14	1	2	2	4	2	1
B HT	7	10	3	6	2	4	4	4
C	12	9	11	8	39	14	35	9
D	4	5	3	3	2	2	2	2
E	1	1	1	1	1	1	1	1
F	3	3	4	4	4	3	3	3
G	5	9	2	24	23	15	13	15
H	0	2	5	15	22	3	3	3
TOTAL	**48**	**60**	**33**	**67**	**104**	**56**	**73**	**41**

* Numbers were derived from [1,13,38–40]. One additional ABCF transporter was identified for *T*. *urticae* (tetur11g02160). The *C*. *elegans* ABCB half transporter Abtm-1 [77] was added to the number of ABC superfamily members in this species.

Table 3 provides a list of ABC members identified in *L*. *salmonis*. Using homology searches, 30 ABC genes were identified in the *L*. *salmonis* genome while 40 transcripts encoding ABC predicted proteins were found in the *L*. *salmonis* reference transcriptome (Table 3). The 40 ABC transcripts identified corresponded to 33 putative genes, of which 7 were represented in two alternative splicing forms (Table 3). The coverage of Illumina reads to these ABC transcripts is given in the supporting information (S3 Table). The transcript Lsa.1758 corresponded to two predicted genes in the genome, while the predicted gene *augustus_masked-LSalAtl2s1361-processed-gene-0*.*2* corresponded to four (partial) sequences of the reference transcriptome. In both cases, the more comprehensive sequence was used in further analyses. Most ABC transporters were represented in both the genome and the transcriptome, three sequences were found only in the genome and four only in the transcriptome (Table 3).

**Table 3:** Summary of 33 ABC proteins identified in *L*. *salmonis*.

Subfamily	Salmon louse genome ID ^¥^	AA length	Transcript ID	AA length	Detected Topology	Best Hit in *Drosophila melanogaster*	Accession number	e-value
A	maker-LSalAtl2s118-snap-gene-1.28-mRNA-1	658 †	Lsa.1758^A^	1583	TMD_1_—NBD_1_—TMD_2_—NBD_2_	CG1718, isoform D	NP_001259765.1	2.00E-104
	maker-LSalAtl2s118-snap-gene-1.27-mRNA-1	693 †						
A	maker-LSalAtl2s725-augustus-gene-0.4-mRNA-1	99 †	Lsa.14583	127 †	TMD_1_	CG34120, isoform E	NP_001285513.1	4.00E-04
A	-	-	Lsa.1680	615 †	TMD_2_	CG1718, isoform B	NP_608445.2	4.00E-15
B	maker-LSalAtl2s2344-augustus-gene-0.12-mRNA-1	476 †	Lsa.26127^A^	566 †	TMD—NBD	CG3156	NP_569844.2	4.00E-160
B	maker-LSalAtl2s174-augustus-gene-3.18-mRNA-1^A^	1006	Lsa.7262	669	TMD—NBD	AT07502p (CG7955)	ADV19030.1	0.0
B	maker-LSalAtl2s445-augustus-gene-0.18-mRNA-1	676	Lsa.643^A^	687	TMD—NBD	CG1824	NP_572810.1	0.0
B	snap_masked-LSalAtl2s662-processed-gene-1.13-mRNA-1	779 †	Lsa.4043^A^	1448	TMD_1_—NBD_1_—TMD_2_—NBD_2_	RE14657p (mdr65)	AAM51996.1	0.0
C	maker-LSalAtl2s498-snap-gene-2.25-mRNA-1	1526	Lsa.11278^A^	1531	TMD_0_—TMD_1_—NBD_1_—TMD_2_—NBD_2_	Multidrug-Resistance like protein 1, isoform F (dMRP1/CG6214)	NP_995692.1	0.0
C	maker-LSalAtl2s1014-snap-gene-0.6-mRNA-1	883 †	Lsa.22810^A^	979 †	TMD_1_—NBD_1_—TMD_2_	Multidrug-Resistance like protein 1, isoform J (dMRP1/CG6214)	NP_995696.1	0.0
C	maker-LSalAtl2s1014-snap-gene-0.5-mRNA-1	215 †	Lsa.29272*	185 †	TMD_1_	IP16232p (CG6214)	ABM92813.1	3.00E-15
C	maker-LSalAtl2s812-augustus-gene-0.6-mRNA-1^A^	1989	Lsa.6310	1175	TMD_0_—TMD_1_—NBD_1_—TMD_2_—NBD_2_	Multidrug-Resistance like protein 1, isoform M (dMRP1/CG6214)	NP_995704.1	0.0
C	maker-LSalAtl2s1420-augustus-gene-0.12-mRNA-1	1559	Lsa.8882* ^A^	1563	TMD_0_—TMD_1_—NBD_1_—TMD_2_—NBD_2_	Multidrug-Resistance like protein 1, isoform M (dMRP1/CG6214)	NP_995704.1	0.0
C	maker-LSalAtl2s111-snap-gene-2.14-mRNA-1	1896	Lsa.23107* ^A^	1381	TMD_1_—NBD_1_—TMD_2_—NBD_2_	CG7627	NP_609215.3	0.0
C	maker-LSalAtl2s111-augustus-gene-1.10-mRNA-1	1597	Lsa.3521* ^A^	926 †	TMD_1_—NBD_1_—TMD_2_	Multidrug resistance protein 4 ortholog (CG14709)	NP_650086.2	0.0
C	-	-	Lsa.3522^A^	1293	TMD_1_—NBD_1_—TMD_2_—NBD_2_	CG9270	NP_995741.2	0.0
C	augustus_masked-LSalAtl2s128-processed-gene-18.4-mRNA-1^A^	1455	Lsa.4564	1466	TMD_0_—TMD_1_—NBD_1_—TMD_2_—NBD_2_	CG7806	NP_609207.1	0.0
C	augustus_masked-LSalAtl2s1361-processed-gene-0.2^A^	1892	Lsa.14261, Lsa.14262, Lsa.14263, Lsa.14264	198 †, 220 †, 274 †, 196 †	TMD_0_—TMD_1_—NBD_1_—TMD_2_—NBD_2_	Multidrug-Resistance like protein 1, isoform H (dMRP1/CG6214)	NP_995694.1	4.00E-91
C	augustus_masked-LSalAtl2s197-processed-gene-0.4-mRNA-1^A^	1412	-	-	TMD_0_—TMD_1_—NBD_1_—TMD_2_—NBD_2_	CG4562	NP_650838.1	0.0
D	augustus_masked-LSalAtl2s3118-processed-gene-0.0-mRNA-1^A^	589 †	-	-	TMD—NBD	Pmp70, isoform C (CG12703)	NP_001259733.1	2.00E-48
D	maker-LSalAtl2s324-augustus-gene-1.87-mRNA-1^A^	597 †	Lsa.10176	455 †	TMD—NBD	Pmp70, isoform A (CG12703)	NP_608354.1	1.00E-41
D	-	-	Lsa.5856^A^	656	TMD—NBD	Pmp70, isoform C (CG12703)	NP_001259733.1	2.00E-46
E	maker-LSalAtl2s1021-augustus-gene-0.34-mRNA-1	615	Lsa.1035* ^A^	670	NBD_1_—NBD_2_	Pixie, isoform A (CG5651)	NP_648272.1	0.0
F	maker-LSalAtl2s1166-snap-gene-0.62-mRNA-1^A^	738	Lsa.20458	109 †	NBD_1_—NBD_2_	CG1703	NP_572736.1	0.0
F	maker-LSalAtl2s530-snap-gene-0.3-mRNA-1^A^	126 †	-	-	NBD_2_	GM14873p (CG9281)	AAL39441.1	2.00E-30
F	maker-LSalAtl2s917-augustus-gene-1.21-mRNA-1	577	Lsa.9678^A^	660	NBD_1_—NBD_2_	CG9281	NP_573057.1	0.00E+00
F	maker-LSalAtl2s1-augustus-gene-35.19-mRNA-1	659	Lsa.8082^A^	758	NBD_1_—NBD_2_	CG9330	NP_649129.1	0.0
G	maker-LSalAtl2s467-augustus-gene-3.18-mRNA-1	617	Lsa.2606* ^A^	702	NBD—TMD	CG11069	NP_651307.2	7.00E-154
G	maker-LSalAtl2s467-augustus-gene-3.17-mRNA-1	978	Lsa.25615^A^	1001	NBD—TMD	CG31121	NP_733058.1	2.00E-123
H	snap_masked-LSalAtl2s1226-processed-gene-0.1-mRNA-1	676	Lsa.12984^A^	825	NBD—TMD	CG9990	NP_001247351.1	0.0
H	augustus_masked-LSalAtl2s1118-processed-gene-0.6-mRNA-1	279 †	Lsa.14023^A^	737	NBD—TMD	CG9990	NP_001189305.1	0.0
H	-	-	Lsa.21408^A^	683	NBD—TMD	CG9990	NP_001189305.1	7.00E-66
H	augustus_masked-LSalAtl2s100-processed-gene-0.5-mRNA-1^A^	740	Lsa.23267	716	NBD—TMD	CG9990	NP_001189305.1	0.0
H	maker-LSalAtl2s100-augustus-gene-0.13-mRNA-1	507 †	Lsa.23269* ^A^	773	NBD—TMD	CG9990	NP_001189305.1	0.0

n/a, not applicable; AA, amino acid; TM, transmembrane domain; NDB, nucleotide binding domain

¥ Predicted protein (Database LSalAtl2EbiPred6; http://sealouse.imr.no/).

† Partial sequence only.

* Alternative splicing forms exist.

**Fig 1:**
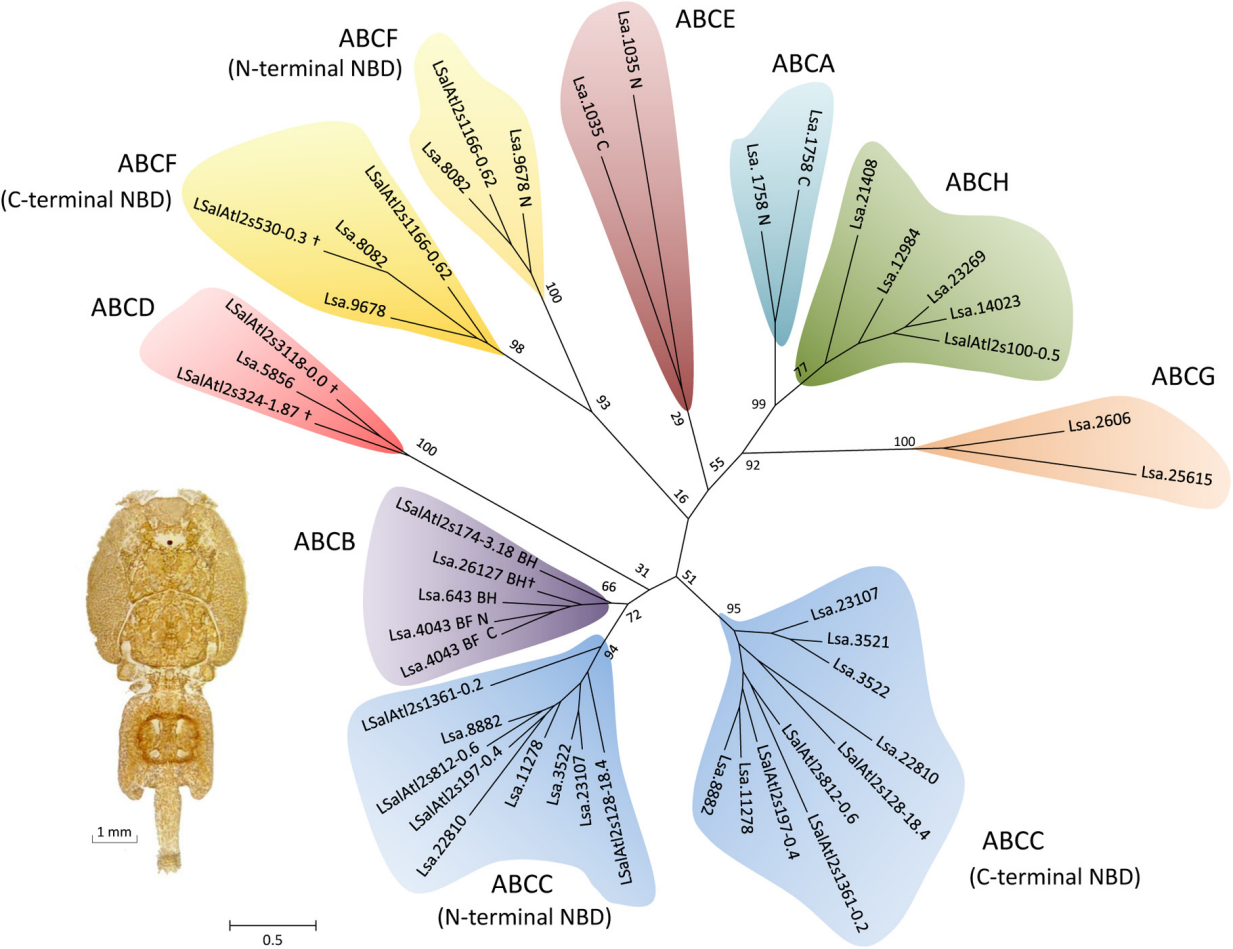
Unrooted phylogenetic tree of NBDs of 30 ABC proteins of *L*. *salmonis*. Amino acid sequences of NBDs predicted with the InterProScan tool [41] were aligned using MUSCLE [42] and subjected to a maximum likelihood analysis using RAxML [44]. For phylogenetic reconstruction, the WAG substitution model and CAT model of rate heterogeneity among sites were implemented. Numbers at the basal nodes represent the frequencies with which the presented tree topology was obtained after bootstrapping (1000 iterations). The scale bar represents 0.5 amino-acid substitutions per site. The different ABC protein subfamilies are indicated by shaded colours. Accession numbers of sequences are provided in S4 Table.

A phylogenetic analysis grouped NBDs of the *L*. *salmonis* ABC proteins into clades corresponding to known ABC subfamilies with high bootstrap support (Fig 1). The subfamily assignment was re-evaluated and in all cases confirmed based on domain architecture, presence of conserved protein motifs and manual annotation through BLASTp searches (Table 3). The evolutionary relationship of *L*. *salmonis* ABC proteins to ABC gene family members of other metazoans was elucidated in subfamily specific analyses.

#### Subfamily A

Subfamily A contains full transporters involved in the trafficking of lipid compounds [45]. Three subfamily A members were identified in *L*. *salmonis* (Table 2). Phylogenetic analyses focused on Lsa.1758, excluding the short partial sequences Lsa.14583 and Lsa.1680 (Table 3). The obtained tree is in accordance with the hypothesis that lineage-specific gene duplications occurred in most species considered (S3 Fig). *L*. *salmonis* Lsa.1758 grouped together with *D*. *pulex* dappu1_312055 and dappu1_312056, *T*. *urticae* tetur25g01640 as well as human ABCA1, ABCA2, ABCA4 and ABCA7. Little functional information is available for the A subfamily in invertebrates. The transcriptional knockdown of *T*. *castaneum* ABCA-9A/B by RNAi lead to defects in wing and elytra development [40].

#### Subfamily B

Subfamily B is the only metazoan ABC subfamily containing both half and full transporters. Thus, separate evolutionary analyses were carried for both these types.

**Fig 2:**
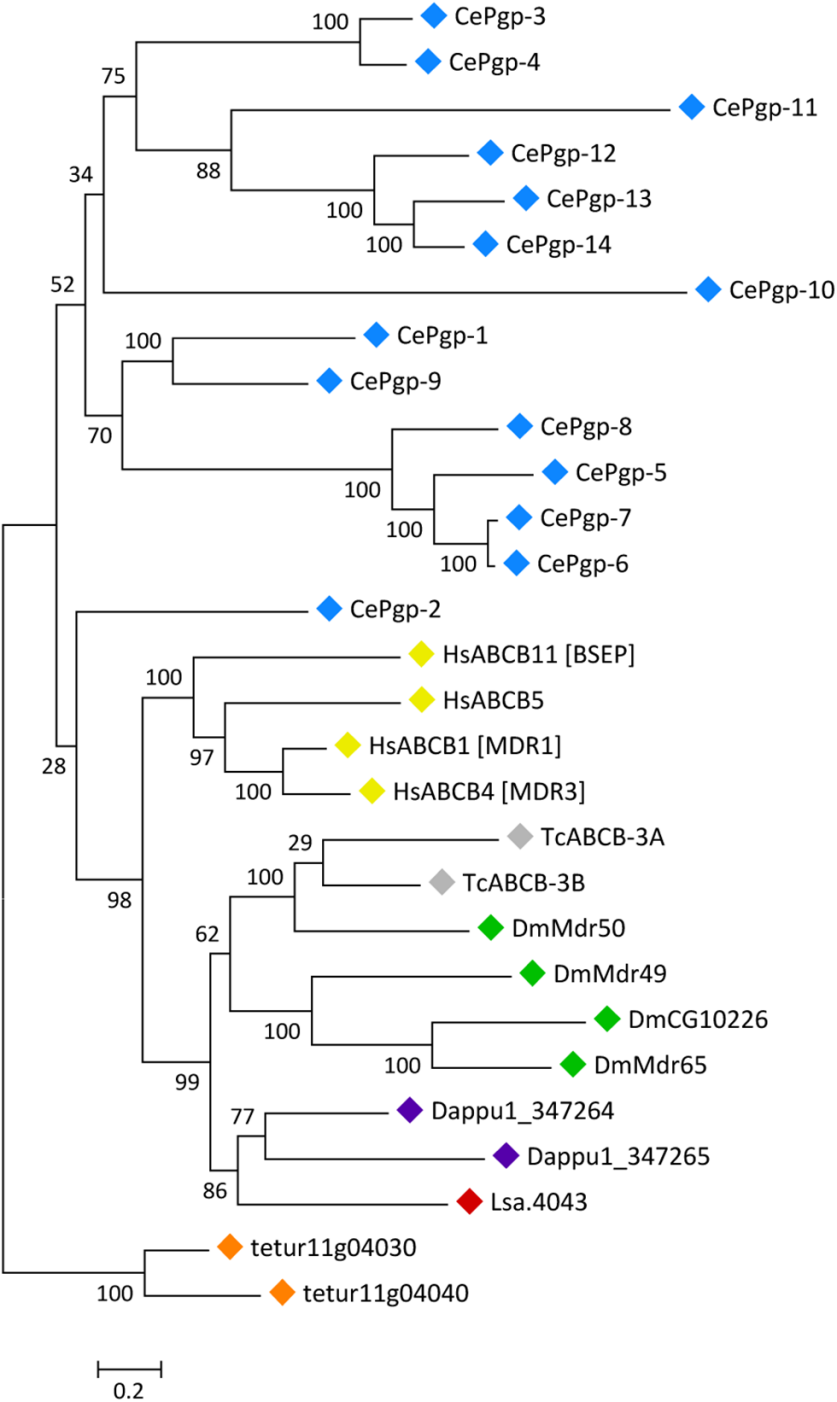
Phylogenetic analysis of ABCB full transporters of *H*. *sapiens* and six invertebrate species. Full-length coding regions were aligned using MUSCLE [42] and subjected to a maximum likelihood analysis using RAxML [44]. Colour diamonds are as follows: blue, *C*. *elegans*; yellow, *H*. *sapiens*; grey, *T*. *castaneum*; green, *D*. *melanogaster*; purple, *D*. *pulex*; orange, *T*. *urticae* and red, *L*. *salmonis*. The scale bar represents amino-acid substitutions per site. Accession numbers of used sequences are provided in S4 Table.

In humans, subfamily B full-transporters include the multidrug transporter ABCB1 (also called P-glycoprotein), as well as the biliary phospholipid pump ABCB4, the bile salt export pump ABCB11 and ABCB5, a drug-resistance mediator and regulator of the cell cycle in cancer stem cells [1,46]. The only subfamily B full-transporter sequence found in *L*. *salmonis*, Lsa.4043 (Table 2), corresponds to SL-PGY1, which has been cloned and characterised in a previous study [20]. SL-PGY1 is transcriptionally up-regulated following emamectin benzoate (EMB) exposure [20] and co-exposure to P-glycoprotein inhibitors increases the toxicity of EMB in *L*. *salmonis* [47]. While this observation is in accordance with potential roles of SL-PGY1 as a multidrug pump, further evidence supporting this hypothesis is lacking. The results from the phylogenetic analysis of B subfamily full-transporters are suggestive of extensive lineage-specific gene duplications in human, *C*. *elegans* and *T*. *urticae* (Fig 2). Lsa.4043/SL-PGY1 grouped together with *D*. *pulex* and insect proteins, including *D*. *melanogaster* mdr49, mdr50 and mdr65, in a cluster of high bootstrap support (Fig 2).

Different lines of evidence support a role for arthropod subfamily B full-transporters as multidrug pumps contributing to the biochemical defence against toxicants. Mdr49, mdr50 and mdr65 are expressed in Malphigian tubules and gut [48] while mdr65 is expressed at the humoral/CNS interface [49]. Pharmacological blockage or RNA interference of subfamily B full transporters can increase the toxicity of chemicals known to be transport substrates in insects and *Daphnia* [50–52]. Subfamily B full-transporters are commonly up-regulated following exposure to organic xenobiotics [48,52,53] and their overexpression can contribute to insecticide resistance phenotypes [54]. In addition to roles of in the biochemical defence against xenobiotics, some arthropod subfamily B full-transporters are involved in the transport of endogenous substrates [55].

Expression of subfamily B half-transporters in humans is mainly localised in intracellular membranes. ABCB6, ABCB7, ABCB8 and ABCB10 are highly conserved mitochondrial proteins and have roles in the cellular homeostasis of iron and transport of Fe/S protein precursors [1]. ABCB2/TAP1 and ABCB3/TAP2 are located in the endoplasmic reticulum and have roles in antigen processing by the major histocompatibility complex (MHC) class I, whereas ABCB9 shows a lysosomal localisation [56]. Three subfamily B half-transporters were found in *L*. *salmonis* (Table 2). The phylogenetic analysis yielded relatively clear orthologous relationships (S4 Fig). No homologues of human ABCB2, ABCB3 or ABCB9 were identified in *L*. *salmonis* or other arthropods (S4 Fig). ABCB6 showed homologues in *C*. *elegans*, *D*. *magna* and insects, but not *L*. *salmonis* or *T*. *urticae*. ABCB7, ABCB8 and ABCB10 had homologues in *L*. *salmonis*, *C*. *elegans*, *D*. *magna* and insects (S4 Fig).

The *L*. *salmonis* ABCB8 homologue Lsa.643 has been cloned and designated SL-Pgp1 in a previous study [25]. SL-Pgp1 showed transcriptional up-regulation following exposure to emamectin benzoate [25]. While B subfamily half-transporters are generally not considered multidrug pumps, an orthologue to *A*. *gambiae* ABCB4 was 2-fold to 5-fold over-transcribed in pyrethroid resistant strains of the mosquito *Aedes aegypti* [57], which also showed elevated levels of C and G subfamily members (see below).

#### Subfamily C

**Fig 3:**
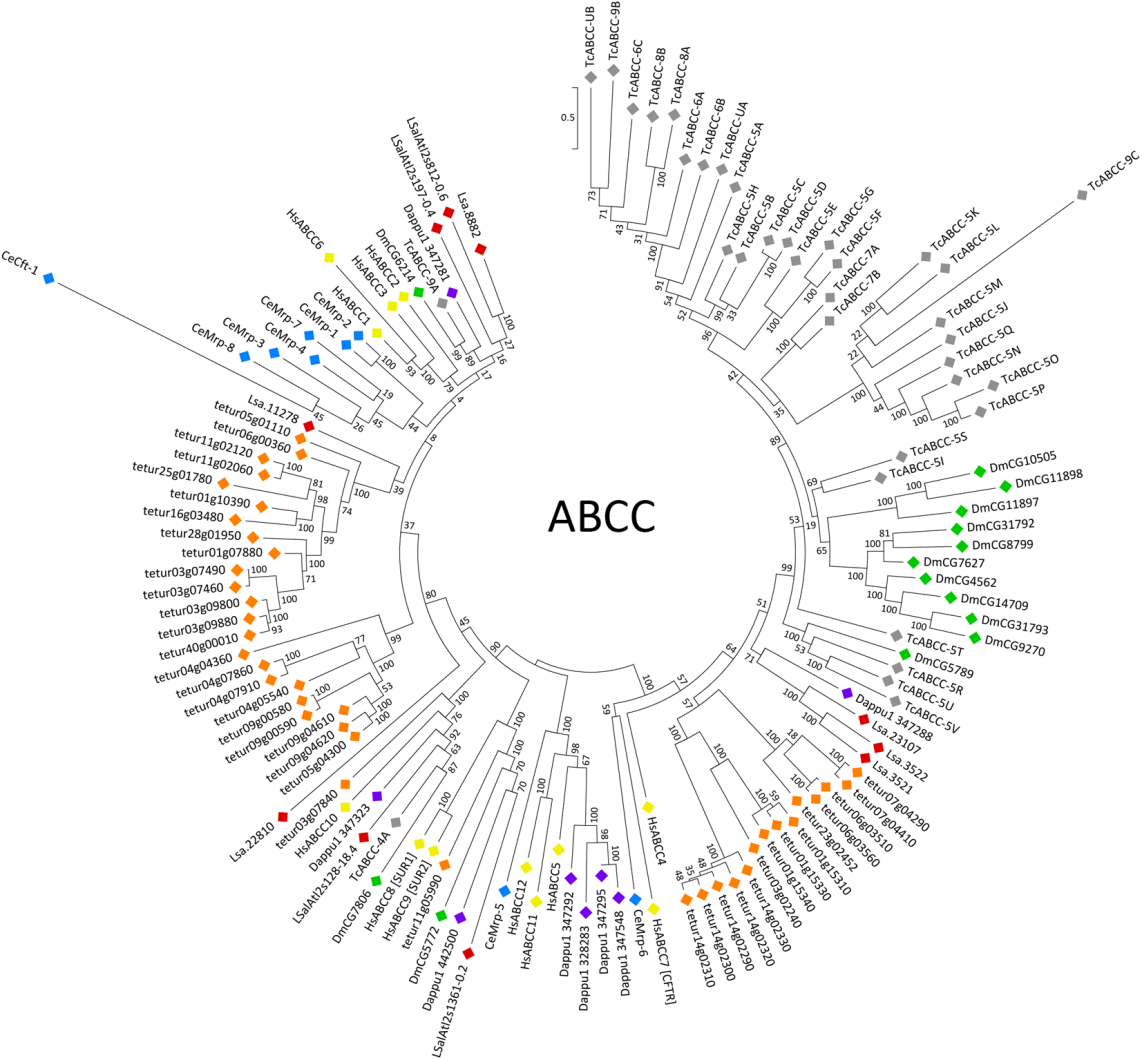
Phylogenetic analysis of ABCC transporters of *H*. *sapiens* and six invertebrate species. Full-length coding regions were aligned using MUSCLE and subjected to a maximum likelihood analysis using RAxML. For further explanations see the legend of Fig 2.

The C subfamily is functionally diverse, comprising transporters called multidrug resistance associated proteins (MRPs), a chloride channel called the cystic fibrosis transmembrane conductance regulator (CFTR, ABCC7), and the sulfonylurea receptors (SUR1/2, ABCC8/9) that function as regulators of potassium channels [58]. A total of 11 subfamily C members were identified in *L*. *salmonis* (Table 2), all of which were subjected to evolutionary analysis apart from the short partial sequence *maker-LSalAtl2s1014-snap-gene-0*.*5-mRNA-1*/Lsa.29272. The phylogenetic tree obtained shows evidence of extensive lineage-specific gene duplications in all species studied, particularly in *T*. *urticae* and *T*. *castaneum*, with few examples of clear one-to-one orthologous relationships (Fig 3).

The *L*. *salmonis* gene/transcript *augustus_masked-LSalAtl2s1361-processed-gene-0*.*2* grouped together in a clade with human and fruit fly SURs, suggesting it represents a SUR orthologue (Fig 3). As expected, no CFTR homologue was found in *L*. *salmonis* (Fig 3). CFTR is believed to have emerged following neofunctionalisation after gene duplication in the vertebrate lineage [59] so that CFTR homologues are lacking in arthropods [4].

The remaining *L*. *salmonis* subfamily C sequences lack specific homology to SUR or CFTR and therefore likely represent MRP transporters. In the phylogenetic tree of subfamily C (Fig 3), a number of *L*. *salmonis* sequences (Lsa.8882, maker-LSalAtl2s812-augustus-gene-0.6-mRNA-1, Lsa.11278, augustus_masked-LSalAtl2s197-processed-gene-0.4-mRNA-1) fall into a large clade also comprising human ABCC1-3, *C*. *elegans* mrp-1 and fruit fly CG6214. The human multidrug transporters ABCC1 (MRP1) and ABCC2 (MRP2) mediate the cellular efflux of a wide range of organic chemicals and their conjugates with glutathione, glucuronic acid and sulphate [58]. ABCC1/2 further transport free glutathione and certain metals, possibly as glutathione complexes [58]. *D*. *melanogaster* CG6214 (also called dMRP) has been shown to transport a similar range of substrates to human ABCC1 [60,61]. Similarly, *C*. *elegans* mrp-1 contributes to resistance against heavy metals and the anthelmintic ivermectin [62,63]. While the phylogenetic assignment of a number of *L*. *salmonis* sequences to one clade with the above human and invertebrate multidrug pumps (Fig 3) provides support for the hypothesis that these *L*. *salmonis* proteins adopt similar functions, the large clade containing human and invertebrate multidrug transporters also contains ABCC6, a protein of unknown function which does not show drug-transport activity [64].

In phylogenetic analyses, *L*. *salmonis augustus_masked-LSalAtl2s1361-processed-gene-0*.*2* was assigned to a well-supported clade also containing human ABCC10 (MRP7), as well as one sequence each from *D*. *pulex*, *T*. *urticae*, *D*. *melanogaster* and *T*. *castaneum* (Fig 3). The presence of putative arthropod orthologues to ABCC10 has been noticed previously [4]. While ABCC10 shows drug-transporting activity *in vitro*, further evidence for its role as a drug pump is lacking [58].

As stated above, lineage-specific expansions of the C subfamily were observed in some arthropods, including *T*. *urticae*, *T*. *castaneum* and, to a lesser extent, *D*. *melanogaster*. No functional information is available for most of these proteins. In *D*. *melanogaster*, CG10505 has roles in metal homeostasis [65], while CG14709 is involved in responses to oxidative stress [66]. In *T*. *castaneum*, knock-down of C subfamily transporter expression by dsRNA injection into penultimate larvae did not result in detectable phenotypic changes [40].

Available evidence suggests that C subfamily transporters can contribute to insecticide resistance. Constitutive transcriptional up-regulation of C subfamily members was observed, along with changed mRNA levels of other ABC proteins, in a multi-resistant *T*. *urticae* strain, a pyrethroid resistant isolate of the mosquito *Aedes aegyptii* and chlorpyrifos and fipronil resistant strains of the moth *Plutella xylostella* [39,57,67].

#### Subfamily D

The D subfamily contains half-transporters involved in the import of fatty acids and their precursors into the peroxisome [1]. Three subfamily D sequences were identified in *L*. *salmonis* (Table 2), which clustered together with human ABCD4 in the phylogenetic analysis (S5 Fig). In contrast, terrestrial arthropods (*T*. *urticae*, *D*. *melanogaster* and *T*. *castaneum*) had homologues to human ABCD1 and ABCD3 but lacked homologues to human ABCD4 (S5 Fig).

#### Subfamilies E and F

Subfamilies E and F contain atypical ABC proteins composed of a pair of linked nucleotide binding domains and lacking transmembrane domains [1]. Members of the E subfamily have central roles in translation initiation [68]. The E subfamily has one member in all metazoans studied to date [1,4], and *L*. *salmonis* conforms to this rule (Table 2, S6 Fig). Subfamily F proteins have functions in ribosome assembly and/or protein translation [69]. Four subfamily F sequences were identified in *L*. *salmonis* (Table 2). In the phylogenetic analysis, subfamily F proteins grouped in three clades containing homologues to the three human ABCF members ABCF1, ABCF2 and ABCF3, with two putative ABCF2 orthologues being present in *L*. *salmonis* (S7 Fig).

#### Subfamily G

The G subfamily contains half-transporters showing a “reverse” domain order, with the NBD being located N-terminally to the TMD [1]. Similar to “regular” ABC half-transporters, reverse half-transporters assemble functional pumps by forming homo- or heterodimers. The human G subfamily has five members, which include the multidrug efflux pump ABCG2 as well as four lipid/sterol transporters [1].

**Fig 4:**
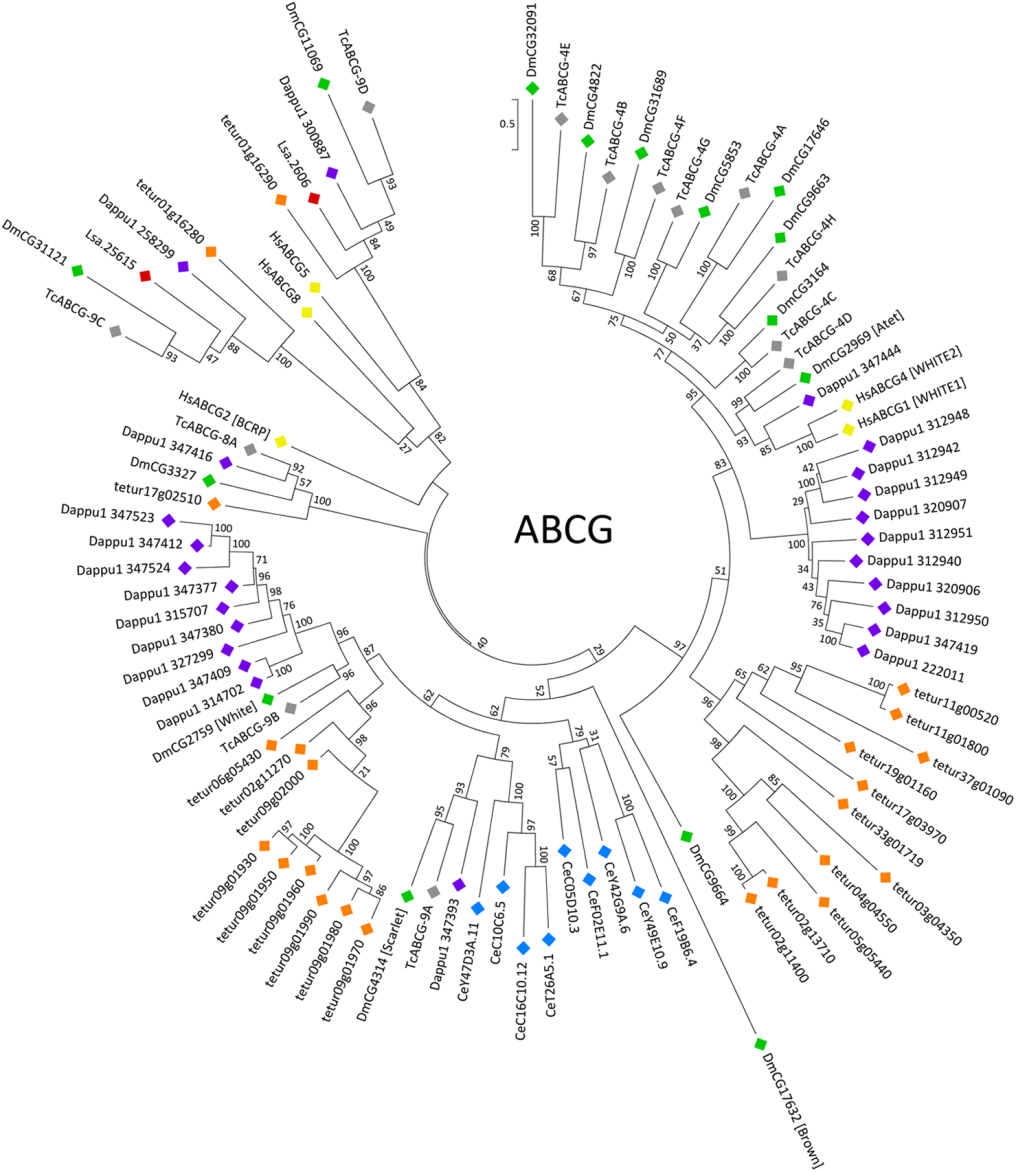
Phylogenetic analysis of ABCG transporters of *H*. *sapiens* and six invertebrate species. Full-length coding regions were aligned using MUSCLE and subjected to a maximum likelihood analysis using RAxML. For further explanations see the legend of Fig 2.

In *L*. *salmonis*, two subfamily G sequences were identified (Table 2). The phylogenetic analysis revealed extensive lineage specific gene duplications and relatively few instances of clear orthologous relationships (Fig 4).

In the obtained tree, both *L*. *salmonis* Lsa.2606 and Lsa.25615 have single orthologues in all arthropod species analysed (Fig 4). Moreover, the cluster containing Lsa.2606 further grouped together with human ABCG5 at high bootstrap support. Similarly, the clade containing Lsa.25615 associated with human ABCG8, albeit at low bootstrap support (Fig 4). In line with these observations, it has been suggested earlier that arthropods possess orthologues of human ABCG5 and ABCG8 [4]. While human ABCG5 and ABCG8 form heterodimers acting as intestinal and biliary efflux pumps for cholesterol and dietary sterols [70], the functions of putative arthropod orthologues of these pumps remain to be identified.

In the phylogenetic tree obtained in this study, a well-supported clade is further formed by *D*. *melanogaster* CG3327 (also called E23) and putative orthologues in *T*. *castaneum*, *T*. *urticae* and *D*. *pulex* (Fig 4). So far, no homologues of CG3327 have been identified in *L*. *salmonis* (this study), the mosquito *Anopheles gambiae* or the silk worm *Bombyx mori* [4].

The products of the *Drosophila* genes *white*, *brown* and *scarlet* are half-transporters with roles in the uptake of eye pigment precursors into lysosome-like vesicles in the developing fly eye [71]. The product of the *white* gene forms heterodimers with either the *brown* or *scarlet* gene products. Loss-of-function mutations of the above transporters cause distinct eye-colour phenotypes (white, brown, scarlet) in fruit fly. Most ecdysozoan invertebrates have homologues to either *white* or *scarlet* or to both (Fig 4). Surprisingly, no such homologues were identified in *L*. *salmonis* (Fig 4).

In humans, ABCG1 has roles in the homeostasis of intracellular levels of sterols, particularly cholesterol, while ABCG4 is a lipid transporter of unknown function [70]. Homologues to ABCG1 and ABCG4 are present in *Daphnia pulex* and insects, but are lacking in *L*. *salmonis* (Fig 4). For *T*. *castaneum* ABCG-4C, which is found in the sister group to the clade containing ABCG1 and ABCG4, knock-down of transporter expression by RNA interference resulted in abortive larval-pupal moulting and pupal death as well as dehydration and cuticle deficiencies [40]. This phenotype is consistent with a role of ABCG-4C as a transporter of cuticular lipids.

Human ABCG2 (also called BCRP) is a drug efflux transporter [2,5]. No putative ABCG2 orthologues were identified in *L*. *salmonis* (Fig 4). This parallels the situation in other arthropod genomes, where transporters showing specific homology to ABCG2 are absent [4].

Despite the absence of arthropod orthologues to ABCG2, some evidence suggests that insect subfamily G members may function as multidrug transporters. In a DDT resistant fruit fly strain, transcript levels CG31689 were increased [72]. Similarly, subfamily G members were among ABC transporters of different families upregulated in insecticide resistant *P*. *xylostella* and *A*. *aegypti* strains [57,67].

#### Subfamily H

Subfamily H, which was initially defined during the annotation of ABC proteins in *D*. *melanogaster* [1], is found in arthropods and nematodes [4,38]. Metazoan subfamily H and G proteins share a “reverse” half-transporter architecture. The H subfamily is lacking in mammals, but has one member in the genome of zebrafish *Danio rerio* [10].

Five members of the H subfamily were found in *L*. *salmonis* in this study (Table 2). The phylogenetic tree obtained for the ABCH subfamily suggests that lineage-specific gene duplications lead to the diversification of the H subfamily in *L*. *salmonis* (5 members), *D*. *pulex* (15 members), *T*. *urticae* (22 members) and insects (3 members) (S8 Fig).

The physiological functions of H subfamily are still poorly understood. Transcriptional silencing of the putative CG9990 orthologue ABCH-9C caused a phenotype similar to that observed in ABCG-4C knockdown beetles, suggesting that ABCH-9C may also function in the transport of cuticular lipids [40]. In the aphid *Myzus persicae*, exposure to pirimicarb resulted in the transcriptional up-regulation of a putative CG33970 orthologue [73]. Similarly, different ABC transporters including a putative CG9990 orthologue showed increased transcript levels in insecticide resistant strains of the moth *Plutella xylostella* [67].

### General features of *L*. *salmonis* ABC gene family

The present study identified ABC gene family members in the parasitic copepod *L*. *salmonis* by searches of the *L*. *salmonis* genome and a reference transcriptome of the species. While we cannot exclude the possibility that the number of ABC proteins in *L*. *salmonis* will be corrected up- or downwards should more data become available, the present results reveal some interesting features of the *L*. *salmonis* ABC superfamily. While all ABC subfamilies A to H are represented in *L*. *salmonis* (Table 2), fewer ABC proteins have been found in this species than in any other arthropod studied so far in this regard [4].

ABC transporters with well documented roles in the biochemical defence against toxicants include full-transporters of subfamily B and members of subfamilies C and G [5]. In addition, some members of subfamily H may have similar chemoprotective roles in arthropods [4]. It is worth noting that these subgroups of ABC pumps are expanded by lineage-specific gene duplications in different phytophagous arthropods, e.g. *T*. *urticae* and *T*. *castaneum* (Table 2). In contrast, ABC subgroups with highly conserved roles, such as half-transporters of subfamily B and all members of subfamilies D, E and F, show comparatively similar counts among the genomes of human, *C*. *elegans* and different arthropods (Table 2). The expansion of multidrug pumps of the ABC gene superfamily in arthropods grazing on plants parallels that of other gene families with roles in the biochemical defence and is likely to represent an adaptation to plant secondary metabolites [74]. Similar expansions of the ABC superfamily in *C*. *elegans* and *D*. *melanogaster* (Table 3) could reflect that these species are, at least for a part of their life cycle, detritivores and may experience exposure to microbial chemicals. In contrast, the honeybee *A*. *mellifera*, which maintains a mutualistic symbiotic relationship with flowering plants, possesses a relatively compact ABC superfamily (Table 2), as well as smaller genomic complements than *D*. *melanogaster* for other gene families involved in detoxification [75]. A similar trend is observed in the human body louse *Pediculus humanus*, the genome of which shows a marked reduction in the number of ABC transporters, cytochrome P450s, glutathione-S-transferases and esterases [76]. The parasitic relationship between the salmon louse and its Atlantic salmon host, whereby it is partially protected from environmental toxicants during host-attached phases of the life-cycle and ingests only host products when feeding, means that it may be able to rely, in part, upon intervening host detoxification pathways and therefore displays a reduced complement of ABC transporters.

## Conclusion

The annotation of the ABC superfamily in *L*. *salmonis* represents a significant step towards an improved understanding of potential drug resistance factors in this species and related parasites. The identification of potential drug transporters provides a basis for elucidating the roles of ABC proteins in the biochemical defence of *L*. *salmonis* against salmon delousing agents.

## Supporting Information

S1 Fig Overview of the *L. salmonis* transcriptome assembly. Size distribution of assembled transcripts (median 821 nt). Cut-off at 300 nt.
(EPS)

S2 Fig Multilevel Gene Ontology (GO) categorisation of the annotated transcripts. GO terms were assigned to 4,954 (17%) of the 28,547 annotated transcripts based on refseq_protein annotation. GO Annotations were first converted to GO-Slim annotations and the multilevel chart shows the top ten of each category to reduce the complexity of the chart.
(EPS)

S3 Fig Phylogenetic analysis of ABCA transporters. (TIF)

S4 Fig Phylogenetic analysis of ABCB half transporters. (TIF)

S5 Fig Phylogenetic analysis of ABCD transporters. (TIF)

S6 Fig Phylogenetic analysis of ABCE transporters. (TIF)

S7 Fig Phylogenetic analysis of ABCF transporters. (TIF)

S8 Fig Phylogenetic analysis of ABCH transporters. (TIF)

S1 File Predicted protein sequences of ABC superfamily members identified in the *L*. *salmonis* genome. (DOC)

S2 File Predicted protein sequences of ABC superfamily members identified in the *L*. *salmonis* reference transcriptome. (DOC)

S1 Table Samples of *L*. *salmonis* stages used to prepare a multi-stage mRNA library. (DOC)

S2 Table Hidden Markov models used to identify ABC superfamily members. (DOC)

S3 Table Illumina read coverage of ABC transcripts in a multi-stage *L*. *salmonis* RNA pool. (XLSX)

S4 Table Accession numbers of sequences of ABC superfamily members in metazoan genomes. (DOC)
