# Supplementary figures and images for: A Survey of the ATP-Binding Cassette (ABC) Gene Superfamily in the Salmon Louse (Lepeophtheirus salmonis)
Source: PLoS One. 2015 Sep 29;10(9):e0137394. doi: 10.1371/journal.pone.0137394 (PMC4587908; doi:10.1371/journal.pone.0137394)

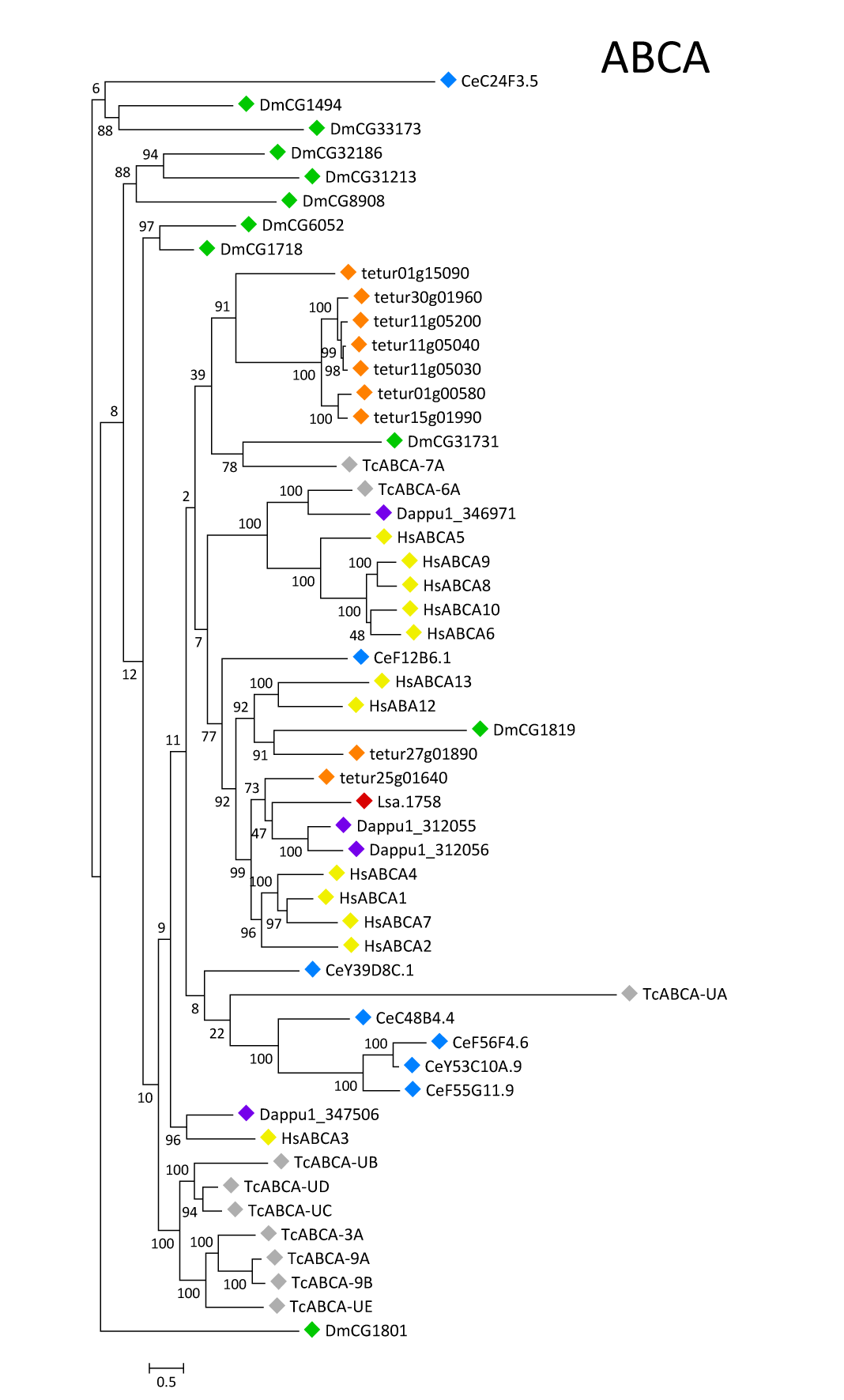

Supplement: S3 Fig — (TIF) [file pone.0137394.s003.tif]

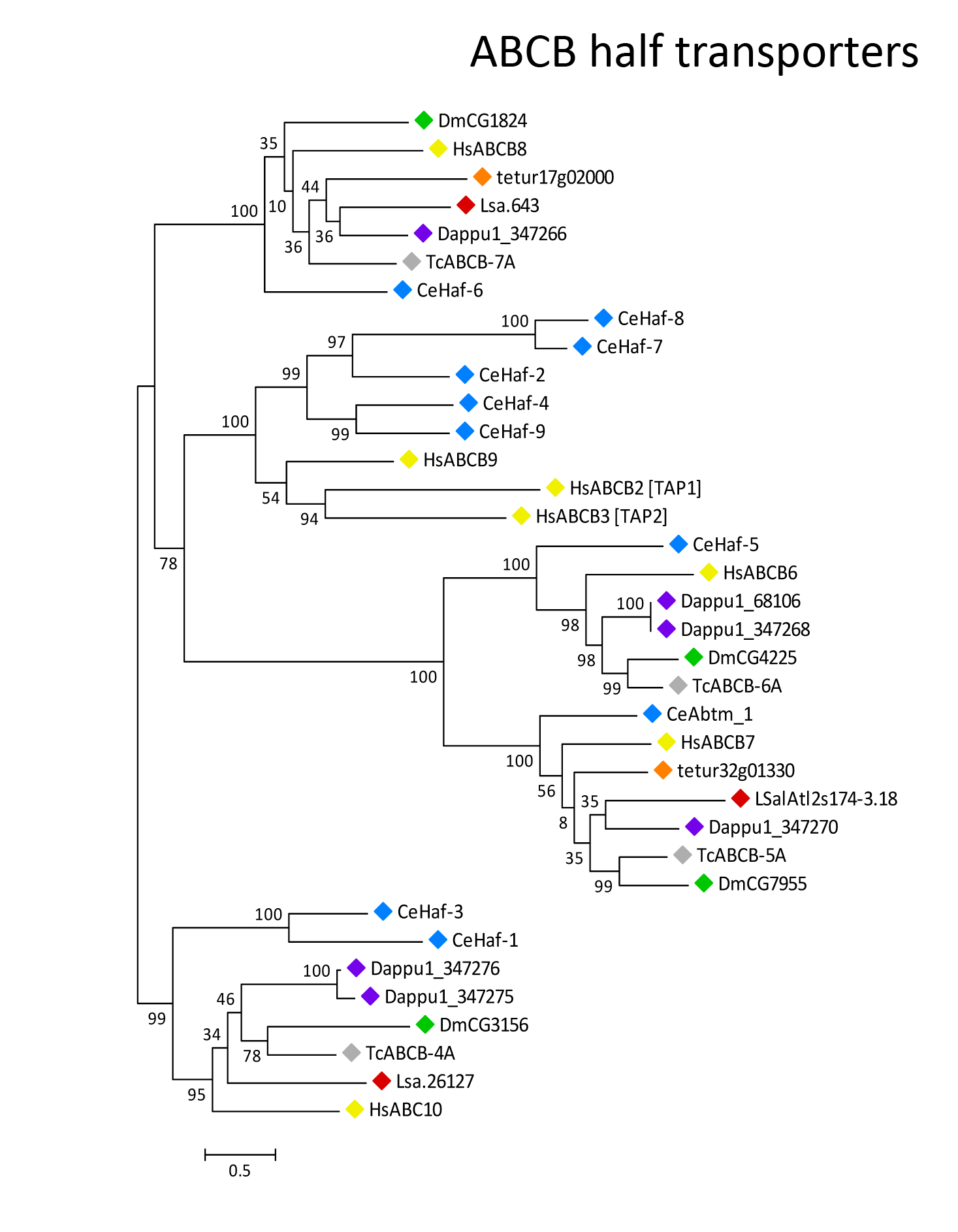

Supplement: S4 Fig — (TIF) [file pone.0137394.s004.tif]

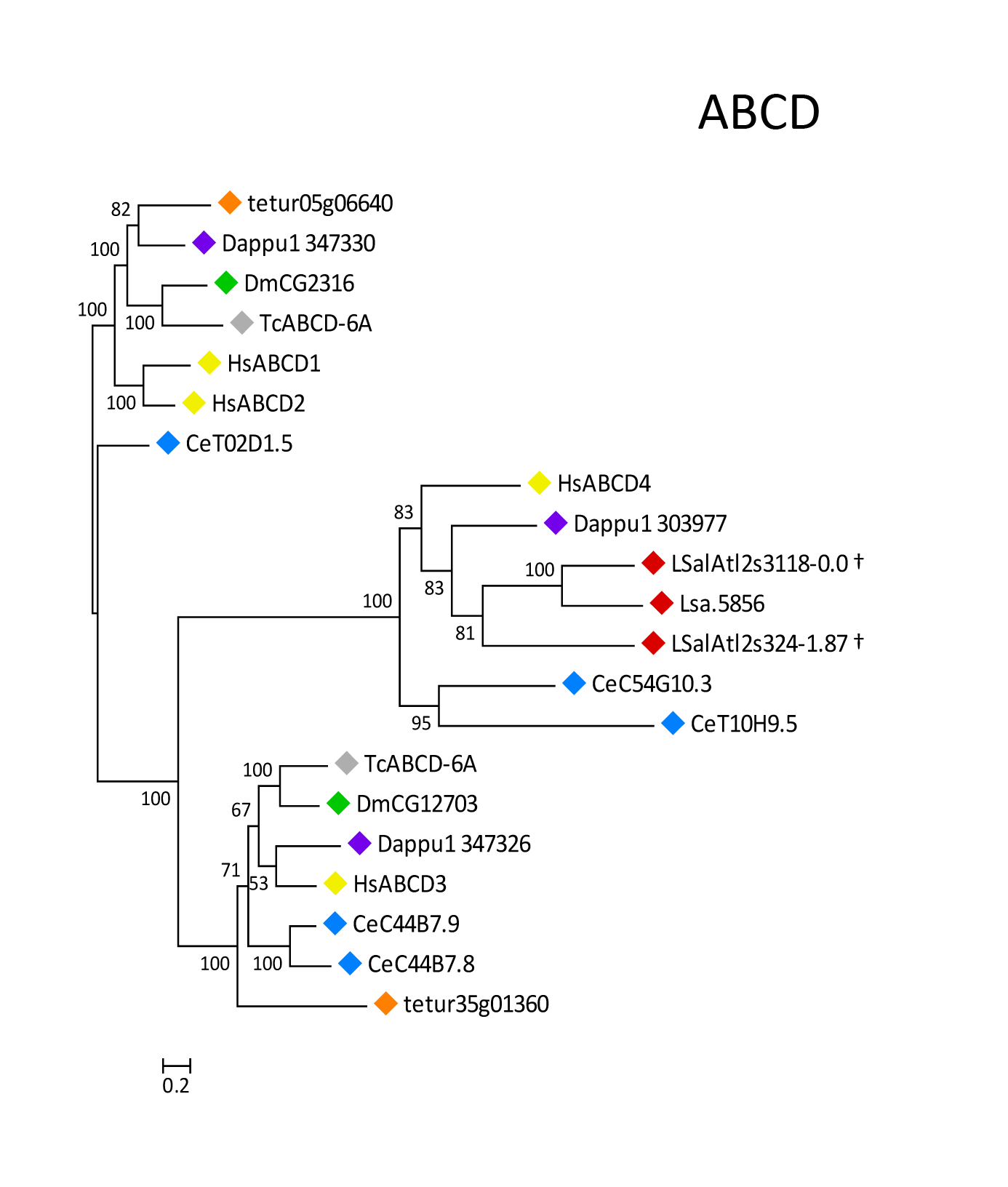

Supplement: S5 Fig — (TIF) [file pone.0137394.s005.tif]

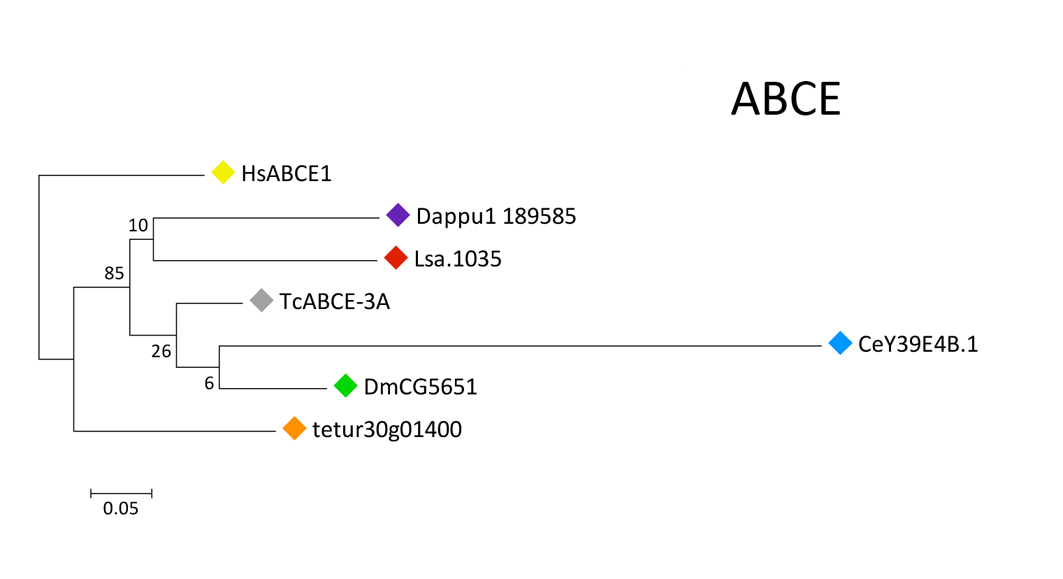

Supplement: S6 Fig — (TIF) [file pone.0137394.s006.tif]

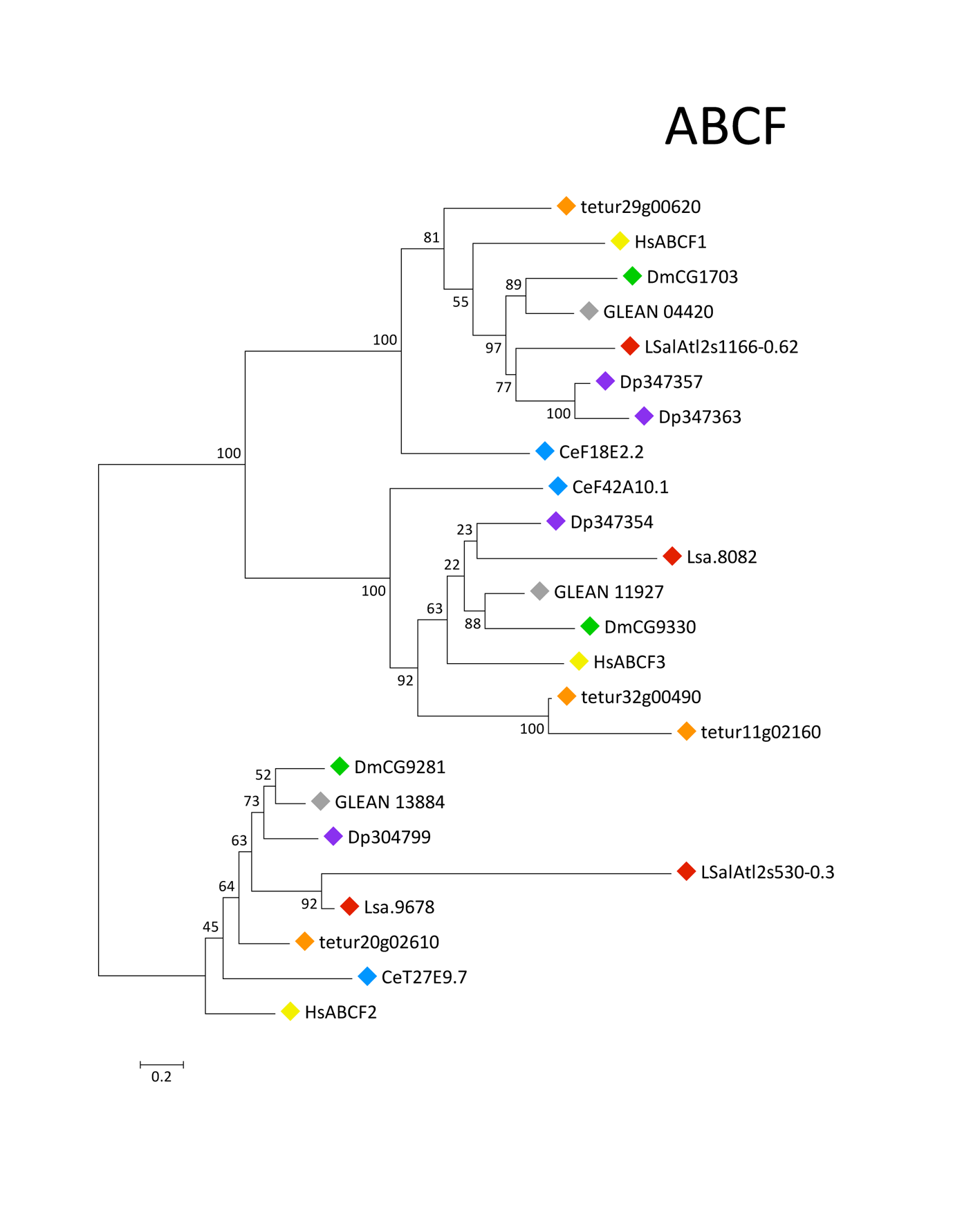

Supplement: S7 Fig — (TIF) [file pone.0137394.s007.tif]

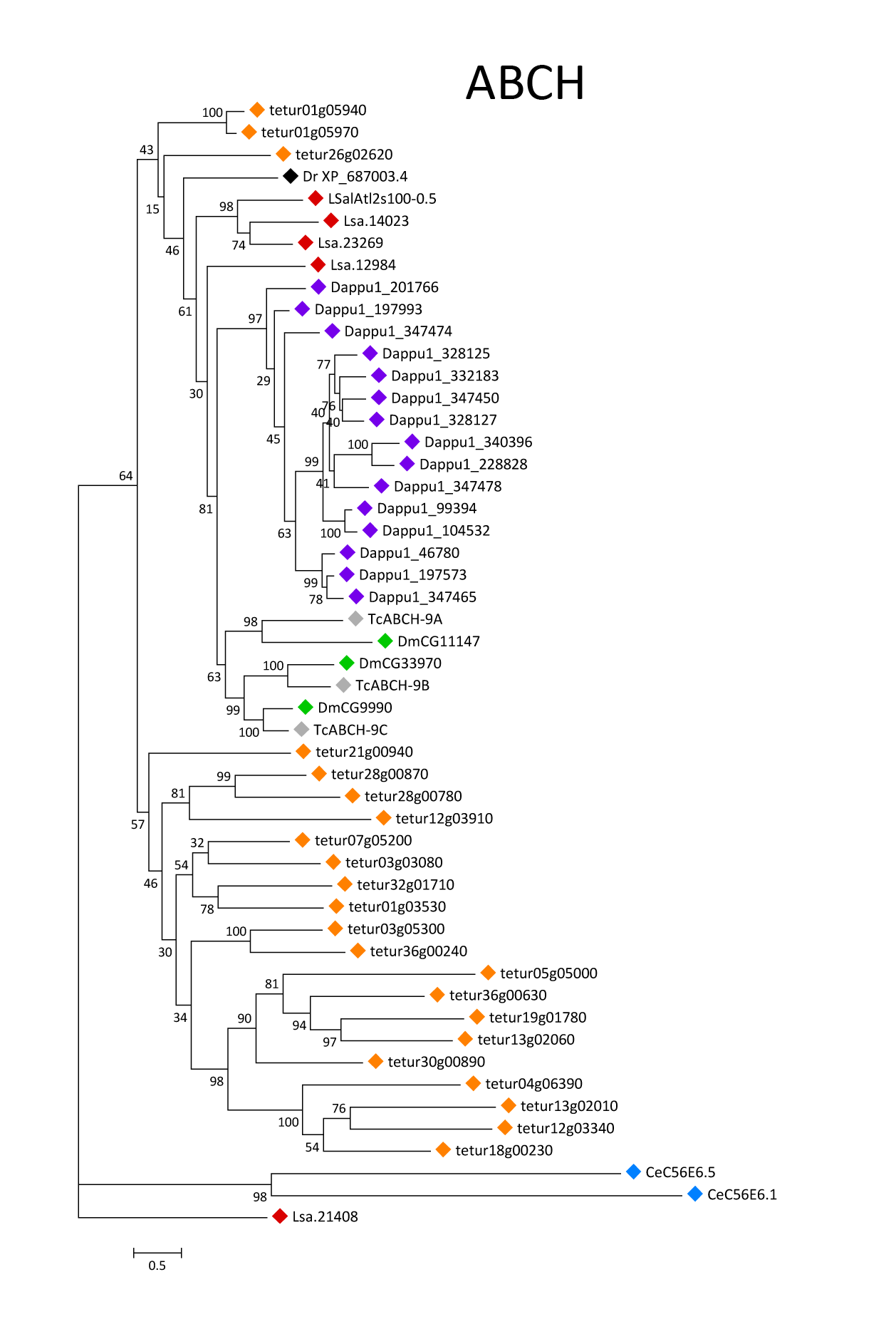

Supplement: S8 Fig — (TIF) [file pone.0137394.s008.tif]
