# Supplementary material for: A Survey of the ATP-Binding Cassette (ABC) Gene Superfamily in the Salmon Louse (Lepeophtheirus salmonis)
Source: PLoS One. 2015 Sep 29;10(9):e0137394. doi: 10.1371/journal.pone.0137394 (PMC4587908; doi:10.1371/journal.pone.0137394)
Supplement: S1 File — (DOC) [file pone.0137394.s009.doc]

>maker-LSalAtl2s118-snap-gene-1.28-mRNA-1

MYEACDKPPPNYYPPEVKLPSGDILQNLNGRNISDYLIKTRFEFEGKRFGGFEFGILNPLAGRNFDQWADSFQKISKATNMNESRVDAFTSTFAKEALVDMAYSSSTFDYIRVWFNNKGWASSVSYMNAVNNMVLRATIEEKSERMHELVDSSKYGIAAINHPMNYTSDQFSNHQLNGIVEALLNSVNILLVLSILPASYVLNYVLENNLKIKHLHFVYGVKITTYWITGYIFDTIIFCINLVFITVALIAIDASSLISDANFYGFATLIILYSFFFKKQSSALFALLNLNLMIGIIPFLTTLLVKYVNEDEKLYYVLETLFMIFPQFCLIHGLFQMFIENIKSITYEDLGFTSTASTFDWNYLGQNYLFLFFEAVIFFIANIMMEVRIFESWRLKILPDAFTKAQKETDDEVMDEDVYEEYQRVMNAYNELSKRYWRQKNSAVDKLSFGVRRGECFGLLGINGAGKTTTFKMLTGDINPTSGDAFINGYSIFNEMGRCRQSLGYCPQEDALHPLLTGREHLELYSRLRGVNKKSEIKIVNYFLKELDVIRGGQSVVLTSHSMEECEVLCSRLGIMVNGKFKCLGTAQHLKSRFGSGYSLTIRSGTTDGNLDSLKDFVLNAFPFSEIKEEHYNQLTYQIPLKTIKLSTIFHEMERAKC

>maker-LSalAtl2s118-snap-gene-1.27-mRNA-1

MNSEKEPLSHGFNVSRLSVLLNDEEEFIGSMNTWMKIKNISVKENVLQSLLDASIDVSNLSGSDILSIIGNPVSVLCKPSFDELMNVHGQINPFNITDWSLFIKTVQELQNNLLNQSSFKSVMGCFETDKFVGVSSEYELEKMGLNLIGNNNLWAGLVFQDFPPDQKDDTLPEFITYKIRMNSVTVDNTRQIQDRFPSLGPRKNPTVDLKYLTFGFAFLQDMTEHAIISIHSGRESSELPGISLQQMPYPCYVKNNVIDNIGELLRTMIFLSWIMPVSGLIKSIIYEKEARLKETMKAMGMGSAAYWMSFFLDRFSGGIIFIVTSFPLSLMKNVKVEFNTKMVACLLSNVAFGTGLDYINAYEKTGFGIQWANFMKTPFDSDNFSLGLSIGFLWLDAFIYLLLAWYIGNVFPGEFGIPKPFHFPFSISYWRGTKPALVETNSKENLDDSSQNKKDIEEEPKHLPCGISVQNLHKVYPKGLFPPSAGTIKVYGRDIQKETESVRRYLVGGSRVIILDEPTAGVDPFSRRSIWDILIRYKTGRTIILTTHFMDEADLLGDRIAIISEGKLITCGXSLFLRNRFGNGXYLTIXXYIPEDNHHEVFSSIEDEEEDKELNDCSLPNGMLIDDEGISDVSKVNGVIINPSSSNPRARTIALTKFIQKHIPDARLFEQLGSEILYLLPTEDYENSIKKFF

>maker-LSalAtl2s725-augustus-gene-0.4-mRNA-1

MPSMGPELFIKSFFCGFKNTCNESPPRDSSKMSAYNVTFVNRLLSDLEDSLYKSFNEERAAAFSKIINDISSIRELSNKAKKRFAQGLSMQGKQMNSIW

>maker-LSalAtl2s2344-augustus-gene-0.12-mRNA-1

PYAIGKIIDIIYELGDRNNSDEEKKKASTRSRLNSLCTALVVVFGIGAICNFGRVYLIQLSGQRITARIRSRLFSSITKQETAFFDTNKTGELVNRLSSDSMLVSQALTSQISSGMRSSIMALAGGGMMLFMSPQLALVGLSVVPPVAGWAVWMGKKVKNISHEYQNTLADATHLAQERIANIRTVRAFGKEVQESMAYDEKMSLVLDKGVKEALIQAKFYGMTGXTGNLIILSVMFYGGFLVTQDVITVGNLTSFILYSGYVGIGLNGVSSFYAEIMKALGAATRIWEIMDRTTHMPLDTGLILPMPLNGHITFEKVGFSYPSRPDHSIFDGLNLNIESKQILAIVGSKSTLTSLLLRLYDPNMGRVCIDGTDIRELNTTWLRNQIGIVMQEPVLFSGTIKENILYGTEGKIEHEEIVSAAKESNAHDFIMNFPDRYDTLVGERGVLLSGGQKQRVAIAXAILKNPQXLILDEAT

>maker-LSalAtl2s174-augustus-gene-3.18-mRNA-1

MLFRLCGSSMARMSSRVLLKPKKGPLALWSKNCFTPGVSGLARTGIKVVPKDKVGPKEIFSTMFHHIWPKNEPQVRKRVMFALGLLVTAKLLNVSVPYFFKKAVDVLNSEANDYFSLGTPAETVFAMASAILIGQTGALSKVIDRGSRGISFALNAMVFNIFPTIFELGLVSGVLGYNFGANYAFTALGAVGMYSVFTLGITSWRTQFRVNMNKAENEAGNKAIDSLINYETVKYFNNEAYECQQYDKSLLKYETASLKTTESLSLLNFGQNAIFSVALSAIMMMAAKDISNGALTVGDLIMVNGLLFQLSLPLNFLGSVYREIRQSLIDMQVMFQLMATPSKITSSKGATEINFINNMEDASITFDDVKFGYNIDKNIANGLSFSVESGKTIAIVGESGSGKSTLVRLLYRFYEPQLGSIKIGNYNINEVTLDSLRRQISIVPQDCVLFNDTIFHNIKYGNLNCSDEDVYKVAKLAEIDNAIRSWPQGYLTQVGERGLKLSGGEKQRVAIARAALKDSPIIIFDEATSSLDSITETMIMKALKRVTSGKTAIIIAHRLSTVVHADEIFVLSNGKVIERGTHEELLNTLTLPNGRSRRFVDLISHKDNILMDSARKDAFFTYLTIIRNKIGGAMEIKESEVLDIFTKILINSGFMLNDTLLNIGTSLSLEFSAIDHSCRPNAIYMFTGRQIVVKALCDIANFDDVRIAYIGNIEPRSIRQKMLSHQYFFDCDCEECTDDPLNLEKIKSHSPCCPQCKNLLDDDKCINCNKEVNLSRYFKIKEILKNEKEMNAKTCLYFVEVLKYFHPFDYVSYKFCDDLIARNDLIDNNKLQILYERNILTMRKYGCIYDIQMSEYLMNLIGILLSKQELKSVPNLLFEAKAIMEGIDESKSSCSKGSLESRLQSLKEDTSKFHESYWTTHNDAFIKERTQFIARLLTEKYPDQIEDKKTLSSKEMSLFYKSFLDKRKKLHWDYNLDWQKRNARILLLSMGVELKNIFNHFTPKAK

>maker-LSalAtl2s445-augustus-gene-0.18-mRNA-1

MTLALFRAATQCCFRISRSSEGLKSFTRKTLPIILGGSTTLCLVGFNLRNVKAEAPSKREKEILPSRVVEAKSEKLSSTDPSNDHPFPWIKFLGYITPHFHYLICAVCSAIAVAYFNIKIPLLLGDIVNVVSSYISETVLSEMRHDNFLREMKEPTLSMIKFYAAQSICTIAYIYSLACLGERMAASLRKDLFNSIICQDIAFFDEHKTGEIVSRLSADVQEFKSSFKLVISQGLRSSAQASGCIVSMYMISPQMTSAMGVIVPTVILGGTYIGSYLRVLSKKAQAQVAKATAVGEECISNIRTVRGFAMEDAEMELYSREVDKTRDFNEALGLGIGVFQGASNFFLNSIVLGTITYGGYLMSDDNLNPGQLMSFLVSVQTIQRSITQVSLLFGHLVKGMASGSRIFEYIEKVPLIPISGGVKIPYHSFFGDVEFKNITFSYPTRPEQAVLKDFSLRIPPSKTVALVGTSGGGKTTIAALLERFYDINGGGSLEIDGINIRDLDPSWLRGSAIGYINQEPVLFATSVIENIRYGRPNATDNEVYEAAKAAHVDDFVRTFPDGYSTILGERGVTVSGGQKQRIAIARALLKNPPILILDEATSALDAESERIVQEALDKLSKGRTSLVIAHRLSTIKNADVIAVIDKGVMAEIGTHAALKRKGGIYSRLIEQQEFRE

>snap_masked-LSalAtl2s662-processed-gene-1.13-mRNA-1

MKLGTLLQRSLSMDLNKLQEGIGEKIGLFIFFITIFISSLITAFIHGWELTLVILSAMPILMIAVGIIAKSQTALTVKESNAYSKAGSVAEEAFSSIKTVMSFQGQNTEIQRYKENLSEAQKTGILRGLLTGIGGGLMWFIIYSSYAIAFWYGVKLILDDRESCIASPTDCQIRYGPSNLLIVFFSVLMGAMNIGQASPYVEAFAIARGAASSVFQIIQSTPAIKSDYDHLQRQQDKAPPFTGRITFKNVHFEYPSRPTVKVLSGLSFEASPGKTLALVGPSGCGKSTVIQLIQRFYDPSFGVVSIDGEDITTLDPHWLRSHIGIVGQEPVLFEYSIKENITMGLQGEISEKMIDDTCKAANAYDFIQRLPKKYDTIVGEKGALLSGGQKQRIAIARALIRNPSILLLDEASSALDSQSEFIVQSALDKARKGRTTIIVAHRLSTIRSADAILVMKDGYRVDYGTHESLKSNKTGLYCSLVNAQDCQVDQDEGLPLFNPELNYEEEDEVYDLEQVENEMNSMTYGSISGGSSWNRRHHFVRPTLERRHSTGSGYSEDSLKIEDALDVAGSAIGIARVGSRKIRRTSTNFTDNEYLEAEEMKSVNSNVGFFTVLRENSKEWLYIFMGCIASVVMGASMPVYAHLFGEVLGVLSKSIEEARVNSITYSMYFLLVGIIVGFSMFMQIFMFSLSGELLTTKLRIKAFTAMLNQEVGWYDESVNSTGALCSRLSADASAVQGVINYLSFFIHLLYILIGNRKSFGHNNSSNTHNTHVYICCIIF

>maker-LSalAtl2s498-snap-gene-2.25-mRNA-1

MTTLEVGKVDVRELNLESTVMGTSTFCSDPLWNSNLTWYTEDPYFTDCFISTVLVYVPSGILFLLTPYEIWTCFNLGENSSRISRTWLNFSRTILVGFLILLSLVEFIFELLREDNVLSNIVAPGIYFLTFLNVFILQILGRIKGRVSSGPLFVFWLLEVFAAGFSFRNWRNIHRVPNTDLVTLSTIVIQYPVVVILLFMGFWADGNNNYNKIGDNDLNPSSLRMASFTSKLTFSWFDPFIYKAWKKXVTDDDLYEINDEFKSCGVLPSWDREMEKEIRRKHLKNKPINILWPLVRSFKSTLLASSALQFFYSVFQFGAPQLVDLIINFVSDPKEPIWKGYLYLFAICSITFINTLIYSQSNYYSYITGLKIRTSLTSSIFNKAVNLKSSSKKQMSVGETTNLMAIDSQRLMDFCLYIDMIWSSPLKIIIAMYLLWQVLGPASLAGLAVMILLIPINIVVGKKVKKFTAIQMQNKDKRIKLMDDILNGIKVLKLYAWEPSFIDAVTNFRVQEIGALKKSALINAVTSFMWTSAPFLVALASFTTYVLIDEKNILTPSTAFVSLTLFNLLRLPLNLLPMMIMKAIQSKVSLDRINLFLNKEDLDLLAISHESQGSHAVKMCNVSLSWETDEGLNTLEDIDLDIEKGSLVAIVGQVGSGKSSLLSGILGEMEIVRGSINVDGQTIYAPQQPWLQNETLRNNILFGKRFNKKLYRRVIDACALSPDLDMLPAGDLTEIGERGINLSGGQKARVSLARCTXNNGDIYILDDPLSAVDVHVGRHLFENVISSQTGLLKNKTRIFVTHGAIFLPQTDKIIVMKNGVISESGTYRDLIEKENGEFAQFLINYLVDEKENILKNDQDLKTIIKDLESTVGGSEELEKRLEAAQSDKSTALTDLIQGTKYLDENTSDQCSSIASRKGEDDLDEVVSNDLNERLIEEEKIESGGIKLNIYLNYFKRTGLLASFFGILFYFSYQGFSLGANLWLSKWSTDRAAINSTSIRNHYLEIYAILGFFQSIFTMLGSVTIAIGTLNASMKIHGKLLENVLRAPLSFFDTNPLGRILNRFSKDIDVAXTTLPFNIRMMIAQSFNVLGTIVIICIALPWFFVIILPAVICYALIQKFYISCARQVKRIESISRSPIYSHFAETLTGIPTIRAFGMVHHFIDENVNKIDFNAKCYFPTVISSRWLAVRLETLGNILVIFVALFSITSRGTTDPGMVGLSLSYALSVTTILNMLITVSTDVETNMVSVERIKEYENIPQEAPYDLPNSDPPPNWPEHGVIKFDNYKTRYRKGLDLVLKGINCTIQKGEKIGIVGRTGAGKSSLTLALFRIIEPSEGSIYIDGENIRFLGLGKLRSRITIIPQDPILFSGSLRMNLDPFEAFADRDIWIALEYSHLKSFVWNLGDGLNFSVLEGGRNLSVGQRQLICLARAILRKTQILVLDEATAAIDLETDDLIQSTIRSEFKDSTVLTIAHRINTIMDSNRIMVLDAGTIAEFDDPQNLLANPDSMFYSLVNDSNGMGNKNFRS

>maker-LSalAtl2s1014-snap-gene-0.6-mRNA-1

MRIPFRLLPSCISALSQGIVSIKRIDSYLNREEVDSVKSNDFSKSPRPEDGYSIQVQDCNYSIDKLKLLKNINFSTRVGELTAVVGKVGSGKSCLLAALCGELNQRGKGSSLINKDLVYVTQNIWLKSATIRDNITFGQPYNSSQYQKVVSLCQLGQDFKDMYRGDLTYLASNGSTLSGGQRQRIGFARAIYQNAQVYLMDDPLSACDSNLKAQIFHNTIGPKGFLQKKTRLLITNQSSLIPLMDRIIVIQNGTVEEKDSAEEVKSFEIAKKSTKKQGSFHEPPKDNYLHPALDNYDIIERRKIPFSLYKYYVLNLGTVPFIIALVGYLISQCFDVCSKLWLSRWTNLSSTNINDTNYRHIYNEDTRNMYIVVYGILGWCQSLAYFLSVLLINSRSLKASSHFHDVILRKVFGAPLNFFWSTPKGTIINRFSKDMDEADMFLPNTLKNFAYQAVKILGTLIIALIAFPITAFFIVCPLGLIFIEVVKSYLMASRFLKRTSATKLAMVLKHFGDSAVHGGTTIRCYGVESDYIDEHMSLIDEHQSVSLMEIISEAXLFLRLQLIAGTFIGILATALVFFPSEGNSSSLSALSLTSSLTVLQDIFLFTRYAAFIEKAMVSIERIKECEDLIPQDPSSGLLPSIITNGSKRPKNHQSRYYIQFEDFSGKYSSLKNASIKNFTLNVRLGEKVGVVGRTGSGKSSLILALSGLLEVEKGKIFIDGVDMSSGTARNIRKKDITDAALFKGSIRYNLDPDDRFTDEYIWSVLDEAQMKEFFVQLPGGLDFSLKENGSNISLAGSNLIIMDEAXSAMTLESEERILNTYFSRFSSSTIFVIAHRIQPLLACDKILVLENGTIAEFGNPESLIKSQKSIFKSMLRRTNGSVE

>maker-LSalAtl2s1014-snap-gene-0.5-mRNA-1

MKLLSSEILISGLYEVFHILFTLTNPIALKMLMDYIEKERGDYLRGIYSILFLTVTGFLSSLCETHTFYHLNLSGFIMKTALMRGNVISLVSVDCQWLVKAIRFIHLPWSCPLQIIIAIYLLYNILGVAIVPGISFLLSLLLQKFQKSHLKKKDIRLSRIMEALGHFKTIKLNCWEESIIDKIGILRSDEISVMKKFLSMQALQPLPHLVFTRIK

>maker-LSalAtl2s812-augustus-gene-0.6-mRNA-1

MNCMSFRNLSHILITVFVSTLISFPNPVISENSILNSESLYRVFKIERELFKVLQERQKQLQDSLELIKEYTRDVESLYRNEGCWPLESCIESKIFEQIVGNPIYNYQVLRRLIVNWKNTEDSLKKIDIKQIMTDIKRLKKQNGGLPDGKDLQTAAKALNRIHKIYDINTTEFSNGNILGHQTSANLGLRDTLYLGRFAANTDYPGVALNWLNTAQSQANLHPNSSEEALKQVKLALRQVNRKLSKKPKSMGDKDPYILGAIPDKTEDPNLVISNTDRRNFEALCRGEKLISSFEESKLYCYYDNRGVFGLKLSPLKVEINHREPHLILTFHDLFTQSETHDLIESVRSDLTSAGVGTDKTISDMRISSHVWIPDGMLSIVDKISEKIENVIGLRSMAQLDPEAKKEEYEMLQVANYGLGGHYDCHHDSMFIYKEPQFIPKSVEETQSPYITGDRMSTFMIYLSDVQKGGNTAFPRLGVATSPRKGSAVFWHNIKRSGRSDMHMIHGACPVVMGSKWVANKWIREFIVKRFVEIHTQKMNESWICEEPLWNQVSDSWKTLDHPNVSSCFRIFFFRLIFPNLCFFYVWLPFEVHPIYSSRDKGIKRSLLGTLKDLFAFFCVSVAIYDVIHEAFFKPNFQILEILDPSLRILTYTLVLVLNFLYRVKGMRISGFLCRFFFVSLFSFGIDLYLMYEVMTQLKLEELMSSIIHVTLLLLNVLAHFWPEPPPRYSEYVRLVGVEGCHNPSPDTQCGYPSYIFFSWMSSLMWKGFKRPIKQKDLWDFIPSVNTALVNPHFLKHWNKIYTQTKFSNYPTSKSPSIFPALLRSYWPLLLKGAFAKLLHDLLLICSPMLLQYLILFSYSPDIPLGQGIALSVILFGNKILGTFFIARYFFYMVTVGMKLKTSITSLIYRKALRIHSTRDDTTSGEIVNLMSVDVQKIVDLMPLLNTIWSGPLQIIAAIYLLINTLGYSALAGVLVMFLLIPINGFIAVRMKNAQMKQMKKKDERVKKMNEILQGIKIIKLYAWESSFSKLISSIRFEEIKLLKLSSKYFGFMMLTISSTTFFISLFTFIVYVVSDPENHILDSEKIFVSISLFNILRFPLNMLTHVIGGIASASVSLKRINKFLASEELEEGILVRSNSHDHQAICIHPSSSFSWKGSSESLFKDISMDVNRGNLIAIIGPVGSGKSSLISALLGEMLFTSKGTNPVEIHGSVAYTPQNAWMQNVSVKDNILFGRKFNKNWYDEVLKACSLESDMEXFPSGDSTEIGEKGINLSGGQKQRISLARAVYSQSDIYFLDDPLAXVDSHVAKHLFEEVIGSNGLLRGKTRILVTHNLSFLHLVDEIYFLKDGEITEKGTYKELMEKNCDFSELISNHANNKITNQSDSEKVMEKINEEGTKQPIETNFKIYDEETLHTGQVGIRVYWYFLSKIGMLAGILAFSFFIISQILATGSSVWLSVWSDAKNNTDIYYHLGIYAGLGVSNIFISTAGTLILSLSLLDASRILHGTLFQCIIKTPMSFFDTTPLGRILNRFGQDIEVLDTKMSFCIFGSMTGTFAYIATVTIISINVPIFIIPTLAIFVVYFVILIVSLSSSRQLKRLSSVATSPIYSHFGETLSGINTVRAYSLEKAFYSEFERRLDEYQKRNFPVIMADRWLGIRINLIGNFIIFASALFSVLSRDTITPGQLGLNVSSSLAITSILSWVIRMYSDLENNIVAVERIKEYSNKESEAPWELEEDLKLNSSWPLKGEITIKNLSLKYRKDLDWVLRDINVHISPGDKIGIVGRTGSGKSTLALALFRLLEVNSGQITIDGVDIKKMGLHTLRSKLTIIPQDPTLFSGTLRLNLDPFGLFKDMELIESLKHAYLQDFLKEDGLDKVIAENGENISLGQRQLICLARALLRKNKVLIMDEATAAVDSETDKLIQKAIKDNFRDSTVLTIAHRLQTVLDYSKIIVIDSGCIIEYDSPKNLLSDSSSTFYKMCLNSGINQ

>maker-LSalAtl2s1420-augustus-gene-0.12-mRNA-1

MEQLNQDEEANSTTMYLNGFCSDRDPFWDPDYAWNTNDPNFTSCFRKTILVWIPCLFFWAFLPFHIYKLYHSKARKVPLKTLGRVKALSCFILILIAFVDLGYWGSKEFYPKDLINPIIRAITFLAVFILLFVERARGYRISPLLTSFFLIYFATNTIDLYGHIRWVMMSGTTXLADITFFLHFFCIIVSFLCHFFVEPRPLYEDTAVNDIKSNNPCPLITSSFPSQISFSWLDSLLWTGFKRNLSFDDLWDLVPSLSSRTVVPIFLQRLDSALRRVKTNENGITYAKGDDNVEVKTKHEKKPQLSILPALARTFGPSILSAAVIKIVSDSLNFVPPLILKRIIKFSTNQEELWKGILYAVILFASSTVGSLVLSKFFYKMYVVGMKIKTSLISTIYRKALRVPTSTKKNISTGEIVNLMSVDAQKIVDLMPYINTVWSAPFQITIAIYLLWQTLGPSVLAGVLVMILLIPFNGFIASRTKTLQTNQMKEKDERIKLLNEVVQGIKIVKLYAWEQSFLDIISNVRSKEVKILTHIGYLQAGNSFIWTCAPFLVSLVTFATFILSSPENVLDSEKAFVCLTLFNILRFPLSMLPMIIGSMVLAGVSIKRINKFMNSEEIDEEAVEKKTAYPDDKFAINLEKACLKWESDEDKNILSDVSLDIEVGSLVAVVGTVGSGKSSLLSAILGEMDKVSGLITVRGSIAYGAQQAWVLNTSLKNNILFNKSYDGDKYTRIVEACALKSDLDMLPGGDETEIGEKGINLSGGQKQRVSLARAVYSGSDIYLLDDPLSAVDSHVGKHIYDNVISSSSGILKDKTRVMVTHGVTYLPFTDKIIVMKDGRVSEIGTYKELLRQKGAFAEFIVQFLSEANENEVNENIKHDIEESFGKNELLEQITKAKQIVRERSVSLNSGDLVTSLVKNNPTNSASGSTTSLKENSGDESTDNDTKQTRPTTSQTQQYQDEKVETGSVKWNIYMHYVKNMSIILVVACSSCFVIYQALNTLSNVLLAWWSDAVMKIEVRTGLNDNRTLDEINQDIMDXQVYYLSIYGGYCLGQGIVVVMGFVFMYLACMEAAQRLHNQMLESILKSPMSFFDTTPQGRILNRLGKDLDVLDSVMPMVLRGWISCFLGVLSSLIVVMVTTPVFIIPASLIIICYFFIQRIYVATSRQLKRLESSSRSPIXSFFSETVSGAATIRAYGQSSTFISESESKVDDNQKANFPATVSNRWLAVRLEVVGNLIVFTAALLAVLGRDSLSPGLVGLSVSYALAVTASLNWLVRMASEVETNIVAVERIQEYTKAEKEAPWIIEDTKPDEDWPRKGEISFKNYSTRYREGLDYVLKNVSLDIEGGEKIGIVGRTGAGKSSFTLSMFRIIEPVTGNIIIDGVDITKLGLHQLRSRITIIPQDPVLFSGSLRRNLDPLDEYNETNIMEAISHSHLKPFIDSLKDGLEYHVSEGGENLSLGQRQLICLARALLRRTRVLILDEATAAVDLETDNLIQKTIRSEFKDSTVITIAHRLNTIMDYSKILVLKNGERVEYGTVAELLADKKSQFYSMCSDAGLV

>maker-LSalAtl2s111-snap-gene-2.14-mRNA-1

MDIEEKVNLKDNKRSNANILSKIFFIWVLPLLRDGQNKAFDIPDLPSALTEDKSRYLSDNLEREWKKELEKGLRYDNTSKKRYSPSLLRALIRTFGSSLGVYGAFSFIEECVFRLLQPLAISQIVLYFSNSNHGISPTQLYIWSAVLIMSGVLYVFSHHWYFFGVVQVGMRIRIACSALLYKKSLKLSKASIGKSSVGQMVNLLSNDVNRYDLCVLFIHYLWVAPLQFILVSIITWYMVGISSMCGGAILLVFIPLQTWIGKQFSRLRILIAGKTDKRIRVMNEIIEGMKVIKMYAWEYPFMEVVNETRRDEIQTIKKTYEYKAFNLGFFFTSSRVVLLLIFFLMIIGNEVISSKNIFLIFGLFNTVRLSLTLFFPNTISMTSEALVSTDRIQNFLLLEEIGDICSSMKHEPEVRPEVSLRIEMKNVSGKWTSNEKDDDLRNVSFQVHKRELTAIIGPVGSGKSTILQALLGEFPVSSGDISIYGKISYASQEPWIFSGTIRQNILLGASMNHKRYLKVLKVCSLEHDLESWPDRDHTFVGEKGVALSGGQKARINLARSVYSEADVYLLDDPLSAVDSHVGRHLYEECIKNYLSRKTVILVTHQIQYLGDASNIILLNTKGEIEDQGTLNKLLMSERDFTSFLVAQEEETDSIFDEDELALTPKKSLNIENYMRRRQSSVSSIGSRATIDTNAMEYNDIPNEGRRTQTTESKVKGSISVALYKKYFYAGGGKWIFFFVYGLNILSQLLFVSTDWWLKQWTNAADARTNNYANASSFEILPGFQIDLFNSIYYGIYFALVGALIICSQISIRKFLILCLKSSKNLHLEMFQKVIFTKPAFFDVNPVGRILNRFSKDIGSLDXLLPLALSDTSLIFLNAVGMFGLIISTEPKVLIPLGLIQRWVFPLLRDGQNKEFDTPDLQSPLSEDKSRYLSDSLEREWKKELEKGLIYDNTSKKRYSPSLLRALIRTFGSSLGVYGPFVFMENCVFRILQPIAISQIVLYFSNSNHGISPTQLYIWSAVLIMSGVLYVFSHHWHFFGMVQSLKLSKASIGKSSVGQMVNLLSNDVNRYDLCVLFLHYLWVAPLQFILVSMIAWYMVGISSMCGGAILLVFIPLQIWIGKQLSRLRILIAGKTDKRIRVMNEIIEGISLTLFFPNTISMTSEALVSTDRIQNFLLLEEIGDICSSMKHEPEVRPEESLRIEMKNVSGKWTSNEKDDDLRNVSFQVHKRELTAIIGPVGSGKSTILQALLGEFPVSSGDISIYGKISYASQEPWIFSGTIRQNILLGAPMNHKRYLKVLKVCSLDHDLESWPDRDHTFVGEKGVALSGGQKARINLARSVYSEGDVYLLDDPLSAVDSHVGRYLYEECIKKYLSRKTVILVTHQIQYLREANNIILLNTKGETEAQGTLNKLLMSERDFKPFLVAQEEETDSIFHKNELALTPRKSLNIENYMRRRQSSVSSIGSRATIDTNVMEYNDIPNEGRRTQTTESKVKGSISVALYKKYFYADGGKWMFFFVYGLNISSQLIFVMTDWWXKQWTNAADARKKFGDIRNQKHFFSSYANTSSFEILPGFQIDLFNSIYYGIYFALVGALIICSQISVRKFIFLCLKSSRNLHSEMFQKVIFTKPAFFDVNPVGRILNRFSKDIGALDDLLPIALSDTNLIFLNAVGMSGLIISTEPKILIPLLAILIILLALRKYYLNASRSVKRLEGITKSPVISQLSTTLNGISTIRASKLENNFASEFHYLQDIHTAAFFSFQSVTRFFGFWVDGIVSINVXATVVIFVFFNVKGGDIGISLSLSVLMARXIQWGLRQSAEVENYMTSVERVAEYGDLPSEKRLTSDKKIDPSWPNKGVIKFHNVKLKYDDQGGPYILKGLTFTINSFEKITLIKFHL

>maker-LSalAtl2s111-augustus-gene-1.10-mRNA-1

MIQWGLRQSAEVENFMTSVERVVEYGELPKEKGLESDIKLDPSWPDKGVVEFSNVSMRYSQHQPNVLKGLNFKTNSFEKIGIIGRTGAGKSSIISALFRLAEPEGEIFIDGLDICKVGLLDIRKKISIIPQDPILFNGSIRKNLDPFNEFTDVQIWNALEQAKLYDIVVDLGHGLDSVVLEFGSNFSVGQRQLFCLAXAILRRNKVLIMDEATANVDPFTDSLIQEAIRKEFKDCTEFGTPKDLLKDPNNLLSRMVAQSEPNTAHTLRHLASGIIDSIEESQLEENISVNVSSKCLYTNTNKCLEILFDDLIGFQWISNRNISRRLIQSPKQISSRIFYFCKAIIFIKWTIPFIRKGQKEDFDVHDLYKTLREDYSRELNDRLEKEWQKELSKVKTFSGNYHPSFAKAAIRTFRLQTLLSGXFVLFEECVLRIAQPFAIFKIIGYFSSGGTXMSEXELYFWSGILVGAGVIFVLFDQRYWYLSLKTGMQLRIAASAIIYRKALTLSKASLGRSSIGQMVNLLSNDVNRFDNSTVFLQYLWVAPIQFICVMLITSYFAGIEAVAGGALLIFFIFFQTYMGKMFSKLRLMTASKTDIRIQIMNEILDGIKVIKMYAWETPFVQLVTNARNRFVLLPVFLLMVLFGVEINAQKIFLIFGLFDAIKLPLTHFFPSAISASSEALVSMKRIEEFLLLNELRDVQSKINHVILPGRYEPLTVEVTKISGKWLDSSNEFTLKNISFSVEIGELCAIIGPVGCGKSTIIQSLLGEFPVSYGEINIKGRISYASQEAWIFSGSVRQNILMGKPLIDKRYREVIRVCALEHDVREWPDGDYTFVGEKGISLSGGQKSRINLARCVYASADIYILDDPLSAVDPHVGQQLFDTCIQKFLSDKTVILVTHQLQFLKNANNIILLNSDGEIEASGTYEKLMSQSGFVTYLEATSEEEDSDLLSDVLSDVKSDGEYMRSQKKKLKKGVRSRRSSTSLLVPVPYIGGSTERDTFPEEKGSQEWKMGSKETIIEGSVSLSLYHKYFVAGGGFFKFLCVYFLSLITHTLFVVSDWWLRLWTNAADARKLGPLPPTFYFSEYQDMTLQITADFQVDFFNFIYCSIYLSLIIILLFSAQLSVRQLFMSCMRSSQTLHDRMFEKVAFTNSRFFDLNPNGRILNRFSKDIGSXDELLPPALLSGSGIFILISMTNPLIVVGIVVLLLILLTIRKYYLNASRAVKRLEGITRSPVFSQLASSLDGLTTIRSMKIEEMLIEEFDYLQDIHTSSYYSFLVVNRFFAFCGDLMLSLFTASMVSMFLFYNRDATGGDVGLSLTLSLALCGMIQWGLRQSAEVENFMTSVERVAEYGDLPKEKGLKGDIKLDPSWPDKGVIEFSNVNLKYDGTTPYVLKGLTEPEGKIFIDGVNICRLSLQDVRKKISIIPQDPLLFKGTIRKNLDPFDEYGELAIWHALEQAKLSVAVSELEGGLETEVSEGGNNFSVGQRQLFCLARAILRKNKVLIMDEATANVDPMTDSLIQEAIRTEFKDCTVFTIAHRLYTVMDSDRILVMSDGEIKELGAPLDLLSDPSNIFYQMVNQLGPSAAERLKKIAQGLQSP

>augustus_masked-LSalAtl2s128-processed-gene-18.4-mRNA-1

MLASSIVDSDIKREFCGRGSLHPWDSSINDFGICFQSLVLVVPTHAILGIISAYYSSYEHGSYYLRPQKAIIAIVSRIIISLGLAAHSALFLLLREKYTENPDGSSLLESFVKIICWTCHAAYNYNLLHRLSLSPRGPNRILFIWILCLIPDLIQARSNLLQPLTIPLIERNLLFYEALFQNIFLATYSLTLFFGPTESESMSYQGPGERDRLWTRAQSYGGFHEEYDYNYLGVAQEDTSIMDXXFFKWXSPLIDKGRMGKLNSSQDVFDLPHNIHTGIVYEDFEFHKSRVRSLQWFRKALSSKFLKEFLCIGMIKFVADVSGFFCPLLLNRLVKFMEDPKADLRWGYFYAFSLFVSTFLVAICNTQFNFKMNELGLKVRASVIQSLYKQTLSVSEANLNKYSRGEIINFMSIDVDRVVNFAPSFHAFWSLPFQMVVTLYLLHQQVGVSSFVGVGFAILMIPINKVIATKIGSLSGHMMSAKDDRVKIIAEIIEGIRVIKYYCWESFFTDKTNSHRNNEIYYLKWRKYLDAVCVYLWASTPVIISVSTFATYSALGNPLTAAKVFTSMALFAMLSGPFNAFPFVINGLIEANVSIKRLARFLSLPSINRIKYFTEYKDENTKASKEIETKIPDIQIMEASFGFTKDCFTLRNIDVSIKRQEFVGVLGPVGSGKTTFLNAILGELEKQSGKISVRDPLSGIAYVQQVPWIQNKSIRDNILFGEMYIHGKYTKVIKACCLDHDFKHLHRGDHTIAGEKGAALSGGQKARIALARAIYQDKDIYLIDDVFSSLDVNVGYKVYTEVMLNLLKRKTRILCTHNPQYINDANIVIKIKDGEFESVKQSTHLVPTSTSPDFSYDTSMFDFKDNSNVIDDSVEEEMQETGVVAYRIYKKYWQAIGTYLAPTILISIFLMQVASNTTDLWLSHWVSTDNFQNNTEDTDHYIYVYSGLAVFHTILTLIRAFLFAYGGIHAAKIIHDLLLGVLLRAKIYFFDSTPAGRILNRFSSDTYAIDDSLPFILNIFLSQIFGVFGRVLVCVYAVPWILIVLLPLGLFYYEIQCKYRPGSRDLKRISSVSLSPIYEHFNETVHGLKIIRASKASQRFLLENEELVECNQKARYAAFSASLWLEIRLQLIGSIVVFSIALISVINFESVDAGLVGLAVSYALGMTXRLAEVVVSFTETEKELVAVERAYDYIDRIYEESYSGVLNMPYNWPNQGVVEFKDIRLRYKDHLPYVLNKVNFKTKPKEKIGIVGRTGAGKSSLIAALFRLSSFSHGEIMVDGIRIRLLPLIDFRRQFAVIPQDPFIFSGTIRQNLDPYAQHSDQELWDSIKLSYLYNIIYSFGSSGLDTLIGDGGKSLSIGQKQLLCLARAIITSAKVVFIDEATASVDKETDRLIRNVLKTAFNDKTVITIAHRIETVLNSDRIFVMSNGQIIEEGKPEDLIKSPNSEFRKLIEQK

>augustus_masked-LSalAtl2s1361-processed-gene-0.2

MSEEEPPIDESLTLCLSQIQRFYYYSILLKEETEKKYSNCVLEFTQISTNTLFCYIVTMIQVYLMISNSSKNSARSANGSGGICFGLEGINNGPIHARYVSHHFISAIYIFQFIVALFSAVEVVCFWSKTNIFMAXSFVDSFLFIVSIIASGAYFVSVEKRHLRYSIPLFLHYGANTLIQGFCLLENSDFRYIRTWCAFLLSTSYGLQFIFGFIGIIQRLYLRLVSNMDGPVYSKRPVIPQSHVYLYNEASSFSRLTFWWLNPILTQGYRAPLEIDDLHKLPKEERTKKYFQKLKTLLSRDKSSILWECITMNWSFVILGGLFRLFADILGYACALSINIIVNSIAAENENASLHLISNVTINPLKDTRYYDSFFVSELFFDPRVVSIVIFLAALGQGALSQTSNHLLTVSGIRAKNALHVLLYEKSLKLPVGSSNPMQIHRKINLKPLNDEGKGCMGGSCSLDEDYSNEGNIDIGFITNLASEDIINIRELIWNVHYIWALPLKILVLLFLLYDQLGISGVSGAILGTCVILPLQVLTGKLMSENNKLILCSQDNRLFKSTETLASMKTVKLATMEEWALKRIRCARTKELIFLRRDSFLWSFMAFLASISTTLVTTLTLGLFVLLEDHNFTAADLFTSMALLSQLTVCLSVVPVTVPIFIKGKVSTQRLAEFLNRAEVSIYKQNNKLNKHCTYFDDRKDDDEEEEEEEEESEIFIQRAKRLPETCFTVENGTFAWPKCETNVLQSINLEIKTGSLTIVIGPSGSGKTALISSLIEEMDRITGSVKWNVPDTVALLGQRPWLLNTTIKDNILLGRPFKEKRYKKVIAACDLQTDIDLLPHGDDTEIGERGVLLSGGQRQRLAIARCLYSKSYCTFMDAPFSSLDSKITSHVFEEGVLKILLKRRRTVFLTTERLDFLHRADHIVALKDGMIKAQGTIQDVTRLCPELLSTKLSKNRLSRISDDNKGLVEGKTAQERWKLLRNVTKLGIKVKNKNTPKKKSQLQLNKSDDNFPLEISPVNSLLIRRHRSHSGGTSSGSSGGSFLFGKSLSQLPSSASSCKLYRSHLRIDSRSQSNXTHDLLLPSDEYIDYSLTEEEPSFCRVKKLSKINNGNNLFARTLSWNTNSNELSVSTSSSMNRSVVRRAKTPPNRPSNHAGEFQRMRSFIVNTNKNINLTSNRNAGGRSSLHRDSMVLSNLMPMHSNSEPEGFITDTVAPLKRRSIVLEPPHNRVMRLTSNSSQISSISGFSDDFFDEEEDEEGLVYKSQGGQSHENREYGSIDIGVLTVYFKAGGIFLAGFFVILSFVLQSVKVYMDFLLRDWSMEEKNSDKLIAYFSFYGSLSVIVILISCVANLIGQLIGARARTKLHNDMIQNIMYCPLELFEAFPIGRIINRISHDIFIVDQKIPPCIQRLIMLSFVCIAALAXNSIQSPVFLIFALPMISIYWWLXHFYRRSSRELQRLDSITRAPVLSHFSDTLSGLITVRAFGEQTRFINELCEKVDTNTSAFLILQSGCRWLGVYLDAAGAIFVFLSILVNLFFPRKGREVTSSASIGLSVNYSLLVPIYLAWVVKFAANIENYMNAVERVLEYTHFPSEEEDFSEFQAVHASSMNSSRRSFLLRGRGKHDEIEEDVRVSGDFLQNDSEGLIIRFNSVCLAPSFEYRRLPGIQQGFSLEIPYRQKVGICGRSGSGKSTLLMGIVRLSRVLQGSITINGININAIPLSRLRKFVITIPQDAVLFSGTIRSNLDPENDFSDELIWSTLDKADCGKTVRNFPDGLDTCVTENGDNFSLGQRQELNIVKALLRRPRVVILDESTSALDPNREVALHNTLLEAFEDSTLISVAHRLSNIVEYERVLVMGEGRILEDGNPKELLKKPMGFFSALWRAAGEKPLS

>augustus_masked-LSalAtl2s197-processed-gene-0.4-mRNA-1

MILFLLSPLELLYNKNKCSSPKTLFDPFSIFKAAIPCILIVLSILEGIFIQDFDTLSDFLKIFLMITSFGFSLFLHVDGVIRRCSKTSTLQFFFYFLLLISSCLSIRRIESHPEDLGRDYLWLICLQTGCVLVATVLYSISETVLKQKSKKNKENPKKDASFFSLLFYEWITPLLWKGFKKPITEDDLWNLNKDLTCGETSRRLDQYYFKSGNKKIGIPLMKAFGFEFVIGSTIKFFSDILSMVLPQIMKLMINHADSEFEMKERSWKGYLYPSMLIIVSFLQSVLLAKYFEIVFLVSMRIRASLTSLVYRKCLKLSNSSRKNKSVGEIVNIMSVDVSRIADLMPFLTLLWSSPFQIVVSVIFMYNELGWAIIGGTGILLITIPVNAILSYFGKKFQMKQMKAKDNRTKILNEVLGGIRILKLYAWEPSYINKIEGIRGVEIGILKKAAWLTSFMGFIWSSTPFIVALASFATFVMVDDRNILTAEKAFVTQSYINKMKMPMAXFPFVIVXAISATVSLKRINQLLTSDELQKDAVERVPMKINQKIDQGDAIVIRDGNFKWSTEDSENTLSNINLRIKHGSLTAIVGTVGSGKSSLISAILGEMVKKSGKVRIKGQIAYVPQESWMQNTSLKNNILFGKPYDEEWYNQVLEYCCLTYDLSLLPAGDETEIGERGINLSGGQRQRISLARAIYSDADVYIFDNPLSALDSQVGKSVFNNVLNNNTGIIKDKTRLLVTHGISYLPHVHHIAVMLKGEIIHEGTYQELLEKKIIQTIEEEEEQEKDGVQENNINNYNNIPKTFDATVNNLEKAAIIQKEGLETGRVSWSVYKYYIQSIGFLGAFLIIFNQMLNQLFGFGSSIWLEAWTQEDYGNATIPKYRDLYLGVYGALGIGQAVTIFLLSLSVAMFTLKASKVIHNQTYDRIMRAPISFFDSTPQGRILNRFSKDISICDNILGSNIRQWLXCLFXFLGIVILIVSVLPLFLLFALPTSLVFILIQNIYVASSRQLKRLQSVTRSPIYSHFSESLNGLSSIRAYGAQSKFILESINKIDTNQRCNYPSIIANRWLAMRLETIGNLCIFGXAILTMTNPELVGPGKVGLVISYALILTQNLNWLXRQTSEIETNIVAVERLKEYAGLDIEKEWRLHKDTSYNWPSQGKVEFKNYSLRYKANSDLVLKKINFTIFGGERVGIVGRTGAGKSSLSVALFRIVEAANGQILIDGKDISTLGLHDLRESLTVIPQEPLLFSGTLRSNLDPENAFSDHDIWNTLGSVHLKEYIRDLKDGLNHSISEGGLNLSSGQRQLMCLARALLRSCKILILDEATAAVDWETDEKIQETIRCYFPSCTILTIAHRLKTIIDYDKILVLDKGTVIEFGSPNELYMNSDSVFHSMINAAGITLTASPKNEIESTSL

>augustus_masked-LSalAtl2s3118-processed-gene-0.0-mRNA-1

MKNKLSSVLTGNYRINWKLFVRIGKLHAIISRNLINIFVTICLLGVCILEQYVGYKTGLVAGAFXEVLLNKDTEGFKTTAIHSFGVILGIAVVKTVRMYVAKILSVFWRRTLTTNLHNLYFDADSYYKVNVLIKELGLDNPDQRMTSDVDTFCLVYGEMIARLIISPFTIGFYTWDAYTRSGWIGPVGVFIYFILGCFVNLFLMSPVSQRVFFMEKNEGDFRFKHMFLREKAEEIAFSNSAIIESIKTNKLLKSLIKSQLSVYTWEILLDLWINIFDYVGAIISYLIIAVPVFTGFYDSLDQAKLGNQISQNSFVCMTLIYNLSQLVDLASKFANMAGVTHRMIELVEILEKHNSNPCFFKDNAICTNDKDPHMFETMINLENIILKIPSPTTPRVLIDNLNLNIKTGDRILITGASSSGKTSLLRLIRGLWPTVEGSISFFTDSIEFLSQRPLMTDGSLLQNMIYPSEXPISPDLTWFKDQLTRFNLDHLYKQYDLFQYQNRWEDILSPGEMQRICFIRVFYHRPQVVLLDESTASLPTNIEAMLYEYLIITCPKITILSVGHRDSLRKYHMKELQLQCNGEWELKQI

>maker-LSalAtl2s324-augustus-gene-1.87-mRNA-1

MEIKDNDGSKSYGFNLQFLHRFWHLQSLIFTDRINIGLAIIILLICSLEQLVMYKIGMIPGQFIRALVSEDESKFVRAIVLSLIIVCFMTLVLSARILTSELLSVSWRRSLTRCLHSLYFFNTGFYTLKLDNPDQRIASDAECLVSIYGQILSEILLSPFVISYYIYDSYRGRGSGPYGPLIIFGFFLFGTFINKTLLTPVVNSGVEVRSKEGDFRFKHAEIRSQSESLAFLGQMGSFAEASRVDHLLEILCEAQRKLVLATYRLNLATNFFDYGASIISYLIVGIPVYNGFYKGLNVEELSGIISETAFVNIMLISKFSRLVDLAGKVSRLASVTHRVAEFVEKLSQEKIKDLKDFDNHAESSPLLMNDSDDSSVSDETVLTLSNLSIGLPDISRKTLVSNLNLELKIGDNLLITGSSSCGKTSLLRVLRGLWNEKSGSYKFGRKTVIFISQKPFFTNGSLRRQVTYPLEVVSSVSHDQFDIWIKMKLIKFGLGDLIDRVNGNLDAEPDKSWSEILSPGETQRMAIIRSLFHSPEILILDEATSALSLDMEELCYREVSYNNLITLISVGHRDSLKKFHSKHLQILSEGQYSLHEI

>maker-LSalAtl2s1021-augustus-gene-0.34-mRNA-1

MPPKTNQRKKKDSSVNSELTRIAIVNADRCKPKRCRQECKKSCPVVRMGKLCIEVSPNSKIATISEELCIGCGICVKKCPFEAVVIINLPSNLETETTHRYSANSFKLHRLPVPRPGVVLGLVGTNGIGKSTALKILAGKQKPNLGLFNEPPDWSDILHYFRGSELQNYFTKILEDNLKAVIKPQYVDQIPKAVKGSVQSLLDKKDEMNNQNSVANLLDLSPQVRQRKVDELSGGELQRFACAMVCIQKADIFMFDEPSSYLDVKQRINASQAIRNLMHPSKYIIVVEHDLAVLDYLSDFICCLYGVPGAYGVVTLPSGVREGINIFLDGFIPTENLRFRQENLIFKVSENATEEEVKRMSSYNYPTMSKTMGTFKLSVQAGNFSDSEILVMLGENGTGKTTFIRMMAGKLEPDTGSGEIPQLNISYKPQKISPKSQGTVRTLLHEKIRDAYIHPQFVADVMRPMKIDDIIDQEVQNLSGGELQRVALTLCLGKPADVYLIDEPSAYLDSEQRLVAAKVIKRFILHAKKTGFVVEHDFIMATYLADRXIVFEGLPSIDTRANCPQSLLTGMNKFLSQLAITFRRDPNNYRPRINKMNSQNDSMQKAAGNFFFLED

>maker-LSalAtl2s1166-snap-gene-0.62-mRNA-1

MGPKKNKKGGKKGADEDWGDSDKALEEKMKKLMALNDEEKPEEIGASTKGDALKGKPGKKPTKMEGEEELSDAXAAPLEDKSSKKKSKKKDKKFDISKAMAEAEEAENGGTVSGEKDVTPNLESVPPEEEEDEEGNDMKGKVGMSHKEKKELKKKKKMQEEIDRISKKGGEGHSELNENFTVAQALKTDIVIEKFSIAAKGKDLFRDAKLQITHGRRYGLVGPNGHGKTTLLRHIGTRSLQIPPNIDVLICEQEVVANEMSALETVLQSDEKRTALLKECKDLEKKQENGVDVTERLNEVYDELRAIGADQAEPKARRLLAGLGFDKEMQERSTNKFSGGWRMRVSLARALYIEPTLLMLDEPTNHLDLNAVIWLDNYLQNWKKTLLVVSHDQSFLDNICTDIIHLENCQLWYYKGNYSMFKKMVVQKRRERIKEFEKQEKRLKELKASGQSKKKAEAKQKEALTRKQLKNQSKLTKEDDSGPTELLEKPREYLVKFRFPETSHLQPPFLGLYNVSFKYESQNPLFKSVDFGIDMESRIAIVGPNGVGKSTFLKLLMGDLEPTKGEMRKNARLKIGRFDQHSGEHLTADEXPTEYIMRLFNLPVEKARKQLGSFGLQSHAHTIKMKDLSGGQKSRVALAELTLSAPDVVILDEPTNNLDIESIDALGDAIREYKGGVIIVTHDERLIRDTECQLWVVEEQTINEIEGDFDDYRKEVLDSLGEEVNNPSLIANQAVVQS

>maker-LSalAtl2s530-snap-gene-0.3-mRNA-1

MDLHTRLAFVGPNGGSNASLLKLIHCGLIPSKGILRRDNQPLITRDGYDCTGVRGENADLSYQSSXILLLDEPTNHLDIETIDSCADVVNKFEGGLVLVSHDLRLIYQIAEETWVCEKNNRRDEIS

>maker-LSalAtl2s917-augustus-gene-1.21-mRNA-1

MPSDYQKKKLAKKKEAAKIKGGKKATNDENSVSKDTNDGTSSYMKDMLSNGTKKEMTNEEELCYRLENEAKLAAEARACTGVLGIHPMSRDIKIDNFSVTFHGAELLTDTKLELSCGQRYGLIGDNGSGKSSLLAVLGNREVPLQDHIDIYYLSREMPASEKSAIQAVMEADQERIKLEHLAEKLAHLDDEEVHEYLMEVYERLEEIGSDTAEAKASALLKGLGFDKEMQAKACKDYSGGWRMRIALARALFIKPHLLLLDEPTNHLDLEACVWLEEELRKYNRILVLISHSQDFMNGNYIARFGHGSAKLARQAQSKEKTLAKMVASGLTEKVATERNFSFYFFSCGKIPPPVIMVQNVSFRYNDSSPWIYRNLEFGMDLDTRLALVGPNGAGKSTLLKLIYGDLIPSEGMIRRNNHLKIGRYHQHLHELLEMDSTPLEYMMKQFPAVKERDEMRKIIGRYGITGKMQTSPIKQLSDGQRCRVCFAWLAWQSPHMLLLDEPTNHLDMETIDALGEAINNFEGGLVLVSHDFRLINQVAEEIWVCEHQKVTKWDSDILKYKEHLKNKVLKELSKDRA

>maker-LSalAtl2s1-augustus-gene-35.19-mRNA-1

MSEGEGGCGFLSAELRRLCLRRGACSLDSQSEVLEYCEGVLWRCGEDLESGSEVYDALGELLEELLPGEAEAEIRALCDELYGLLGLRTGKSRPGEGVLCLESGGSGXSEXCSPICLGESLEGAEVLDSLQGGRGSIWMEEKRGLGRVDKEKLEKAEKALLKKQGKDSSLKSHSSSQLILQATASQVLPKTRTDFNLSKDIRLEGVDVAFGDKILIQNTNLSLIHGRRYGLVGRNGLGKSTFLRMLSSSQLRIPTHISILHVEQEVVGDDTSALQSVLESDTKRQSLIQEAQHLSKEALNSYRLSEIYTEMEAIEADKAPSRASIILSGLAWQSTLLVVSHNRNFLDNVTTDIIHLQSKRLDTYKGNYTVFINQMTEKLKAQRREYEAQQDYRKHVQEFIDKFRFNAKRASLVQSRIKQPFLMPVVKEAEIIIRFPDVQKLXPPILCLNDVKFSYDGKNDIFDHVDISATMESRICIVGKNGSGKSTLLKLIMEEISVTDGRRIVHRNLKFGYFSQHHVDQLNMNLCPLQIMEKKFRGKKMEECRQMLGHFGISGDLALQKTSSLSGGQKSRVAFAVLCGEEPNFLILDEPTNHLDLETIDALGHGLMKYNGGLILVCHDERLIRMVCQELWVCSKGKISRLEGGFDEYRRILELELEI

>maker-LSalAtl2s467-augustus-gene-3.18-mRNA-1

MMSSDIKMESESGWRSKAMITNEYVLELTNVFHSGQVETGTCMQKMIGTVRTGVILKDVSMELHGGELSAVLGSKGSGKHALLEVISRRAQGPTRGQILLNGVPMSMRLFQESCGXVTQKXDLLPGXTVKETLEYSANLTVGSKVGSFVKHSRVKQVMADLALTNLANRNSESLSQSEYRRLVIGXQLVRDPVVLLLDEPTWDLDPLNTYFIVSILANHAKKYNRIVLLTMEKPRSDIFPFLDRVTYLCLGDVVYTGATRMMLDYFRSIGFPCPELENPLMYYLCLSTVDRRSRDRFIESNNQIASLVEKFKMEGGPYRKYGGPPPDAESVLDAASHQKVPLTAYGRPNSITIFYYLLMRSWCRISPFNIHGMQQFFIKILMMPTFFFLLWIFYYNSSSKGMENQYQRNFVTRNGLVFNSLAGAYFMSILATVTSFATDRTRYYQEVREGIYGGPLFLLSNLVQSLPLSALTTFMSTFIIFRGLKNELICYPDGDSNICKSYSSFDSDLDDLNYHLEYSYYPDLITHWLXLWACYLLAEQQTVSILMVVKSSYTATYGNGTLFLTEKYGYKHDRLETIMDRWFNIGVSILFPAILFLLNNVLYLIPLPAFVKAKFRE

>maker-LSalAtl2s467-augustus-gene-3.17-mRNA-1

MWEMKAMDGRRYNKTGSSMMSEVQTGGGGGGGHXLPHGXASTSEDLHAWSIFRQNLNSDFTDSALGSSEKSPMPYGNFHLRESTMHSILSNPKYGPKSELGANMYTYLKFGLPRVLPPLHKRENSSGYDSTDEETHHHTSKNRSNGVLRSARSEDFLNYGREEIQFATNYKRNRDRHPQSAINHRRLGTSSNSTTDRRXKSASEANLLSPTSYYYHQDDLSGTKTGRLRSLARSRASSTNRISSEKDLRDGGAFVNRGMEMDEDDDEEEEIDTELDKDSVMANNNKLGGNKAASTLSILSKSKINGVSYRNEAPMVNDKYFPKDRIYGSNGNFLGGGLHGADIIREGTKYPHLQIRGLNFEIRRYGHFIRLLDDISLDVKGGELXSIMATKEDEGTALCDIIANSFNHWNTRLDTDIILNGISVNTKRLEDRVSYVKRNINFSPDMSVRQTMLFHSFLREPGTHTRNNDTKGRINALIEDLGLVQVKHTRVKDLTVSERQRLNVAAHLLMDTDIVVLDQPTRGMDIFDTFFLVEYLRQWAGRGRIVIITLHPPTYEILTMISKILLISTGRSMYYGKRREMLPYFALIEYPCPAFKNPSDYYLDLVTLDDLSSEAMLESSQRIDQLASTFKRRMEPLPDPGPPGVLPSKIKRANFLDQIFGLWIRALIYMYPFNVIEWVKMVLLSGGISILVGVIFLGIRWQYWDREWQENPTFDQDNINDRLGFHHVMMSVGIWPMMMAMITNEWANKMPISRDVDDKLYSKMAYIFIKTLYSIPGIVGIFLAYIIPGYLLAGIHYQNVNDLDVFYYYIGYMMLYLLSIRMMIMCFVHLSSSRHWASAMGGTILVILSLVNGYVIHVKDLGDWTSWIKYVSPQYWMNHPIQRGEFSPISIFHCKDNPVITENSIIKQVPCGLSSGNKTLDYFQFGDKFQNIVRAPWYIFMPIFLTLFFYAFWQILCYVFYLGRTQKARQSRSRKSKV

>snap_masked-LSalAtl2s1226-processed-gene-0.1-mRNA-1

MNGPVNPICEDDDSSFKNMENEPRIVVALRDACKYYGKNAMENRVLNNLNLTITEGSIYGLLGASGCGKTTVLSCIVGRKKLNKGQVTVFGGSPGDKGIGIPGNRIGYMPQEIALYKEFTVRETLKYFGRLYEMETDQIDQRIDFILKFLQITRDRDMVGKLSGGQKRRVSFAAALLHDPELYILDEPTVGVDPRLRKSIWNHLTDLALNRKKTILITTHYIEEARQATSIGLMRNGKLLAESSPEKLLQIYGEPSLEDVFLTLCVSQDELNSIESFNYSTSFSNSLKKVVEGIFGIFRKKPPESIMISSTKISNANEDGHTSKGVYPVSYNKSSESIDSYSSTKPTNIDPKLLSNFSKNKFGNHRILSPKKLKALIMKNFIQMWRNIPNLLFIFLLPAIEVLLFCIAIGNDPTNLNFGIVNNEFPYASNASNYSCSTLQGCEFENLSCRFLSNLTNRKDLNFKYFEDEQLAKTEVLNGEIWGYMTISSNFSEAFLDRLWNTLNVDSESLLQSSLRVYLDMTNQQVSFSIKRIIFDSYKDFIGGLMTDCELPSELAXSPVRYEDPIYGVENPSFTSFMAPGIIVIIIYFLAMALTGDAFLLERRDGLLDRSWVAGVSALEYILAVILTQFIVMIIQTIITLIFILIVFQITCNGPLLWLIVLTLLQGTAGMTFGKL

>augustus_masked-LSalAtl2s1118-processed-gene-0.6-mRNA-1

MTDFEGSEMDQLQIMKNDNEHDPRAAISVKGAYKSFGWGKKKVNVLVNLSIRITKGHIYGLLGPSGSGKTTLLQCVIGKQSLDSGSILVFGEYPGTKDLGVPGKRVGYMPQDLAMYMELTIMETLEFYGRIFNMPKAKIKKRAKFLVELLELPKKKILIQKLSGGHQRRASLAVALLHEPDLLILDEPTVGVDPVLRRNIWGHLVDICNNPIRKTTIVVTTHYVDEARQANMVGMMRFGRILAQNCPSRLLKIYNKPTLEAVFFNLCVRDESEXIYLLM

>augustus_masked-LSalAtl2s100-processed-gene-0.5-mRNA-1

MKKDFDPLYSEKNPLALLKAKLAIWIRNAYKHFGTKSNTSLFTGLNMSVPKGVIYGLLGPSGCGKTTLLRVITGREKLNSGEFKIFGEDPGTVGLEVPGRRIGYMPQDLALYMELTILETLFFYGRLNKMQTSKIRSRAETLIEILELPHSGRLVQNMSGGQRRRISLAVALLHEPELLVLDEPTVGVDPVLRQYIWDHLLTIVNNSSKKRTIIITTHYVEEARQAHIVGMLRYGKILDEDAPTKLLEKFKALTLEEVFYELCQQEQRVICEGSEDEEESFLMSRNQRNSSLSSDSKFRRRDAFSISIEAPKKHFFPQMSSFFAIHRWKALVIKNFLRMFRKIGFLIFQLIFPAIQASAFCIAIGKDIKGMTVAVVNEEATYQECQNNYAGCILSFQSSKYVTMSKTSDLQDNLSCRLLYYIERGELDIKYYDDFETARNKVLNGDHWGVIHFEKGFSKKLAEEMHFIVSEPYAKDKEINTRMHIYLDMTNQQISLSIKQSLHSSADEFLKESLRACNKSEELGNSLLYFGNTIYGSNKPSYTEFMAPGIILSIAFFMAVGLTSQSFVTERREGLLERSFATGVTTLEVMLAHMVAQFIIMILQVTFVLLFMILVFKIPANGSIVXMISXVILQGLCGXSFGLLISSFCSTEHDAIQLALGSFYPLLLLSGIIWPLEGMSKNLRYFSYTLPQTLACKAMRGILSRGWNLEWSIVYLGFIVTIAWIFIFQILSLLLFRIRK

>maker-LSalAtl2s100-augustus-gene-0.13-mRNA-1

MMRYGRLLAESPPANLMKLYNKPTLELVFFNLCRKESSDDSLFAEHSTPRTPKHNPDVISLKQIQTSSEAVAPSFSEQKSCLPRMDSIFALHRLISLVIKNFIRMWRNIGFLIFQFILPTLQVSLFCLAIGGDLKGMSLAVANEDIGSKTCTGFAEGCSISENPLDYLSFSDPVIHPAFNLSCRYLSFMDKDAVDLVYYDDYASAKQAVELGKHWGMLHFTPRFSKAFPDRVIKLISMEVPSNQTLFDSQVHAHLDMTNQQVGHTLKMIMTLSFQSFVEDILQSCNKSQKILGFPLHFHEPIYGAMEPKFTEFMAPGVILSITYFMAVGLTAQSFILERKEGLLERSWVAGTATEVMLAHIIAQFAVMVVQVGFVLLFMIYVFSIPSQGPLFLIILLTILQGICGMSFGLVISSMCNTEQDAIQVALGSFYPILLLSGIIWPLEGMPRELKXVSYALPQTLACEAMRGVLSRGWNLEWTQVSQGFLVTIAWICVFQIVSAIILRIRR
