# Supplementary material for: A Survey of the ATP-Binding Cassette (ABC) Gene Superfamily in the Salmon Louse (Lepeophtheirus salmonis)
Source: PLoS One. 2015 Sep 29;10(9):e0137394. doi: 10.1371/journal.pone.0137394 (PMC4587908; doi:10.1371/journal.pone.0137394)
Supplement: S2 File — (DOC) [file pone.0137394.s010.doc]

>Lsa.1758

GGVYYYANILIFFIFFASYGISTICFSFFVSTLFSKSNSAGFSGGIIFIVTSFPLSLMKNVKVEFNTKMVACLLSNVAFGTGLDYINAYEKTGFGIQWANFMKTPFDSDNFSLGLSIGFLWLDAFIYLLLAWYIGNVFPGEFGIPKPFHFPFSISYWRGTKPALVETNSKENLDDSSQNKKDIEEEPKHLPCGISVQNLHKVYPKGKVAVDDLSLNFYEDQITSFLGHNGAGKTTTINIITGLFPPSAGTIKVYGRDIQKETESVRRYLGICPQHNVLFQYLTVKEHFILFSLLKNTDNRADDPEIDSLISEIGLNKKKNAFPAQLSGGMKRKLCIGLALVGGSRVIILDEPTAGVDPFSRRSIWDILIRYKTGRTIILTTHFMDEADLLGDRIAIISEGKLITCGSSLFLRNRFGNGYYLTIDRYIPEDNHHEVFSSIEDEEEDKELNDCSLPNGMLIDDEGISDVSKVNGVIINPSSSNPRARTIALTKFIQKHIPDARLFEQLGSEILYLLPTEDYENSIKKFEGLFLELENHLKTFQIRSYGLSNTTMEEIFLTVVKNTNAMEVQRQPVYDGKSSSRHRLTSSSSLPAKNYSIKEPKHESNNTHTSSVEPIIDTNIRIGYEDYEKVDDTPEVYTKGTNERKKLRLPRIKKRAVVKHAIALFIKRFHQHRRDKKIVLAEIVIPFFYIVLSAMCISRYPEETSEPQLPLNPWIYPITQDFNTYTFYSNAHKEADWPRRYEEQLLSKIGMGDKCVTKIDGSHNCTGGKPDTSNFSPDSSMDYMEISPVCKCQTMYEACDKPPPNYYPPEVKLPSGDILQNLNGRNISDYLIKTRFEFEGKRFGGFEFGILNPLAGRNFDQWADSFQKISKATNMNESRVDAFTSTFAKEALVDMAYSSSTFDYIRVWFNNKGWASSVSYMNAVNNMVLRATIEEKSERMHELVDSSKYGIAAINHPMNYTSDQFSNHQLNGIVEALLNSVNILLVLSILPASYVLNYVLENNLKIKHLHFVYGVKITTYWITGYIFDTIIFCINLVFITVALIAIDASSLISDANFYGFATLIILYSLAIMPLMYILSFFFKKQSSALFALLNLNLMIGIIPFLTTLLVKYVNEDEKLYYVLETLFMIFPQFCLIHGLFQMFIENIKSITYEDLGFTSTASTFDWNYLGQNYLFLFFEAVIFFIANIMMEVRIFESWRLKILPDAFTKAQKETDDEVMDEDVYEEYQRVMNAYNDPNNDENALLVKELSKRYWRQKNSAVDKLSFGVRRGECFGLLGINGAGKTTTFKMLTGDINPTSGDAFINGYSIFNEMGRCRQSLGYCPQEDALHPLLTGREHLELYSRLRGVNKKSEIKIVNYFLKELGLISYCDQLTHTYSGGNKRKLCTAISLIGKPSTVFLDEPTSGMDPGSRRFLWNCVLDVIRGGQSVVLTSHSMEECEVLCSRLGIMVNGKFKCLGTAQHLKSRFGSGYSLTIRSGTTDGNLDSLKDFVLNAFPFSEIKEEHYNQLTYQIPLKTIKLSTIFHEMERAKSMKHLLLEDYSLTQTTLDEVFIRFASEQKGMSEDAEFVNDEEGPETAVETTAL

>Lsa.14583

VVYVFDSPIALVIIINMLYLHHTKLLLWKNFKKRSREKTRTILEIFLPLALFILLVFVVRNNGMENIPSCHFEEKSMPSMGPELFIKSFFCGFKNTCNESPPRDSSKMSAYNVTFVNRLLSDLEDSLYKSFNEERAAAFSKIINDISSIRELSNKAKKRFAQGLSMQGKQMNSIW

>Lsa.1680

IGFNVSRLSVLLNDEEEFIGSMNTWMKIKNISVKENVLQSLLDASIDVSNLSGSDILSIIGNPVSVLCKPSVTSKLFHFRQKLNNPIQKELDGICNLNVSELRELVILANGHFNRDSLQREFDELMNVHGQINPFNITDWSLFIKTVQELQNNLLNQSSFKSVMGDVSNILREYSESVDFGYLREKPSYKMKLETQIALLYRLLCSRKRSSIKNIFSKEGEASYLDEFRESMKAEKKEEFEYLYDETTSKQCNEIMQMLENDPIFALIWKQLKPLVRGKIVYTPDTPATRKIISAIEKTFFPIIKTIKIMELWTNQYSSQFRAFFLNGENQNFIEDLFTDDSEGNFIDLILNNRIVDVLNGEGEYHVVNASDVRQQVNEYFQSDISKAWAKSFDGLDELLENLSVYLSCFETDKFVGVSSEYELEKMGLNLIGNNNLWAGLVFQDFPPDQKDDTLPEFITYKIRMNSVTVDNTRQIQDRFPSLGPRKNPTVDLKYLTFGFAFLQDMTEHAIISIHSGRESSELPGISLQQMPYPCYVKNNVIDNIGELLRTMIFLSWIMPVSGLIKSIIYEKEARLKETMKAMGMGSAAYWMSFFLDSIFITGPTVVILTFLLTV

>Lsa.26127

NLRIVMLSRLLRTGVVTLGRRNISFSRPKGKEWKKLFEFAYPERKAIGGAMGLILISSGVTMSVPYAIGKIIDIIYELGDRNNSDEEKKKASTRSRLNSLCTALVVVFGIGAICNFGRVYLIQLSGQRITARIRSRLFSSITKQETAFFDTNKTGELVNRLSSDSMLVSQALTSQISSGMRSSIMALAGGGMMLFMSPQLALVGLSVVPPVAGWAVWMGKKVKNISHEYQNTLADATHLAQERIANIRTVRAFGKEVQESMAYDEKMSLVLDKGVKEALIQAKFYGMTGLTGNLIILSVMFYGGFLVTQDVITVGNLTSFILYSGYVGIGLNGVSSFYAEIMKALGAATRIWEIMDRTTHMPLDTGLILPMPLNGHITFEKVGFSYPSRPDHSIFDGLNLNIESKQILAIVGSSGSGKSTLTSLLLRLYDPNMGRVCIDGTDIRELNTTWLRNQIGIVMQEPVLFSGTIKENILYGTEGKIEHEEIVSAAKESNAHDFIMNFPDRYDTLVGERGVLLSGGQKQRVAIARAILKNPQILILDEATSALDAASEHEVKEALNRVMKGR

>Lsa.7262

ILCSSVMLFRLCGSSMARMSSRVLLKPKKGPLALWSKNCFTPGVSGLARTGIKVVPKDKVGPKEIFSTMFHHIWPKNEPQVRKRVMFALGLLVTAKLLNVSVPYFFKKAVDVLNSEANDYFSLGTPAETVFAMASAILIGYGCARAGAAGFNELRNAVFARVAQRSIRKIAQNVFLHLHNLDMSFHLNRQTGALSKVIDRGSRGISFALNAMVFNIFPTIFELGLVSGVLGYNFGANYAFTALGAVGMYSVFTLGITSWRTQFRVNMNKAENEAGNKAIDSLINYETVKYFNNEAYECQQYDKSLLKYETASLKTTESLSLLNFGQNAIFSVALSAIMMMAAKDISNGALTVGDLIMVNGLLFQLSLPLNFLGSVYREIRQSLIDMQVMFQLMATPSKITSSKGATEINFINNMEDASITFDDVKFGYNIDKNIANGLSFSVESGKTIAIVGESGSGKSTLVRLLYRFYEPQLGSIKIGNYNINEVTLDSLRRQISIVPQDCVLFNDTIFHNIKYGNLNCSDEDVYKVAKLAEIDNAIRSWPQGYLTQVGERGLKLSGGEKQRVAIARAALKDSPIIIFDEATSSLDSITETMIMKALKRVTSGKTAIIIAHRLSTVVHADEIFVLSNGKVIERGTHEELLNTRESRYSVLWESQHWHERTRLEKMINK

>Lsa.643

SDLFTQNPTIKMTLALFRAATQCCFRISRSSEGLKSFTRKTLPIILGGSTTLCLVGFNLRNVKAEAPSKREKEILPSRVVEAKSEKLSSTDPSNDHPFPWIKFLGYITPHFHYLICAVCSAIAVAYFNIKIPLLLGDIVNVVSSYISETVLSEMRHDNFLREMKEPTLSMIKFYAAQSICTIAYIYSLACLGERMAASLRKDLFNSIICQDIAFFDEHKTGEIVSRLSADVQEFKSSFKLVISQGLRSSAQASGCIVSMYMISPQMTSAMGVIVPTVILGGTYIGSYLRVLSKKAQAQVAKATAVGEECISNIRTVRGFAMEDAEMELYSREVDKTRDFNEALGLGIGVFQGASNFFLNSIVLGTITYGGYLMSDDNLNPGQLMSFLVSVQTIQRSITQVSLLFGHLVKGMASGSRIFEYIEKVPLIPISGGVKIPYHSFFGDVEFKNITFSYPTRPEQAVLKDFSLRIPPSKTVALVGTSGGGKTTIAALLERFYDINGGGSLEIDGINIRDLDPSWLRGSAIGYINQEPVLFATSVIENIRYGRPNATDNEVYEAAKAAHVDDFVRTFPDGYSTILGERGVTVSGGQKQRIAIARALLKNPPILILDEATSALDAESERIVQEALDKLSKGRTSLVIAHRLSTIKNADVIAVIDKGVMAEIGTHAALKRKGGIYSRLIEQQEFRE

>Lsa.4043

ITYVDPQIIIMRWNKYIVTPEKERKKMPDGSHMNGIINSGFEEIELNDNNSVLNPEEEKKNGETVEHKSSVVSNDNLKLDINEDISNTHVPFMKLFSYATRSDLIFIGIGILAALIGGLSLPFMIMLFGELTDTFILSNPLSTDICLIENGTCCSNNGTVDLSLEDCDLNEDDIMQLFKPINFLDGVARFGQGTAIIGLINFITSYIFVTSLNFTAERQVHRIRKAFFKSLLNQDIKWFDTHETGDFATKITEDLNKLQEGIGEKIGLFIFFITIFISSLITAFIHGWELTLVILSAMPILMIAVGIIAKSQTALTVKESNAYSKAGSVAEEAFSSIKTVMSFQGQNTEIQRYKENLSEAQKTGILRGLLTGIGGGLMWFIIYSSYAIAFWYGVKLILDDRESCIASPTDCQIRYGPSNLLIVFFSVLMGAMNIGQASPYVEAFAIARGAASSVFQIIQSTPAIKSDYDHLQRQQDKAPPFTGRITFKNVHFEYPSRPTVKVLSGLSFEASPGKTLALVGPSGCGKSTVIQLIQRFYDPSFGVVSIDGEDITTLDPHWLRSHIGIVGQEPVLFEYSIKENITMGLQGEISEKMIDDTCKAANAYDFIQRLPKKYDTIVGEKGALLSGGQKQRIAIARALIRNPSILLLDEASSALDSQSEFIVQSALDKARKGRTTIIVAHRLSTIRSADAILVMKDGYRVDYGTHESLKSNKTGLYCSLVNAQDCQVDQDEGLPLFNPELNYEEEDEVYDLEQVENEMNSMTYGSISGGSSWNRRHHFVRPTLERRHSTGSGYSEDSLKIEDALDVAGSAIGIARVGSRKIRRTSTNFTDNEYLEAEEMKSVNSNVGFFTVLRENSKEWLYIFMGCIASVVMGASMPVYAHLFGEVLGVLSKSIEEARVNSITYSMYFLLVGIIVGFSMFMQIFMFSLSGELLTTKLRIKAFTAMLNQEVGWYDESVNSTGALCSRLSADASAVQGVINYLSFFIHLLYILIATGSRLGTIIQVTLTILMSISAALYFNIKLGLVGTLFVPFVLIGAWFQGKIITSQDNLEKDALSRSARIAIEAINGIRTVVGLRLEESFQEKYSTELKDPHESAIKNSHLRGLIFGFSQSIPFFAYAGTMYYGGTLVESDGLPYKNVFKVAETLILGTLMVGQATAFGPNYTKARIASIRIFKLLNREPKIRSDVIPNTDEMATNMNGEVTFTNAGFYYPTRKSVKVLRDLKLSIKSGQSIGIVGSSGCGKSTIIQLIQKFYDLSSGKLELDSKDSESINVMWLRSKIGIVSQEPNLFNRSIRENICYGLNKRNDVSMDDIIQAAKDANIHSFIASLPQGYDTRVGNAGTMLSGGQKQRIAIARALIRNPSLLLLDEATSALDTESEKVVQEALNKALENRTSITIAHRLSTIKNVDKIFVLNQGKVAEAGSHESLLLLKGFYYKLWTNGTQRKI

>Lsa.11278

LKSTLDLIREVGKVDVRELNLESTVMGTSTFCSDPLWNSNLTWYTEDPYFTDCFISTVLVYVPSGILFLLTPYEIWTCFNLGENSSRISRTWLNFSRTILVGFLILLSLVEFIFELLREDNVLSNIVAPGIYFLTFLNVFILQILGRIKGRVSSGPLFVFWLLEVFAAGFSFRNWRNIHRVPNTDLVTLSTIVIQYPVVVILLFMGFWADGNNNYNKIGDNDLNPSSLRMASFTSKLTFSWFDPFIYKAWKKSVTDDDLYEINDEFKSCGVLPSWDREMEKEIRRKHLKNKPINILWPLVRSFKSTLLASSALQFFYSVFQFGAPQLVDLIINFVSDPKEPIWKGYLYLFAICSITFINTLIYSQSNYYSYITGLKIRTSLTSSIFNKAVNLKSSSKKQMSVGETTNLMAIDSQRLMDFCLYIDMIWSSPLKIIIAMYLLWQVLGPASLAGLAVMILLIPINIVVGKKVKKFTAIQMQNKDKRIKLMDDILNGIKVLKLYAWEPSFIDAVTNFRVQEIGALKKSALINAVTSFMWTSAPFLVALASFTTYVLIDEKNILTPSTAFVSLTLFNLLRLPLNLLPMMIMKAIQSKVSLDRINLFLNKEDLDLLAISHESQGSHAVKMCNVSLSWETDEGLNTLEDIDLDIEKGSLVAIVGQVGSGKSSLLSGILGEMEIVRGSINVDGQTIYAPQQPWLQNETLRNNILFGKRFNKKLYRRVIDACALSPDLDMLPAGDLTEIGERGINLSGGQKARVSLARCTYNNGDIYILDDPLSAVDVHVGRHLFENVISSQTGLLKNKTRIFVTHGAIFLPQTDKIIVMKNGVISESGTYRDLIEKENGEFAQFLINYLVDEKENILKNDQDLKTIIKDLESTVGGSEELEKRLEAAQSDKSTALTDLIQGTKYLDENTSDQCSSIASRKGEDDLDEVVSNDLNERLIEEEKIESGGIKLNIYLNYFKRTGLLASFFGILFYFSYQGFSLGANLWLSKWSTDRAAINSTSIRNHYLEIYAILGFFQSIFTMLGSVTIAIGTLNASMKIHGKLLENVLRAPLSFFDTNPLGRILNRFSKDIDVADTTLPFNIRMMIAQSFNVLGTIVIICIALPWFFVIILPAVICYALIQKFYISCARQVKRIESISRSPIYSHFAETLTGIPTIRAFGMVHHFIDENVNKIDFNAKCYFPTVISSRWLAVRLETLGNILVIFVALFSITSRGTTDPGMVGLSLSYALSVTTILNMLITVSTDVETNMVSVERIKEYENIPQEAPYDLPNSDPPPNWPEHGVIKFDNYKTRYRKGLDLVLKGINCTIQKGEKIGIVGRTGAGKSSLTLALFRIIEPSEGSIYIDGENIRFLGLGKLRSRITIIPQDPILFSGSLRMNLDPFEAFADRDIWIALEYSHLKSFVWNLGDGLNFSVLEGGRNLSVGQRQLICLARAILRKTQILVLDEATAAIDLETDDLIQSTIRSEFKDSTVLTIAHRINTIMDSNRIMVLDAGTIAEFDDPQNLLANPDSMFYSLVNDSNGMGNKNFRS

>Lsa.22810

LTIFLSFQVFLWNCGIFIFSFSAFATFSFYSHQMSVQSIFVSAALVNTMRIPFRLLPSCISALSQGIVSIKRIDSYLNREEVDSVKSNDFSKSPRPEDGYSIQVQDCNYSIDKLKLLKNINFSTRVGELTAVVGKVGSGKSCLLAALCGELNQRGKGSSLINKDLVYVTQNIWLKSATIRDNITFGQPYNSSQYQKVVSLCQLGQDFKDMYRGDLTYLASNGSTLSGGQRQRIGFARAIYQNAQVYLMDDPLSACDSNLKAQIFHNTIGPKGFLQKKTRLLITNQSSLIPLMDRIIVIQNGTVVYEGTYDQFKSHFSSVNEFLEPDETQNEKIEEEKDSAEEVKSFEIAKKSTKKQGSFHEPPKDNYLHPALDNYDIIERRKIPFSLYKYYVLNLGTVPFIIALVGYLISQCFDVCSKLWLSRWTNLSSTNINDTNYRHIYNEDTRNMYIVVYGILGWCQSLAYFLSVLLINSRSLKASSHFHDVILRKVFGAPLNFFWSTPKGTIINRFSKDMDEADMFLPNTLKNFAYQAVKILGTLIIALIAFPITAFFIVCPLGLIFIEVVKSYLMASRFLKRTSATKLAMVLKHFGDSAVHGGTTIRCYGVESDYIDEHMSLIDEHQSVSLMEIISEAWLFLRLQLIAGTFIGILATALVFFPSEGNSSSLSALSLTSSLTVLQDIFLFTRYAAFIEKAMVSIERIKECEDLIPQDPSSGLLPSIITNGSKRPKNHQSRYYIQFEDFSGKYSSLKNASIKNFTLNVRLGEKVGVVGRTGSGKSSLILALSGLLEVEKGKIFIDGVDMSSGTARNIRKKDITVIPQDAALFKGSIRYNLDPDDRFTDEYIWSVLDEAQMKEFFVQLPGGLDFSLKENGSNISLGEKQLICCLRGLLTGSNLIIMDEATSAMTLESEERILNTYFSRFSSSTIFVIAHRIQPLLACDKILVLENGTIAEFGNPESLIKSQKSIFKSMLRRTNGSVE

>Lsa.29272

RTFNIHEKWKRKDSCSPLCNKTALALMKLLSSEILISGLYEVFHILFTLTNPIALKMLMDYIEKERGDYLRGIYSILFLTVTGFLSSLCETHTFYHLNLSGFIMKTALMSAIYKKSLRVPHFNGGNVISLVSVDCQWLVKAIRFIHLPWSCPLQIIIAIYLLYNILGVAIVPGIIIIFILIGISF

>Lsa.6310

FIVKRFVEIHTQKMNESWICEEPLWNQVSDSWKTLDHPNVSSCFRIFFFRLIFPNLCFFYVWLPFEVHPIYSSRDKGIKRSLLGTLKDLFAFFCVSVAIYDVIHEAFFKPNFQILEILDPSLRILTYTLVLVLNFLYRVKGMRISGFLCRFFFVSLFSFGIDLYLMYEVMTQLKLEELMSSIIHVTLLLLNVLAHFWPEPPPRYSEYVRLVGVEGCHNPSPDTQCGYPSYIFFSWMSSLMWKGFKRPIKQKDLWDFIPSVNTALVNPHFLKHWNKIYTQTKFSNYPTSKSPSIFPALLRSYWPLLLKGAFAKLLHDLLLICSPMLLQYLILFSYSPDIPLGQGIALSVILFGNKILGTFFIARYFFYMVTVGMKLKTSITSLIYRKALRIHSTRDDTTSGEIVNLMSVDVQKIVDLMPLLNTIWSGPLQIIAAIYLLINTLGYSALAGVLVMFLLIPINGFIAVRMKNAQMKQMKKKDERVKKMNEILQGIKIIKLYAWESSFSKLISSIRFEEIKLLKLSSKYFGFMMLTISSTTFFISLFTFIVYVVSDPENHILDSEKIFVSISLFNILRFPLNMLTHVIGGIASASVSLKRINKFLASEELEEGILVRSNSHDHQAICIHPSSSFSWKGSSESLFKDISMDVNRGNLIAIIGPVGSGKSSLISALLGEMLFTSKGTNPVEIHGSVAYTPQNAWMQNVSVKDNILFGRKFNKNWYDEVLKACSLESDMEVFPSGDSTEIGEKGINLSGGQKQRISLARAVYSQSDIYFLDDPLAAVDSHVAKHLFEEVIGSNGLLRGKTRILVTHNLSFLHLVDEIYFLKDGEITEKGTYKELMEKNCDFSELISNHANNKITNQSDSEKVMEKINEEGTKQPIETNFKIYDEETLHTGQVGIRVYWYFLSKIGMLAGILAFSFFIISQILATGSSVWLSVWSDAKNNTDIYYHLGIYAGLGVSNIFISTAGTLILSLSLLDASRILHGTLFQCIIKTPMSFFDTTPLGRILNRFGQDIEVLDTKMSFCIFGSMTGTFAYIATVTIISINVPIFIIPTLAIFVVYFVILIVSLSSSRQLKRLSSVATSPIYSHFGETLSGINTVRAYSLEKAFYSEFERRLDEYQKRNFPVIMADRWLGIRINLIGNFIIFASALFSVLSRDTITPVISIRLFVFLTSVLLF

>Lsa.8882

KKYKMEQLNQDEEANSTTMYLNGFCSDRDPFWDPDYAWNTNDPNFTSCFRKTILVWIPCLFFWAFLPFHIYKLYHSKARKVPLKTLGRVKALSCFILILIAFVDLGYWGSKEFYPKDLINPIIRAITFLAVFILLFVERARGYRISPLLTSFFLIYFATNTIDLYGHIRWVMMSGTTSLADITFFLHFFCIIVSFLCHFFVEPRPLYEDTAVNDIKSNNPCPLITSSFPSQISFSWLDSLLWTGFKRNLSFDDLWDLVPSLSSRTVVPIFLQRLDSALRRVKTNENGITYAKGDDNVEVKTKHEKKPQLSILPALARTFGPSILSAAVIKIVSDSLNFVPPLILKRIIKFSTNQEELWKGILYAVILFASSTVGSLVLSKFFYKMYVVGMKIKTSLISTIYRKALRVPTSTKKNISTGEIVNLMSVDAQKIVDLMPYINTVWSAPFQITIAIYLLWQTLGPSVLAGVLVMILLIPFNGFIASRTKTLQTNQMKEKDERIKLLNEVVQGIKIVKLYAWEQSFLDIISNVRSKEVKILTHIGYLQAGNSFIWTCAPFLVSLVTFATFILSSPENVLDSEKAFVCLTLFNILRFPLSMLPMIIGSMVLAGVSIKRINKFMNSEEIDEEAVEKKTAYPDDKFAINLEKACLKWESDEDKNILSDVSLDIEVGSLVAVVGTVGSGKSSLLSAILGEMDKVSGLITVRGSIAYGAQQAWVLNTSLKNNILFNKSYDGDKYTRIVEACALKSDLDMLPGGDETEIGEKGINLSGGQKQRVSLARAVYSGSDIYLLDDPLSAVDSHVGKHIYDNVISSSSGILKDKTRVMVTHGVTYLPFTDKIIVMKDGRVSEIGTYKELLRQKGAFAEFIVQFLSEANENEVNENIKHDIEESFGKNELLEQITKAKQIVRERSVSLNSGDLVTSLVKNNPTNSASGSTTSLKENSGDESTDNDTKQTRPTTSQTQQYQDEKVETGSVKWNIYMHYVKNMSIILVVACSSCFVIYQALNTLSNVLLAWWSDAVMKIEVRTGLNDNRTLDEINQDIMDTQVYYLSIYGGYCLGQGIVVVMGFVFMYLACMEAAQRLHNQMLESILKSPMSFFDTTPQGRILNRLGKDLDVLDSVMPMVLRGWISCFLGVLSSLIVVMVTTPVFIIPASLIIICYFFIQRIYVATSRQLKRLESSSRSPIYSFFSETVSGAATIRAYGQSSTFISESESKVDDNQKANFPATVSNRWLAVRLEVVGNLIVFTAALLAVLGRDSLSPGLVGLSVSYALAVTASLNWLVRMASEVETNIVAVERIQEYTKAEKEAPWIIEDTKPDEDWPRKGEISFKNYSTRYREGLDYVLKNVSLDIEGGEKIGIVGRTGAGKSSFTLSMFRIIEPVTGNIIIDGVDITKLGLHQLRSRITIIPQDPVLFSGSLRRNLDPLDEYNETNIMEAISHSHLKPFIDSLKDGLEYHVSEGGENLSLGQRQLICLARALLRRTRVLILDEATAAVDLETDNLIQKTIRSEFKDSTVITIAHRLNTIMDYSKILVLKNGERVEYGTVAELLADKKSQFYSMCSDAGLV

>Lsa.23107

LHYFSFASVIATLSFLFCVLLSVFVCVLADSLLYIDNMDIEEKVNLKDNKRSNANILSKIFFIWVLPLLRDGQNKAFDIPDLPSALTEDKSRYLSDNLEREWKKELEKGLRYDNTSKKRYSPSLLRALIRTFGSSLGVYGAFSFIEECVFRLLQPLAISQIVLYFSNSNHGISPTQLYIWSAVLIMSGVLYVFSHHWYFFGVVQVGMRIRIACSALLYKKSLKLSKASIGKSSVGQMVNLLSNDVNRYDLCVLFIHYLWVAPLQFILVSIITWYMVGISSMCGGAILLVFIPLQTWIGKQFSRLRILIAGKTDKRIRVMNEIIEGMKVIKMYAWEYPFMEVVNETRRDEIQTIKKTYEYKAFNLGFFFTSSRVVLLLIFFLMIIGNEVISSKNIFLIFGLFNTVRLSLTLFFPNTISMTSEALVSTDRIQNFLLLEEIGDICSSMKHEPEVRPEVSLRIEMKNVSGKWTSNEKDDDLRNVSFQVHKRELTAIIGPVGSGKSTILQALLGEFPVSSGDISIYGKISYASQEPWIFSGTIRQNILLGASMNHKRYLKVLKVCSLEHDLESWPDRDHTFVGEKGVALSGGQKARINLARSVYSEADVYLLDDPLSAVDSHVGRHLYEECIKNYLSRKTVILVTHQIQYLGDASNIILLNTKGEIEDQGTLNKLLMSERDFTSFLVAQEEETDSIFDEDELALTPKKSLNIENYMRRRQSSVSSIGSRATIDTNAMEYNDIPNEGRRTQTTESKVKGSISVALYKKYFYAGGGKWIFFFVYGLNILSQLLFVSTDWWLKQWTNAADARTKFGDISNQSHFFSNYANASSFEILPGFQIDLFNSIYYGIYFALVGALIICSQISIRKFLILCLKSSKNLHLEMFQKVIFTKPAFFDVNPVGRILNRFSKDIGSLDDLLPLALSDTSLIFLNAVGMFGLIISTEPKVLIPLGVILIILLILRKYYLNASRSVKRLEGITKSPVISQLSTTLNGISTIRASKLENTFSSEFHYLQDIHTAAFFSFQSVTRFFGFWVDGIVSVYVAATVVIFVFFSGDVEGGDIGISLSLSVIMAGMIQWGLRQSAEVENYMTSVERVTEYADLPSEKSLTSDKKIDPSWPNKGVIKFHNVKLKYDDQGGPYILKGLTFTINSFEKIGIIGRTGAGKSSMIAAIFRLVEPEGEIIIDGEDICQLGLHDIRKRISIIPQDPLLFSGNVRKNLDPIMEYEDSDLWNALEQAKLSQIISNLNGGLDAQVTDGGSNFSIGQRQLMCLARAILRKNRILIMDEATANVDPHTDSLIQEAIRTKFSKCTVLTIAHRLHTVMDSDRMLVLSDGRIEEFDEPHTLLQNEHNLISHLVEQTGPAMSEKLRNIAKLHFEERRNNQEHHPGSIEEDNLVMTHL

>Lsa.3521

LNWISMDFQQEYFQETNPKSKTNILSNILFLWTIPFIRKGQKEDFDVHDLYKTLREDYSRELNDRLEKEWQKELSKVKTFSGNYHPSFAKAAIRTFRLQTLLSGVFVLFEECVLRIAQPFAIFKIIGYFSSGGTVMSEDELYFWSGILVGAGVIFVLFDQRYWYLSLKTGMQLRIAASAIIYRKALTLSKASLGRSSIGQMVNLLSNDVNRFDNSTVFLQYLWVAPIQFICVMLITSYFAGIEAVAGGALLIFFIFFQTYMGKMFSKLRLMTASKTDIRIQIMNEILDGIKVIKMYAWETPFVQLVTNARKNEMSVIKQTAGCKSFNYAFFSTSSRFVLLPVFLLMVLFGVEINAQKIFLIFGLFDAIKLPLTHFFPSAISASSEALVSMKRIEEFLLLNELRDVQSKINHVILPGRYEPLTVEVTKISGKWLDSSNEFTLKNISFSVEIGELCAIIGPVGCGKSTIIQSLLGEFPVSYGEINIKGRISYASQEAWIFSGSVRQNILMGKPLIDKRYREVIRVCALEHDVREWPDGDYTFVGEKGISLSGGQKSRINLARCVYASADIYILDDPLSAVDPHVGQQLFDTCIQKFLSDKTVILVTHQLQFLKNANNIILLNSDGEIEASGTYEKLMSQSGFVTYLEATSEEEDSDLLSDVLSDVKSDGEYMRSQKKKLKKGVRSRRSSTSLLVPVPYIGGSTERDTFPEEKGSQEWKMGSKETIIEGSVSLSLYHKYFVAGGGFFKFLCVYFLSLITHTLFVVSDWWLRLWTNAADARKLGPLPPTFYFSEYQDMTLQITADFQVDFFNFIYCSIYLSLIIILLFSAQLSVRQLFMSCMRSSQTLHDRMFEKVAFTNSRFFDLNPNGRILNRFSKDIGSIDELLPPVLIDTNWVMSKEYFCIPLYIICFSYSCRPFSLDRVFLFLYL

>Lsa.3522

REWKKETIKKKTSNFYKPSLLKAMLRTFGPQSLLSLIIVLFEECVFRILQPYAVFNIISCFTNPKSSDTELYIWGYVLILSGICFVLFDQRYWYRSLKTGMQIRIAASALIYRKALTLSKRSLGLSSVGQMVNLLSNDVNRFDNSTVFLQYLWVAPLQFTIVLFVTIYHVGSVTILGGFILIFFIFIQTRMINIFSKLRSKTAEKTDSRIQIMSEILNGIKVIKIYAWESSFVQLVNSVRKNEMDVIRRSASYKSFNYAFFSTSSRLVLLPIFFMILIFGGVVNSQNIFLVFGLFETLKLPVTHFFPSAIATSSEAYISLRRIQEFLLLEEWSSRRICHDNQSLKYEPLAIKLNNLGGKWSLASPELTLKDISLNVEVGELIAIIGPVGSGKSTFLQALLGEFPFEYGSMDIVGKLSYASQEAWIFDGTVRQNILMGKPFIEKRYQEVLNACALEHDIQEWSNGDLTFVGGKGISLSGGQKARINLARCVYEKADIYILDDPLSAVDPHVGRHLLEKCIQHFLMNKTVIIATHQIQYIKTADQIVLLNSDGSVETQGRYEELSSSNQGFGSFVKKMDTGNHHKPPSPTKNRKSTISLSSSSISSLNHPMYFSEIIHSDIDEQIDDDIKAKEEFDKNRSLRSESKAEGNVSFKFYLKYFAAANGILGFILIYSLSLITHSLFVFGDWWLRLWTNAADIREQNGTLNDGSFYFTSYQNSSITFIVPELKVDLFNLIYCSIYSGIILVLVLSAQISMRQLFNSCLKSSQNLHDKMFLRVAFTYSSFFDVNPIGRILNRFSKDIGSIDELLPPVVVDTNWAFLSAIGIFFLISSTNPLVLIGVAILLIILITVRRYYLKASRSIKRLEGVTRSPVFSQLASSLDGLTTIRSMKIENMLINEFDDLQDIHTSSYYSFLVVNRFLRSLETFSYLYLRPRLYLYSFSIIEVCFVSHCRYEVITFSLDASGGDVGLSLTLSLALSGMIQWGLRQSAEVENFMTSVERVVEYGELPKEKGLESDIKLDPSWPDKGVVEFSNVSMRYSQHQPNVLKGLNFKTNSFEKIGIIGRTGAGKSSIISALFRLAEPEGEIFIDGLDICKVGLLDIRKKISIIPQDPILFNGSIRKNLDPFNEFTDVQIWNALEQAKLYDIVVDLGHGLDSVVLEFGSNFSVGQRQLFCLARAILRRNKVLIMDEATANVDPFTDSLIQEAIRKEFKDCTVFTIAHRLYTVMDSDRMLVLQNGEIEEFGTPKDLLKDPNNLLSRMVAQSEPNTAHTLRHLASGIIDSIEESQLEENISV

>Lsa.4564

IEAKTDEKEKKMLASSIVDSDIKREFCGRGSLHPWDSSINDFGICFQSLVLVVPTHAILGIISAYYSSYEHGSYYLRPQKAIIAIVSRIIISLGLAAHSALFLLLREKYTENPDGSSLLESFVKIICWTCHAAYNYNLLHRLSLSPRGPNRILFIWILCLIPDLIQARSNLLQPLTIPLIERNLLFYEALFQNIFLATYSLTLFFGPTESESMSYQGPGERDRLWTRAQSYGGFHEEYDYNYLGVAQEDTSIMDRLFFKWVSPLIDKGRMGKLNSSQDVFDLPHNIHTGIVYEDFEFHKSRVRSLQWFRKALSSKFLKEFLCIGMIKFVADVSGFFCPLLLNRLVKFMEDPKADLRWGYFYAFSLFVSTFLVAICNTQFNFKMNELGLKVRASVIQSLYKQTLSVSEANLNKYSRGEIINFMSIDVDRVVNFAPSFHAFWSLPFQMVVTLYLLHQQVGVSSFVGVGFAILMIPINKVIATKIGSLSGHMMSAKDDRVKIIAEIIEGIRVIKYYCWESFFTDKTNSHRNNEIYYLKWRKYLDAVCVYLWASTPVIISVSTFATYSALGNPLTAAKVFTSMALFAMLSGPFNAFPFVINGLIEANVSIKRLARFLSLPSINRIKYFTEYKDENTKASKEIETKIPDIQIMEASFGFTKDCFTLRNIDVSIKRQEFVGVLGPVGSGKTTFLNAILGELEKQSGKISVRDPLSGIAYVQQVPWIQNKSIRDNILFGEMYIHGKYTKVIKACCLDHDFKHLHRGDHTIAGEKGAALSGGQKARIALARAIYQDKDIYLIDDVFSSLDVNVGYKVYTEVMLNLLKRKTRILCTHNPQYINDANIVIKIKDGEFESVKQSTHLVPTSTSPDFSYDTSMFDFKDNSNVIDDSVEEEMQETGVVAYRIYKKYWQAIGTYLAPTILISIFLMQVASNTTDLWLSHWVSTDNFQNNTEDTDHYIYVYSGLAVFHTILTLIRAFLFAYGGIHAAKIIHDLLLGVLLRAKIYFFDSTPAGRILNRFSSDTYAIDDSLPFILNIFLSQIFGVFGRVLVCVYAVPWILIVLLPLGLFYYEIQCKYRPGSRDLKRISSVSLSPIYEHFNETVHGLKIIRASKASQRFLLENEELVECNQKARYAAFSASLWLEIRLQLIGSIVVFSIALISVINFESVDAGLVGLAVSYALGMTSRLAEVVVSFTETEKELVAVERAYDYIDRIYEESYSGVLNMPYNWPNQGVVEFKDIRLRYKDHLPYVLNKVNFKTKPKEKIGIVGRTGAGKSSLIAALFRLSSFSHGEIMVDGIRIRLLPLIDFRRQFAVIPQDPFIFSGTIRQNLDPYAQHSDQELWDSIKLSYLYNIIYSFGSSGLDTLIGDGGKSLSIGQKQLLCLARAIITSAKVVFIDEATASVDKETDRLIRNVLKTAFNDKTVITIAHRIETVLNSDRIFVMSNGQIIEEGKPEDLIKSPNSEFRKLIEQK

>Lsa.14261

QKLKTLLSRDKSSILWECITMNWSFVILGGLFRLFADILGYACALSINIIVNSIAAENENASLHLISNVTINPLKDTRYYDSFFVSELFFDPRVVSIVIFLAALGQGALSQTSNHLLTVSGIRAKNALHVLLYEKSLKLPVGSSNPMQIHRKINLKPLNDEGKGCMGGSCSLDEDYSNEGNIDIGFITNLASEDIINI

>Lsa.14262

KHCTYFDDRKDDDEEEEEEEEESEIFIQRAKRLPETCFTVENGTFAWPKCETNVLQSINLEIKTGSLTIVIGPSGSGKTALISSLIEEMDRITGSVKWNVPDTVALLGQRPWLLNTTIKDNILLGRPFKEKRYKKVIAACDLQTDIDLLPHGDDTEIGERGVLLSGGQRQRLAIARCLYSKSYCTFMDAPFSSLDSKITSHVFEEGVLKILLKRRRTVFL

>Lsa.14263

LISCVANLIGQLIGARARTKLHNDMIQNIMYCPLELFEAFPIGRIINRISHDIFIVDQKIPPCIQRLIMLSFVCIAALAVNSIQSPVFLIFALPMISIYWWLQHFYRRSSRELQRLDSITRAPVLSHFSDTLSGLITVRAFGEQTRFINELCEKVDTNTSAFLILQSGCRWLGVYLDAAGAIFVFLSILVNLFFPRKGREVTSSASIGLSVNYSLLVPIYLAWVVKFAANIENYMNAVERVLEYTHFPSEEEDFSEFQAVHASSMNSSRRSFLLRGRGKHDEIEEDVRVSGDFLQNDSEGLIIRFNSVCLAPSFEYRRLPGIQQGFSLEIPYRQKVGICGRSGSGKSTLLMGIVRLSRVLQGSITINGININAI

>Lsa.14264

RKFVITIPQDAVLFSGTIRSNLDPENDFSDELIWSTLDKADCGKTVRNFPDGLDTCVTENGDNFSLGQRQELNIVKALLRRPRVVILDESTSALDPNREVALHNTLLEAFEDSTLISVAHRLSNIVEYERVLVMGEGRILEDGNPKELLKKPMGFFSALWRAAGEKPLS

>Lsa.10176

KRLLLIMEIKDNDGSKSYGFNLQFLHRFWHLQSLIFTDRINIGLAIIILLICSLEQLVMYKIGMIPGQFIRALVSEDESKFVRAIVLSLIIVCFMTLVLSARILTSELLSVSWRRSLTRCLHSLYFFNTGFYTLKLDNPDQRIASDAECLVSIYGQILSEILLSPFVISYYIYDSYRGRGSGPYGPLIIFGFFLFGTFINKTLLTPVVNSGVEVRSKEGDFRFKHAEIRSQSESLAFLGQMGSFAEASRVDHLLEILCEAQRKLVLATYRLNLATNFFDYGASIISYLIVGIPVYNGFYKGLNVEELSGIISETAFVNIMLISKFSRLVDLAGKVSRLASVTHRVAEFVEKLSQEKIKDLKDFDNHAESSPLLMNDSDDSSVSDETVLTLSNLSIGLPDISRKTLVSNLNLELKIGDNLLITGSSSCGKTSLLRVLRGLWNEKSGSYKFGRKTVI

>Lsa.5856

KLFDNDSHPYCLISSTQLKKGQSDRFIIMISNLNNSHFPFIIMISNLNNSHFPPLTRNYKVNWEFIKRIFTIHKIIFSKWTSVLIFILLIAVCGLEQFLAYNTGLIAGNFYEVLLNKDMKGFQMQVLISMGFIMGIAVAMTFRMYVSNILSLFFRAILTRRLHGSYFNENEFYRINVILRELHIDNPDQRMTADIDIFCRAYGDMMSRLITSPFVIAYYSWDAYTRAGWTGPVGVYVFFLLGTFINILLMSPLSKKVFNLERREGDFRYKHVFVRDHSEEMAIMSASKREYKSTNDKLKKLVVAQQSLYNREAFLDLSTNLFNYMGAILSYLIIAVPIFMGYYDYMDKATLGKQISQNSFVCMTLVYNFTQLIDLATKVANMSGVTHRIVELLEVMDEEPEESSYQDCLEYRTHVLQSRGGEEEVVLINNVTLTPPFSDRILIENLDLKILKGQRLLITGSSSSGKTSILRHIRGLWKARIGRTVVNAKKVYYLPQNPIFTDGSLIENMIYPSERDPTLDDSWFIEQLIALELNHLLERYSLRADRSHWVDILSPGEMQRICFIRIFYHAPDLIILDEATSSLPTCAEGVIYEKLDQTFSDATIISVGHRESLREFHDLELQLTNGRTWSLIDLNQSRIEEEEKQRSLSSCSSPDT

>Lsa.1035

LCERIARFHGTLDIYAKEKKRLVPLICLNKKKKILNKKLPYIYLFLFLFTCNYLGMPPKTNQRKKKDSSVNSELTRIAIVNADRCKPKRCRQECKKSCPVVRMGKLCIEVSPNSKIATISEELCIGCGICVKKCPFEAVVIINLPSNLETETTHRYSANSFKLHRLPVPRPGVVLGLVGTNGIGKSTALKILAGKQKPNLGLFNEPPDWSDILHYFRGSELQNYFTKILEDNLKAVIKPQYVDQIPKAVKGSVQSLLDKKDEMNNQNSVANLLDLSPQVRQRKVDELSGGELQRFACAMVCIQKADIFMFDEPSSYLDVKQRINASQAIRNLMHPSKYIIVVEHDLAVLDYLSDFICCLYGVPGAYGVVTLPSGVREGINIFLDGFIPTENLRFRQENLIFKVSENATEEEVKRMSSYNYPTMSKTMGTFKLSVQAGNFSDSEILVMLGENGTGKTTFIRMMAGKLEPDTGSGEIPQLNISYKPQKISPKSQGTVRTLLHEKIRDAYIHPQFVADVMRPMKIDDIIDQEVQNLSGGELQRVALTLCLGKPADVYLIDEPSAYLDSEQRLVAAKVIKRFILHAKKTGFVVEHDFIMATYLADRVIVFEGLPSIDTRANCPQSLLTGMNKFLSQLAITFRRDPNNYRPRINKMNSQNDSMQKAAGNFFFLED

>Lsa.20458

ARKQLGSFGLQSHAHTIKMKDLSGGQKSRVALAELTLSAPDVVILDEPTNNLDIESIDALGDAIREYKGGVIIVTHDERLIRDTECQLWVVEEQTINEIEGDFDDYRKE

>Lsa.9678

YIQIGIQVTTREFLGRRIFGVKMPSDYQKKKLAKKKEAAKIKGGKKATNDENSVSKDTNDGTSSYMKDMLSNGTKKEMTNEEELCYRLENEAKLAAEARACTGVLGIHPMSRDIKIDNFSVTFHGAELLTDTKLELSCGQRYGLIGDNGSGKSSLLAVLGNREVPLQDHIDIYYLSREMPASEKSAIQAVMEADQERIKLEHLAEKLAHLDDEEVHEYLMEVYERLEEIGSDTAEAKASALLKGLGFDKEMQAKACKDYSGGWRMRIALARALFIKPHLLLLDEPTNHLDLEACVWLEEELRKYNRILVLISHSQDFMNGVCTNIMHLDGKKLKYYGGNYDAFIRTRVELLENQAKRYQWEQDQIAHMKNYIARFGHGSAKLARQAQSKEKTLAKMVASGLTEKVATERNFSFYFFSCGKIPPPVIMVQNVSFRYNDSSPWIYRNLEFGMDLDTRLALVGPNGAGKSTLLKLIYGDLIPSEGMIRRNNHLKIGRYHQHLHELLEMDSHQHLHELLEMDSTPLEYMMKQFPAVKERDEMRKIIGRYGITGKMQTSPIKQLSDGQRCRVCFAWLAWQSPHMLLLDEPTNHLDMETIDALGEAINNFEGGLVLVSHDFRLINQVAEEIWVCEHQKVTKWDSDILKYKEHLKNKVLKELSKDRA

>Lsa.8082

VTPNISRTHCIKSQVLNPGSVVMSEGEGGCGFLSAELRRLCLRRGACSLDSQSEVLEYCEGVLWRCGEDLESGSEVYDALGELLEELLPGEAEAEIRALCDELYGLLGLRTGKSRPGEGVLCLESGGSGVSELCSPICLGESLEGAEVLDSLQGGRGSIWMEEKRGLGRVDKEKLEKAEKALLKKQGKDSSLKSHSSSQLILQATASQVLPKTRTDFNLSKDIRLEGVDVAFGDKILIQNTNLSLIHGRRYGLVGRNGLGKSTFLRMLSSSQLRIPTHISILHVEQEVVGDDTSALQSVLESDTKRQSLIQEAQHLSKEALNSYRLSEIYTEMEAIEADKAPSRASIILSGLGFDIIMQNKSTKKFSGGWRMRIALARALFCKPDLLLLDEPTNMLDMEAIVWLEKYLQSWQSTLLVVSHNRNFLDNVTTDIIHLQSKRLDTYKGNYTVFINQMTEKLKAQRREYEAQQDYRKHVQEFIDKFRFNAKRASLVQSRIKQLEKLPFLMPVVKEAEIIIRFPDVQKLSPPILCLNDVKFSYDGKNDIFDHVDISATMESRICIVGKNGSGKSTLLKLIMEEISVTDGRRIVHRNLKFGYFSQHHVDQLNMNLCPLQIMEKKFRGKKMEECRQMLGHFGISGDLALQKTSSLSGGQKSRVAFAVLCGEEPNFLILDEPTNHLDLETIDALGHGLMKYNGGLILVCHDERLIRMVCQELWVCSKGKISRLEGGFDEYRRILELELEI

>Lsa.2606

GRSKAMITNEYVLELTNVFHSGQVETGTCMQKMIGTVRTGVILKDVSMELHGGELSAVLGSKGSGKHALLEVISRRAQGPTRGQILLNGVPMSMRLFQESCGYVTQKCDLLPGITVKETLEYSANLTVGSKVGSFVKHSRVKQVMADLALTNLANRNSESLSQSEYRRLVIGTQLVRDPVVLLLDEPTWDLDPLNTYFIVSILANHAKKYNRIVLLTMEKPRSDIFPFLDRVTYLCLGDVVYTGATRMMLDYFRSIGFPCPELENPLMYYLCLSTVDRRSRDRFIESNNQIASLVEKFKMEGGPYRKYGGPPPDAESVLDAASHQKVPLTAYGRPNSITIFYYLLMRSWCRISPFNIHGMQQFFIKILMMPTFFFLLWIFYYNSSSKGMENQYQRNFVTRNGLVFNSLAGAYFMSILATVTSFATDRTRYYQEVREGIYGGPLFLLSNLVQSLPLSALTTFMSTFIIFRGLKNELICYPDGDSNICKSYSSFDSDLDDLNYHLEYSYYPDLITHWLALWACYLLAEQQTVSILMVVKSSYTATLVSVFLTVLYLVLGSATVRSYSSLPKTFYHLTYLTQSRYTGLVLNDIEFFNKTSLQNLGLYDDINERVNPCKGSRLGFGCRYGNGTLFLTEKYGYKHDRLETIMDRWFNIGVSILFPAILFLLNNVLYLIPLPAFVKAKFRE

>Lsa.25615

KGLYRGQLCNRHSIDFLCINPKAMWEMKAMDGRRYNKTGSSMMSEVQTGGGGGGGHTLPHGQASTSEDLHAWSIFRQNLNSDFTDSALGSSEKSPMPYGNFHLRESTMHSILSNPKYGPKSELGANMYTYLKFGLPRVLPPLHKRENSSGYDSTDEETHHHTSKNRSNGVLRSARSEDFLNYGREEIQFATNYKRNRDRHPQSAINHRRLGTSSNSTTDRRYKSASEANLLSPTSYYYHQDDLSGTKTGRLRSLARSRASSTNRISSEKDLRDGGAFVNRGMEMDEDDDEEEEIDTELDKDSVMANNNKLGGNKAASTLSILSKSKINGVSYRNEAPMVNDKYFPKDRIYGSNGNFLGGGLHGADIIREGTKYPHLQIRGLNFEIRRYGHFIRLLDDISLDVKGGELLSIMATKEDEGTALCDIIANSFNHWNTRLDTDIILNGISVNTKRLEDRVSYVKRNINFSPDMSVRQTMLFHSFLREPGTHTRNNDTKGRINALIEDLGLVQVKHTRVKDLTVSERQRLNVAAHLLMDTDIVVLDQPTRGMDIFDTFFLVEYLRQWAGRGRIVIITLHPPTYEILTMISKILLISTGRSMYYGKRREMLPYFALIEYPCPAFKNPSDYYLDLVTLDDLSSEAMLESSQRIDQLASTFKRRMEPLPDPGPPGVLPSKIKRANFLDQIFGLWIRALIYMYPFNVIEWVKMVLLSGGISILVGVIFLGIRWQYWDREWQENPTFDQDNINDRLGFHHVMMSVGIWPMMMAMITNEWANKMPISRDVDDKLYSKMAYIFIKTLYSIPGIVGIFLAYIIPGYLLAGIHYQNVNDLDVFYYYIGYMMLYLLSIRMMIMCFVHLSSSRHWASAMGGTILVILSLVNGYVIHVKDLGDWTSWIKYVSPQYWMNHPIQRGEFSPISIFHCKDNPVITENSIIKQVPCGLSSGNKTLDYFQFGDKFQNIVRAPWYIFMPIFLTLFFYAFWQILCYVFYLGRTQKARQSRSRKSKV

>Lsa.12984

YDTSLVVKLSFNKLFLLISTFVENGTYMFQWSFYGQVSVLMTDRTSFKTGFDRMNKPVNPICEDDDSSFKNMENEPRIVVALRDACKYYGKNAMENRVLNNLNLTITEGSIYGLLGASGCGKTTVLSCIVGRKKLNKGQVTVFGGSPGDKGIGIPGNRIGYMPQEIALYKEFTVRETLKYFGRLYEMETDQIDQRIDFILKFLQITRDRDMVGKLSGGQKRRVSFAAALLHDPELYILDEPTVGVDPRLRKSIWNHLTDLALNRKKTILITTHYIEEARQATSIGLMRNGKLLAESSPEKLLQIYGEPSLEDVFLTLCVSQDELNSIESFNYSTSFSNSLKKVVEGIFGIFRKKPPESIMISSTKISNANEDGHTSKGVYPVSYNKSSESIDSYSSTKPTNIDPKLLSNFSKNKFGNHRILSPKKLKALIMKNFIQMWRNIPNLLFIFLLPAIEVLLFCIAIGNDPTNLNFGIVNNEFPYASNASNYSCSTLQGCEFENLSCRFLSNLTNRKDLNFKYFEDEQLAKTEVLNGEIWGYMTISSNFSEAFLDRLWNTLNVDSESLLQSSLRVYLDMTNQQVSFSIKRIIFDSYKDFIGGLMTDCELPSELAASPVRYEDPIYGVENPSFTSFMAPGIIVIIIYFLAMALTGDAFLLERRDGLLDRSWVAGVSALEYILAVILTQFIVMIIQTIITLIFILIVFQITCNGPLLWLIVLTLLQGTAGMTFGFMNSAIFTDQSTAMQVALASFFPNLLLSGIIWPLEGMPYYLKTFSQILPSTAACQAMRDIMSRGWGVAYTSVYSGILISMSWITVFVLISVITIRLTV

>Lsa.14023

LLKMTDFEGSEMDQLQIMKNDNEHDPRAAISVKGAYKSFGWGKKKVNVLVNLSIRITKGHIYGLLGPSGSGKTTLLQCVIGKQSLDSGSILVFGEYPGTKDLGVPGKRVGYMPQDLAMYMELTIMETLEFYGRIFNMPKAKIKKRAKFLVELLELPKKKILIQKLSGGHQRRASLAVALLHEPDLLILDEPTVGVDPVLRRNIWGHLVDICNNPIRKTTIVVTTHYVDEARQANMVGMMRFGRILAQNCPSRLLKIYNKPTLEAVFFNLCVRDESEISIYSCETCESDEVLNLKVSDFRENYSKVTIPYPSKKNCPRLSSLLAFHRLKALVLKNFVRMWRNITFLLFQFIIPTIQVTLFCIAIGRDIKGLSMAVVNDDIVGKECNGYAKECYHQSIPFISFAGNDQPKNISCLFLQKVDEAIIDLVHYKEYKTAKDAVIAGKHWGVIHFLNGFSDAFRWRLGALFQKEDVDIKKINTSYIYIDLDTTNQQVMYSLKRSLYETFRSFSKELLTECYDIKNVEGYPAKYEKPIYGSINPTFTEFMAPGIILSIAFFMAVGLTAQSFILERNDGLLERAWVAGVTSAENMLAYMIAQFVVVIFQVTFAIVFLIYVFQIPSVGSLWLLGLLTTFQGVCGMSLGLLISALCNHEQDAIQVALGLFYPTLLLSGIVWPIEGMPSSLKYVSYIFPQTIACEAMRGILSRGWDPRWKVVFLGFVATTIWIFIFQILGALILRFKR

>Lsa.21408

NKTNICKKYKFSHRIFLLYLFNMEYSPIVVLEKVRFSYENGVEILRNVDLRIDKGCVTALLGPSASGKTTILSSIVGIIKPSSGQVIRHYNSLGFMPQSYCLHEYFTVREIFKFYAMLYDIINPDTIINKIIRTMDLPPDRIIQNCSGGMKRRISLCCAVMHSPDLIILDEPSVGVDPLLRRRMWDFLLDLAKNGSAIVVSTHYAEEASLAHKFAFLRKGRILQSGNVNELGDMGLNYYEQCLEDERRFRSDDPKPFNNKIEKLKNVILRPKLEFLDRNPCNSRRAFSSMFYKNLIVLKRHKLFMMFQAILPLLTVFIFTLSIGRSLKDIHITVLNEDQSDCSKQNVNFICPDIASIISPNLDFFAYSSCKIIDNLTLKSLKIQFHEKTMSNAKNRIMNGESVAIIHFPKGFSESLTLKLKNSINSIKETHKNSSHLDSIGIILDTTNKLVVDQVYSSLHQALEITIDKFFKECEISLNSSSILMNIDFSTDEKEDYDLTNGMMPVMANAILYFMAFALTADLTAGEKEAGLLVRISLTGVKFKSILISQLLVQSIVVLIHIFSCLLFLKISVCPQCSLSLYLLIGFLYSLQSLSGMTIGILLSTICSGRDQVIQYGLAIIFPTFLLCGLLWPLTSLPLYLKALSYSLPVTLSGQLIRRIIYRQCLDNMLYLFAVSLIPLIWSGGCIGLTLFVASRSGSLYRL

>Lsa.23267

WIRNAYKHFGTKSNTSLFTGLNMSVPKGVIYGLLGPSGCGKTTLLRVITGREKLNSGEFKIFGEDPGTVGLEVPGRRIGYMPQDLALYMELTILETLFFYGRLNKMQTSKIRSRAETLIEILELPHSGRLVQNMSGGQRRRISLAVALLHEPELLVLDEPTVGVDPVLRQYIWDHLLTIVNNSSKKRTIIITTHYVEEARQAHIVGMLRYGKILDEDAPTKLLEKFKALTLEEVFYELCQQEQRVICEGSEDEEESFLMSRNQRNSSLSSDSKFRRRDAFSISIEAPKKHFFPQMSSFFAIHRWKALVIKNFLRMFRKIGFLIFQLIFPAIQASAFCIAIGKDIKGMTVAVVNEEATYQECQNNYAGCILSFQSSKYVTMSKTSDLQDNLSCRLLYYIERGELDIKYYDDFETARNKVLNGDHWGVIHFEKGFSKKLAEEMHFIVSEPYAKDKEINTRMHIYLDMTNQQISLSIKQSLHSSADEFLKESLRACNKSEELGNSLLYFGNTIYGSNKPSYTEFMAPGIILSIAFFMAVGLTSQSFVTERREGLLERSFATGVTTLEVMLAHMVAQFIIMILQVTFVLLFMILVFKIPANGSIVLMISLVILQGLCGMSFGLLISSFCSTEHDAIQLALGSFYPLLLLSGIIWPLEGMSKNLRYFSYTLPQTLACKAMRGILSRGWNLEWSIVYLGFIVTIAWIFIFQILSLLLFRIRK

>Lsa.23269

TLVENLIIIIIIMNEVDDVGVPESEEVLPSLLEQKEDGFQNLQPAIWIRDVYKQYGWGRKKVNVLMDLDMTIPKGVIYGLLGPSGCGKTTLLQCTIGRQRIQSGELLVFGHKPGSSGSGVPGRRVGYMPQELALYKEFTILETLEYFGRIFHMSPGSIKMRAEFLLDFLDLPRKNRLIQNLSGGQQRRASLAIALLHEPELLILDEPTVGVDPVLRHSIWEHLLAISNNPSKKTTIVITTHYVEEARRAHIVGMMRYGRLLAESPPANLMKLYNKPTLELVFFNLCRKESSDDSLFAEHSTPRTPKHNPDVISLKQIQTSSEAVAPSFSEQKSCLPRMDSIFALHRLISLVIKNFIRMWRNIGFLIFQFILPTLQVSLFCLAIGGDLKGMSLAVANEDIGSKTCTGFAEGCSISENPLDYLSFSDPVIHPAFNLSCRYLSFMDKDAVDLVYYDDYASAKQAVELGKHWGMLHFTPRFSKAFPDRVIKLISMEVPSNQTLFDSQVHAHLDMTNQQVGHTLKMIMTLSFQSFVEDILQSCNKSQKILGFPLHFHEPIYGAMEPKFTEFMAPGVILSITYFMAGGVILSITYFMAVGLTAQSFILERKEGLLERSWVAGVTATEVMLAHIIAQFAVMVVQVGFVLLFMIYVFSIPSQGPLFLIILLTILQGICGMSFGLVISSMCNTEQDAIQVALGSFYPILLLSGIIWPLEGMPRELKYVSYALPQTLACEAMRGVLSRGWNLEWTQVSQGFLVTIAWICVFQIVSAIILRIRR
