# Supplementary material for: A Survey of the ATP-Binding Cassette (ABC) Gene Superfamily in the Salmon Louse (Lepeophtheirus salmonis)
Source: PLoS One. 2015 Sep 29;10(9):e0137394. doi: 10.1371/journal.pone.0137394 (PMC4587908; doi:10.1371/journal.pone.0137394)
Supplement: S1 Table — (DOC) [file pone.0137394.s011.doc]

Table S1. Samples of *L. salmonis* stages used to prepare a multi-stage mRNA library used in high-throughpout RNA sequencing (RNA-seq) on a Illumina HiSeq 2000 platform.

| Sample_ID | Salmon_louse_stage |
| --- | --- |
| 1 | Egg strings (light colouration) |
| 2 | Egg strings (dark colouration) |
| 3 | Nauplius (24 h growth at 8°C) |
| 4 | Nauplius (24 h growth at 10.5°C) |
| 5 | Nauplius (48 h growth at 8°C) |
| 6 | Nauplius (48 h growth at 10.5°C) |
| 7 | Free-living copepodid |
| 8 | Attached copepod (24 h dpi) |
| 9 | Attached copepod (48 h dpi) |
| 10 | Chalimus I (72 h dpi) |
| 11 | Chalimus I (96 h dpi) |
| 12 | Chalimus II |
| 13 | Chalimus III |
| 14 | Chalimus IV |
| 15 | Preadult I male |
| 16 | Preadult II male |
| 17 | Adult male |
| 18 | Preadult I female |
| 19 | Preadult II female |
| 20 | Adult virgin female |
| 21 | Adult gravid female |
