# Supplementary material for: A Survey of the ATP-Binding Cassette (ABC) Gene Superfamily in the Salmon Louse (Lepeophtheirus salmonis)
Source: PLoS One. 2015 Sep 29;10(9):e0137394. doi: 10.1371/journal.pone.0137394 (PMC4587908; doi:10.1371/journal.pone.0137394)
Supplement: S2 Table — (DOC) [file pone.0137394.s012.doc]

Table S2. ABC transporter-related hidden Markov models used to identify ABC superfamily in a L. salmonis reference transcriptome.

| Motif_ID | Description |
| --- | --- |
| PF00005 | ABC transporter |
| PF00664 | ABC transporter transmembrane region |
| PF06472 | ABC transporter transmembrane region 2 |
| PPTHR11384 | ATP-binding cassette |
| PTHR19211 | ATP-binding transport protein-related |
| PPTHR19229 | ATP-binding cassette transporter subfamily A |
| PF12698 | ABC-2 family transporter protein |
| PR01868 | ABC transporter family E signature |
| PS50893 | ATP-binding cassette |
| PS51012 | ABC transporter integral membrane type-2 domain |
| SM00382 | ATPases associated with a variety of cellular activities |
| SSF90123 | ABC transporter |
