# Supplementary material for: A Survey of the ATP-Binding Cassette (ABC) Gene Superfamily in the Salmon Louse (Lepeophtheirus salmonis)
Source: PLoS One. 2015 Sep 29;10(9):e0137394. doi: 10.1371/journal.pone.0137394 (PMC4587908; doi:10.1371/journal.pone.0137394)
Supplement: S4 Table — (DOC) [file pone.0137394.s014.doc]

Table S3. Accession numbers of sequences of ABC superfamily members in metazoan genomes.

| ABC subfamily | *Daphnia pulex* | *C.elegans* | | *D. melanogaster* | *H. sapiens* | | *T. urticae* | | *T. castaneum* | | | *L. salmonis* | | |
| --- | --- | --- | --- | --- | --- | --- | --- | --- | --- | --- | --- | --- | --- | --- |
| A | Dappu1_346971 EFX89859.1 | C24F3.5 | CAA18775 | CG34120 NP_001036287.1 | ABCA1 | NP_005493.2 | TuABCA-01 | tetur01g00580 | TcABCA-UA | GLEAN_10722 | XP_967122.2 | Lsa.1758 | HACA01002058 | |
|  | Dappu1_312055 EFX87570 | C48B4.4 | NP_499115 | CG1801 AAF50836 | ABCA2 | NP_001597.2 | TuABCA-02 | tetur01g15090 | TcABCA-UB | GLEAN_2280 | XP_970623.2 | Lsa.14583 | HACA01016690 | |
|  | Dappu1_312056 EFX87571.1 | F12B6.1 | AAB54153 | CG8908 AAF57490 | ABCA3 | NP_001080.2 | TuABCA-03 | tetur11g05030 | TcABCA-UC | GLEAN_2278 | XP_001809119.1 | Lsa.1680 | HACA01001963 | |
|  | Dappu1_347506 EFX70287.1 | F55G11.9 | CAB05222 | CG42816 NP_730301.3 | ABCA4 | NP_000341.2 | TuABCA-04 | tetur11g05040 | TcABCA-UD |  | XP_970754.2 |  |  |  |
|  |  | F56F4.6 | AAB54203 | CG31213 NP_001262752.1 | ABCA5 | NP_758424.1 | TuABCA-05 | tetur11g05200 | TcABCA-UE | GLEAN_2274 | XP_970882.2 |  |  |  |
|  |  | Y39D8C.1 | AAC69223 | CG6052 AAF49312 | ABCA6 | XP_006721853.1 | TuABCA-06 | tetur15g01990 | TcABCA-3A | GLEAN_10527 | XP_967691.2 |  |  |  |
|  |  | Y53C10A.9 | CAA22142 | CG1718 AAF50837 | ABCA7 | NP_061985.2 | TuABCA-07 | tetur25g01640 | TcABCA-6A | GLEAN_15859 | XP_001812136.1 |  |  |  |
|  |  |  |  | CG31731 ABI31314.1 | ABCA8 | NP_001275914.1 | TuABCA-08 | tetur27g01890 | TcABCA-7A |  | XP_969271.1 |  |  |  |
|  |  |  |  | CG43672 AAO41659.2 | ABCA9 | NP_525022.2 | TuABCA-09 | tetur30g01960 | TcABCA-9A | GLEAN_16282 | GLEAN_16282 |  |  |  |
|  |  |  |  | CG1494 AAF50838 | ABCA10 | NP_525021.3 |  |  | TcABCA-9B | GLEAN_16281 | GLEAN_16281 |  |  |  |
|  |  |  |  |  | ABCA12 | NP_775099.2 |  |  |  |  |  |  |  |  |
|  |  |  |  |  | ABCA13 | NP_689914.3 |  |  |  |  |  |  |  |  |
|  |  |  |  |  |  |  |  |  |  |  |  |  |  |  |
|  |  |  |  |  |  |  |  |  |  |  |  |  |  |  |
|  |  |  |  |  |  |  |  |  |  |  |  |  |  |  |
| B-half | Dappu1_347270 EFX85894.1 | Haf-1 | CAB02812 | CG1824 AAF48177 | ABCB2 | Q03518 | TuABCB-03 | tetur17g02000 | TcABCB-4A | GLEAN_8383 | XP_966724.1 | Lsa.26127 | HACA01029620 | |
|  | Dappu1_347266 EFX81018.1 | Haf-2 | AAC71121 | CG3156 NP_569844 | ABCB3 | Q03519 | TuABCB-04 | tetur32g01330 | TcABCB-5A | GLEAN_14809 | XP_001813375.1 | Lsa.7262 | HACA01008343 | |
|  | Dappu1_347268 EFX79468.1 | Haf-3 | CAB09418 | CG4225 AAF55241 | ABCB6 | Q9NP58 |  |  | TcABCB-6A | GLEAN_15192 | XP_974441.2 | Lsa.643 | HACA01000740 | |
|  | Dappu1_347275 EFX70521.1 | Haf-4 | AAC68724 | CG7955 AAF47525 | ABCB7 | O75027 |  |  | TcABCB-7A | GLEAN_9730 | XP_972133.1 |  |  |  |
|  | Dappu1_347276 EFX65703.1 | Haf-5 | CAB04947 |  | ABCB8 | Q9NUT2 |  |  |  |  |  |  |  |  |
|  | Dappu1_68106, EFX62242 | Haf-6 | AAK29911 |  | ABCB9 | Q9NP78 |  |  |  |  |  |  |  |  |
|  |  | Haf-7 | CAB60586 |  | ABCB10 | Q9NRK6 |  |  |  |  |  |  |  |  |
|  |  | Haf-8 | CAB16503 |  |  |  |  |  |  |  |  |  |  |  |
|  |  | Haf-9 | AAK39394 |  |  |  |  |  |  |  |  |  |  |  |
|  |  | Abtm-1 | CCD73540.1 |  |  |  |  |  |  |  |  |  |  |  |
|  |  |  |  |  |  |  |  |  |  |  |  |  |  |  |
|  |  |  |  |  |  |  |  |  |  |  |  |  |  |  |
|  |  |  |  |  |  |  |  |  |  |  |  |  |  |  |
|  |  |  |  |  |  |  |  |  |  |  |  |  |  |  |
|  |  |  |  |  |  |  |  |  |  |  |  |  |  |  |
| B – full | Dappu1_347264 EFX85237.1 | Pgp-1 | CAB01232 | CG10226 AAF50670 | ABCB1/MDR1 | 4505769 | TuABCB-01 | tetur11g04030 | TcABCB-3A | GLEAN_03402 | XP_967244.2 | Lsa.4043 | HACA01004649 | |
|  | Dappu1_347265 EFX86431.1 | Pgp-2 | AAB52482 | Mdr49 NP_523724 | ABCB4/MDR3 | AAA36207 | TuABCB-02 | tetur11g04040 | TcABCB-3B | GLEAN_03797 | XP_001810982.1 |  |  |  |
|  |  | Pgp-3 | CAA91467 | Mdr50 NP_523740 | ABCB5 | AAO73470 |  |  |  |  |  |  |  |  |
|  |  | Pgp-4 | CAA91463 | Mdr65 NP_476831 | ABCB11/BSEP | AF091582 |  |  |  |  |  |  |  |  |
|  |  | Pgp-5 | CAA94202 |  |  |  |  |  |  |  |  |  |  |  |
|  |  | Pgp-6 | CAA94220 |  |  |  |  |  |  |  |  |  |  |  |
|  |  | Pgp-7 | CAA94219 |  |  |  |  |  |  |  |  |  |  |  |
|  |  | Pgp-8 | CAA94203 |  |  |  |  |  |  |  |  |  |  |  |
|  |  | Pgp-9 | CAB03973 |  |  |  |  |  |  |  |  |  |  |  |
|  |  | Pgp-10 | AAC48149 |  |  |  |  |  |  |  |  |  |  |  |
|  |  | Pgp-11 | CAA88940 |  |  |  |  |  |  |  |  |  |  |  |
|  |  | Pgp-12 | CAA91799 |  |  |  |  |  |  |  |  |  |  |  |
|  |  | Pgp-13 | CAA91800 |  |  |  |  |  |  |  |  |  |  |  |
|  |  | Pgp-14 | CAA91801 |  |  |  |  |  |  |  |  |  |  |  |
|  |  |  |  |  |  |  |  |  |  |  |  |  |  |  |
| C | Dappu1_347281 EFX72783.1 | Cft-1 | AAK52175 | CG10505 AAF46706 | ABCC1/MRP1 | AAB46616 | TuABCC-01 | tetur01g07880 | TcABCC-UA | 8570 | XP_971058.2 | Lsa.11278 | HACA01012937 | |
|  | Dappu1_347295 EFX72657.1 | Mrp-1 | AAD31550 | CG11897 AAF56869 | ABCC2/MRP2 | CAA65259 | TuABCC-02 | tetur01g10390 | TcABCC-UB | 1803 | XP_970354.2 | Lsa.22810 | HACA01025969 | |
|  | Dappu1_347292 EFX72656.1 | Mrp-2 | AAB07022 | CG11898 AAF56870 | ABCC3/MRP3 | AB010887 | TuABCC-03 | tetur01g15310 | TcABCC-4A | 8035 | XP_972214.2 | Lsa.29272* | HACA01033013 HACA01033014 | |
|  | Dappu1_347548 EFX63846.1 | Mrp-3 | CAA92148 | CG14709 AAF54656 | ABCC4/MRP4 | NP_005836 | TuABCC-04 | tetur01g15330 | TcABCC-5A | 13752 | XP_973658.1 | Lsa.6310 | HACA01007250 | |
|  | Dappu1_347323 EFX82733.1 | Mrp-4 | CAB02667 | CG31792 NP_724148 | ABCC5/MRP5 | AAB71758 | TuABCC-05 | tetur01g15340 | TcABCC-5B | 13751 | XP_973693.1 | Lsa.8882* | HACA01010189 HACA01010190 | |
|  | Dappu1_442500 EFX68442.1 | Mrp-5 | CAB54225 | CG31793 NP_609930 | ABCC6/MRP6 | AF076622 | TuABCC-06 | tetur03g02240 | TcABCC-5C | 13750 | XP_973725.2 | Lsa.23107* | HACA01026296 HACA01026297 | |
|  | Dappu1_347288 EFX68457.1 | Mrp-6 | AAA82317 | CG4562 AAF55707 | ABCC7/CFTR | AAC13657 | TuABCC-07 | tetur03g07460 | TcABCC-5D | 13749 | XP_973757.2 | Lsa.3521* | HACA01004060 HACA01004061 | |
|  | Dappu1_328283 EFX70472.1 | Mrp-7 | CAA21622 | CG5789 AAF56312 | ABCC8/SUR1 | AAB02278 | TuABCC-08 | tetur03g07490 | TcABCC-5E | 14089 | XP_968524.2 | Lsa.3522 | HACA01004062 | |
|  |  | Mrp-8 | CAA22110 | CG6214 AAF53223 | ABCC9/SUR2 | AF061323 | TuABCC-09 | tetur03g07840 | TcABCC-5F | 14090 | XP_968603.2 | Lsa.4564 | HACA01005249 | |
|  |  |  |  | CG7627 AAF52648 | ABCC10/MRP7 | NP_258261 | TuABCC-10 | tetur03g09800 | TcABCC-5G | 14091 | N/A | Lsa.14261 | HACA01016334 | |
|  |  |  |  | CG7806 AAF52639 | ABCC11/MRP8 | NP_149163 | TuABCC-11 | tetur03g09880 | TcABCC-5H | 14092 | XP_968748.1 | Lsa.14262 | HACA01016335 | |
|  |  |  |  | CG8799 AAF58947 | ABCC12/MRP9 | NM_033226 | TuABCC-12 | tetur04g04360 | TcABCC-5I | 10434 | N/A | Lsa.14263 | HACA01016336 | |
|  |  |  |  | CG9270 AAF53950 |  |  | TuABCC-13 | tetur04g05540 | TcABCC-5J | 14379 | XP_971732.1 | Lsa.14264 | HACA01016337 | |
|  |  |  |  | Sur NP_477472 |  |  | TuABCC-14 | tetur04g07860 | TcABCC-5K | 14380 | XP_971687.2 |  |  |  |
|  |  |  |  |  |  |  | TuABCC-15 | tetur04g07910 | TcABCC-5L | 14381 | N/A |  |  |  |
|  |  |  |  |  |  |  | TuABCC-16 | tetur05g01110 | TcABCC-5M | 14382 | N/A |  |  |  |
|  |  |  |  |  |  |  | TuABCC-17 | tetur05g04300 | TcABCC-5N | 14383 | XP_971802.2 |  |  |  |
|  |  |  |  |  |  |  | TuABCC-18 | tetur06g00360 | TcABCC-5O | 14384 | XP_971857.2 |  |  |  |
|  |  |  |  |  |  |  | TuABCC-19 | tetur06g03510 | TcABCC-5P | 14385 | XP_971908.1 |  |  |  |
|  |  |  |  |  |  |  | TuABCC-20 | tetur06g03560 | TcABCC-5Q | 14386 | XP_971965.2 |  |  |  |
|  |  |  |  |  |  |  | TuABCC-21 | tetur07g04290 | TcABCC-5R | 14403 | N/A |  |  |  |
|  |  |  |  |  |  |  | TuABCC-22 | tetur07g04410 | TcABCC-5S | 14589 | XP_970316.2 |  |  |  |
|  |  |  |  |  |  |  | TuABCC-23 | tetur09g00580 | TcABCC-5T | 14775 | XP_969781.1 |  |  |  |
|  |  |  |  |  |  |  | TuABCC-24 | tetur09g00590 | TcABCC-5U |  | XP_969849.1 |  |  |  |
|  |  |  |  |  |  |  | TuABCC-25 | tetur09g04610 | TcABCC-5V |  | XP_001810350.1 |  |  |  |
|  |  |  |  |  |  |  | TuABCC-26 | tetur09g04620 | TcABCC-6A | 15346 | XP_969711.1 |  |  |  |
|  |  |  |  |  |  |  | TuABCC-27 | tetur11g02060 | TcABCC-6B | 15131 | N/A |  |  |  |
|  |  |  |  |  |  |  | TuABCC-28 | tetur11g02120 | TcABCC-6C | 14880 | XP_970526.1 |  |  |  |
|  |  |  |  |  |  |  | TuABCC-29 | tetur11g05990 | TcABCC-7A | 9891 | XP_972486.2 |  |  |  |
|  |  |  |  |  |  |  | TuABCC-30 | tetur14g02290 | TcABCC-7B | 9892 | XP_972534.1 |  |  |  |
|  |  |  |  |  |  |  | TuABCC-31 | tetur14g02300 | TcABCC-8A | 6467 | XP_969997.2 |  |  |  |
|  |  |  |  |  |  |  | TuABCC-32 | tetur14g02310 | TcABCC-8B | 6468 | XP_970068.2 |  |  |  |
|  |  |  |  |  |  |  | TuABCC-33 | tetur14g02320 | TcABCC-9A | 12253 | XP_969354.2 |  |  |  |
|  |  |  |  |  |  |  | TuABCC-34 | tetur14g02330 | TcABCC-9B | 10962 | XP_969737.2 |  |  |  |
|  |  |  |  |  |  |  | TuABCC-35 | tetur16g03480 | TcABCC-9C | 11800 | XP_001813826.1 |  |  |  |
|  |  |  |  |  |  |  | TuABCC-36 | tetur23g02452 |  |  |  |  |  |  |
|  |  |  |  |  |  |  | TuABCC-38 | tetur25g01780 |  |  |  |  |  |  |
|  |  |  |  |  |  |  | TuABCC-37 | tetur28g01950 |  |  |  |  |  |  |
|  |  |  |  |  |  |  | TuABCC-39 | tetur40g00010 |  |  |  |  |  |  |
|  |  |  |  |  |  |  |  |  |  |  |  |  |  |  |
|  |  |  |  |  |  |  |  |  |  |  |  |  |  |  |
|  |  |  |  |  |  |  |  |  |  |  |  |  |  |  |
|  |  |  |  |  |  |  |  |  |  |  |  |  |  |  |
|  |  |  |  |  |  |  |  |  |  |  |  |  |  |  |
|  |  |  |  |  |  |  |  |  |  |  |  |  |  |  |
| D | Dappu1_347330 EFX83241.1 | C44B7.8 | AAA68339 | CG12703 AAF49018 | ABCD1 | NP_000024.2 | TuABCD-01 | tetur05g06640 | TcABCD-6A | 15333 | XP_971218.1 | Lsa.10176 | HACA01011675 | |
|  | Dappu1_347326 EFX75398.1 | C44B7.9 | AAA68340 | CG2316 AAF59367 | ABCD2 | NP_005155.1 | TuABCD-02 | tetur35g01360 | TcABCD-6A | 12277 | XP_971649.2 | Lsa.5856 | HACA01006738 | |
|  | Dappu1_303977 EFX65057 | C54G10.3 | CAA99810 |  | ABCD3 | AAH68509.1 |  |  |  |  |  |  |  |  |
|  |  | T02D1.5 | CAB0590 |  | ABCD4 | CAG33385.1 |  |  |  |  |  |  |  |  |
|  |  | T10H9.5 | AAC19238 |  |  |  |  |  |  |  |  |  |  |  |
|  |  |  |  |  |  |  |  |  |  |  |  |  |  |  |
|  |  |  |  |  |  |  |  |  |  |  |  |  |  |  |
|  |  |  |  |  |  |  |  |  |  |  |  |  |  |  |
|  |  |  |  |  |  |  |  |  |  |  |  |  |  |  |
|  |  |  |  |  |  |  |  |  |  |  |  |  |  |  |
|  |  |  |  |  |  |  |  |  |  |  |  |  |  |  |
|  |  |  |  |  |  |  |  |  |  |  |  |  |  |  |
|  |  |  |  |  |  |  |  |  |  |  |  |  |  |  |
|  |  |  |  |  |  |  |  |  |  |  |  |  |  |  |
|  |  |  |  |  |  |  |  |  |  |  |  |  |  |  |
| E | Dappu1_189585 EFX66734.1 | Y39E4B.1 | CAB54424 | CG5651 AAF50342 | ABCE1 | NP_002931.2 | TuABCE-01 | tetur30g01400 | TcABCE-3A | 10519 | XP_968009.1 | Lsa.1035* | HACA01001204 HACA01001205 | |
|  |  |  |  |  |  |  |  |  |  |  |  |  |  |  |
|  |  |  |  |  |  |  |  |  |  |  |  |  |  |  |
| F | Dappu1_304799 EFX88800.1 | F18E2.2 | CAA99835 | CG1703 AAF48069 | ABCF1 | BAD92801.1 | TuABCF-01 | tetur20g02610 | TcABCF-2A | 4420 | XP_971562.1 | Lsa.9678 | HACA01011095 | |
|  | Dappu1_347357 EFX73813.1 | F42A10.1 | AAA19072 | CG9281 AAF48493 | ABCF2 | NP_009120.1 | TuABCF-02 | tetur29g00620 | TcABCF-5A | 13884 | XP_966990.1 | Lsa.8082 | HACA01009266 | |
|  | Dappu1_347354 EFX69544.1 | T27E9.7 | CAB04880 | CG9330 AAF49142 | ABCF3 | NP_060828.2 | TuABCF-03 | tetur32g00490 | TcABCF-9A | 11927 | XP_972814.1 | Lsa.20458 | HACA01023315 | |
|  | Dappu1_347363 EFX66171.1 |  |  |  |  |  | TuABCF-04 | tetur11g02160 |  |  |  |  |  |  |
|  |  |  |  |  |  |  |  |  |  |  |  |  |  |  |
|  |  |  |  |  |  |  |  |  |  |  |  |  |  |  |
|  |  |  |  |  |  |  |  |  |  |  |  |  |  |  |
|  |  |  |  |  |  |  |  |  |  |  |  |  |  |  |
|  |  |  |  |  |  |  |  |  |  |  |  |  |  |  |
|  |  |  |  |  |  |  |  |  |  |  |  |  |  |  |
|  |  |  |  |  |  |  |  |  |  |  |  |  |  |  |
|  |  |  |  |  |  |  |  |  |  |  |  |  |  |  |
|  |  |  |  |  |  |  |  |  |  |  |  |  |  |  |
|  |  |  |  |  |  |  |  |  |  |  |  |  |  |  |
|  |  |  |  |  |  |  |  |  |  |  |  |  |  |  |
| G | Dappu1_312940 EFX86710.1 | C05D10.3 | AAA20989 | CG2969 Atet AAF51027 | ABCG1/WHITE1 | AAC51098 | TuABCG-01 | tetur01g16280 | TcABCG-4A | 7470 | XP_971210.2 | Lsa.2606* | HACA01003008 HACA01003009 | |
|  | Dappu1_312948 EFX86708.1 | C10C6.5 | CAB05682 | CG17632 Brown AAF47020 | ABCG2/BCRP | XP_032425 | TuABCG-02 | tetur01g16290 | TcABCG-4B | 7467 | XP_971681.1 | Lsa.25615 | HACA01029057 | |
|  | Dappu1_312949 EFX86707.1 | C16C10.12 | CAA86750 | CG11069 AAF56361 | ABCG4/WHITE2 | NP_071452 | TuABCG-03 | tetur02g11400 | TcABCG-4C | 7466 | XP_001813184.1 |  |  |  |
|  | Dappu1_312951 EFX86600.1 | F02E11.1 | AAB66050 | CG17646 AAF51341 | ABCG5 | AF320293 | TuABCG-04 | tetur03g04350 | TcABCG-4D | 8293 | XP_973458.1 |  |  |  |
|  | Dappu1_312950 EFX86599.1 | F19B6.4 | CAA93461 | CG31121 NP_733058 | ABCG8 | AF320294 | TuABCG-05 | tetur04g04550 | TcABCG-4E |  | XP_001811847.1 |  |  |  |
|  | Dappu1_222011 EFX86598.1 | T26A5.1 | AAC77504 | CG3164 AAF51548 |  |  | TuABCG-06 | tetur05g05440 | TcABCG-4F | 7074 | XP_971735.1 |  |  |  |
|  | Dappu1_312942 EFX86593.1 | Y47D3A.11 | CAB57891 | CG31689 NP_722827 |  |  | TuABCG-07 | tetur06g05430 | TcABCG-4G | 8454 | XP_973493.1 |  |  |  |
|  | Dappu1_347419 EFX86592.1 | Y49E10.9 | CAB11549 | CG32091 NP_729728 |  |  | TuABCG-08 | tetur09g01930 | TcABCG-4H | 7047 | XP_973526.1 |  |  |  |
|  | Dappu1_347409 EFX84701.1 | Y42G9A.6 | AAF60554 | CG3327 AAF51122 |  |  | TuABCG-09 | tetur09g02000 | TcABCG-8A | 5701 | XP_975214.2 |  |  |  |
|  | Dappu1_314702 EFX84700.1 |  |  | CG4822 AAF51552 |  |  | TuABCG-10 | tetur19g01160 | TcABCG-9A | 11998 | XP_968696.1 |  |  |  |
|  | Dappu1_347393 EFX84423.1 |  |  | CG5853 AAF52835 |  |  | TuABCG-11 | tetur33g01719 | TcABCG-9B | 11997 | NP_001034521.1 |  |  |  |
|  | Dappu1_315707 EFX83518.1 |  |  | CG9663 AAF51130 |  |  | TuABCG-12 | tetur37g01090 | TcABCG-9C | 11713 | XP_968472.1 |  |  |  |
|  | Dappu1_347377 EFX83517.1 |  |  | CG9664 AAF51131 |  |  | TuABCG-13 | tetur17g02510 | TcABCG-9D | 12778 | XP_968555.1 |  |  |  |
|  | Dappu1_347380 EFX83516.1 |  |  | CG4314 Scarlet AAF49455 |  |  | TuABCG-14 | tetur11g00520 |  |  |  |  |  |  |
|  | Dappu1_320907 EFX78013.1 |  |  | CG2759 White AAF45826 |  |  | TuABCG-15 | tetur02g13710 |  |  |  |  |  |  |
|  | Dappu1_320906 EFX77993.1 |  |  |  |  |  | TuABCG-16 | tetur09g01980 |  |  |  |  |  |  |
|  | Dappu1_347412 EFX71588.1 |  |  |  |  |  | TuABCG-17 | tetur09g01960 |  |  |  |  |  |  |
|  | Dappu1_347524 EFX71587.1 |  |  |  |  |  | TuABCG-18 | tetur09g01990 |  |  |  |  |  |  |
|  | Dappu1_327299 EFX71283.1 |  |  |  |  |  | TuABCG-19 | tetur11g01800 |  |  |  |  |  |  |
|  | Dappu1_258299 EFX69628.1 |  |  |  |  |  | TuABCG-20 | tetur09g01970 |  |  |  |  |  |  |
|  | Dappu1_300887 EFX69652.1 |  |  |  |  |  | TuABCG-21 | tetur02g11270 |  |  |  |  |  |  |
|  | Dappu1_347416 EFX68572.1 |  |  |  |  |  | TuABCG-22 | tetur09g01950 |  |  |  |  |  |  |
|  | Dappu1_347444 EFX67564.1 |  |  |  |  |  | TuABCG-23 | tetur17g03970 |  |  |  |  |  |  |
|  | Dappu1_347523 EFX62699.1 |  |  |  |  |  |  |  |  |  |  |  |  |  |
| H | Dappu1_46780 EFX84774.1 | C56E6.1 | AAA811093 | CG11147 AAF52284.2 |  |  | TuABCH-01 | tetur01g03530 | TcABCH-9A | 12169 | XP_973444.1 | Lsa.12984 | HACA01014872 | |
|  | Dappu1_99394 EFX84773.1 | C56E6.5 | AAA81094 | CG33970 NP_001034071.1 |  |  | TuABCH-02 | tetur01g05940 | TcABCH-9B | 12512 | XP_967359.1 | Lsa.14023 | HACA01016058 | |
|  | Dappu1_347465 EFX84768.1 |  |  | CG9990 AAF56807.1 |  |  | TuABCH-03 | tetur01g05970 | TcABCH-9C | 11755 | XP_974932.1 | Lsa.21408 | HACA01024391 | |
|  | Dappu1_197573 EFX79371.1 |  |  |  |  |  | TuABCH-04 | tetur03g03080 |  |  |  | Lsa.23267 | HACA01026477 | |
|  | Dappu1_104532 EFX79372.1 |  |  |  |  |  | TuABCH-05 | tetur03g05300 |  |  |  | Lsa.23269* | HACA01026479 HACA01026480 | |
|  | Dappu1_197993 EFX78467.1 |  |  |  |  |  | TuABCH-06 | tetur04g06390 |  |  |  |  |  |  |
|  | Dappu1_347474 EFX78468.1 |  |  |  |  |  | TuABCH-07 | tetur05g05000 |  |  |  |  |  |  |
|  | Dappu1_347478 EFX71371.1 |  |  |  |  |  | TuABCH-08 | tetur07g05200 |  |  |  |  |  |  |
|  | Dappu1_201766 EFX71377.1 |  |  |  |  |  | TuABCH-09 | tetur12g03340 |  |  |  |  |  |  |
|  | Dappu1_347450 EFX70383.1 |  |  |  |  |  | TuABCH-10 | tetur12g03910 |  |  |  |  |  |  |
|  | Dappu1_328125 EFX70382.1 |  |  |  |  |  | TuABCH-11 | tetur13g02010 |  |  |  |  |  |  |
|  | Dappu1_328127 EFX70380.1 |  |  |  |  |  | TuABCH-12 | tetur13g02060 |  |  |  |  |  |  |
|  | Dappu1_228828 EFX69049.1 |  |  |  |  |  | TuABCH-13 | tetur18g00230 |  |  |  |  |  |  |
|  | Dappu1_332183 EFX66472.1 |  |  |  |  |  | TuABCH-14 | tetur19g01780 |  |  |  |  |  |  |
|  | Dappu1_340396 EFX61237.1 |  |  |  |  |  | TuABCH-15 | tetur21g00940 |  |  |  |  |  |  |
|  |  |  |  |  |  |  | TuABCH-16 | tetur26g02620 |  |  |  |  |  |  |
|  |  |  |  |  |  |  | TuABCH-17 | tetur28g00780 |  |  |  |  |  |  |
|  |  |  |  |  |  |  | TuABCH-18 | tetur28g00870 |  |  |  |  |  |  |
|  |  |  |  |  |  |  | TuABCH-19 | tetur30g00890 |  |  |  |  |  |  |
|  |  |  |  |  |  |  | TuABCH-20 | tetur32g01710 |  |  |  |  |  |  |
|  |  |  |  |  |  |  | TuABCH-21 | tetur36g00240 |  |  |  |  |  |  |
|  |  |  |  |  |  |  | TuABCH-22 | tetur36g00630 |  |  |  |  |  |  |
|  |  |  |  |  |  |  |  |  |  |  |  |  |  |  |
|  |  |  |  |  |  |  |  |  |  |  |  |  |  |  |
|  |  |  |  |  |  |  |  |  |  |  |  |  |  |  |
|  |  |  |  |  |  |  |  |  |  |  |  |  |  |  |
